# One-Pot Diastereoselective Synthesis of Pyrrolopiperazine-2,6-diones by a Ugi/Nucleophilic Substitution/N-Acylation Sequence

**DOI:** 10.1021/acs.joc.2c00694

**Published:** 2022-06-27

**Authors:** Beatriz González-Saiz, Israel Carreira-Barral, Pablo Pertejo, Javier Gómez-Ayuso, Roberto Quesada, María García-Valverde

**Affiliations:** Departamento de Química, Facultad de Ciencias, Universidad de Burgos, 09001, Burgos, Spain

## Abstract

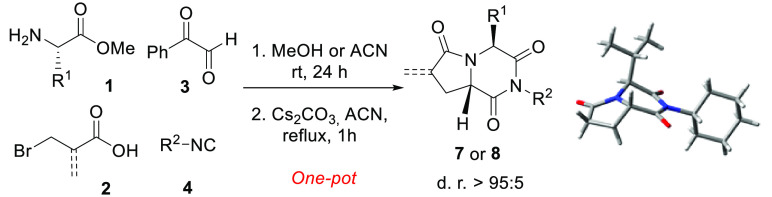

The diastereoselective synthesis of two families of pyrrolopiperazine-2,6-diones is presented. These compounds were prepared by one-pot Ugi/nucleophilic substitution/N-acylation/debenzoylation/(elimination) sequences. This novel route provides straightforward access to a wide variety of pyrrolopiperazine-2,6-diones with high chemical yields and complete diastereoselectivities. The proposed synthetic strategy poses a significant improvement compared to the syntheses of pyrrolopiperazine-2,6-diones previously described, as it allows introduction of different substituents to the C4 position and the diastereoselective generation of a new stereogenic center on the bridgehead carbon (C8a).

The development of new and efficient syntheses of N-heterocycles is of paramount importance because of their relevance in the pharmaceutical and fine chemicals industries.^1^ Such importance relies on the fact that the structures of many of these systems are found in molecules displaying biological activity.^2^ This is the case of fused bicyclic piperazines, found in various natural products presenting antifungal, antibacterial, anxiolytic, or antitumoral activities.^3^ Among the different methodologies described to synthesize fused heterocycles, as pyrrolopiperazines,^3^ multicomponent reactions (MCR) represent an interesting strategy to address the access to these systems.^4^

Regarding pyrrolo-2,5-diketopiperazines, different methodologies based on the Ugi reaction have been described, for instance the Ugi/deprotection/cyclization sequence (UDC),^5^ the Ugi four-center three-component reaction (U-4C-3CR),^6^ and the Joullié–Ugi/postcondensation sequence.^7^ On the other hand, pyrrolo-2,6-diketopiperazines can be prepared through a Ugi five-center four-component reaction (U-5C-4CR) followed by a postcondensation step. In this case, the proline is employed as a doubly functionalized reactant, with the alcohol used as solvent acting as the fourth component. This leads to a 1,1′-diiminodicarboxylic derivative which affords the corresponding piperazine after treatment with a strong base.^8^

Another interesting family of pyrrolodiketopiperazines are those in which the pyrrolo nucleus is a γ-lactam. However, although the enantiopure form of their 2,5-diketopiperazine derivatives can be easily synthesized starting from glutamic acid and other α-amino acids,^9^ the synthesis of enantiopure 2,6-diketopiperazine–pyrrolidinone systems is challenging. Indeed, although the methodologies based on the Ugi reaction are chemically efficient, their characteristic low diastereoselectivity represents an important drawback of these approaches.^10^ Thus, Ciufolini et al. have reported the synthesis of these pyrrolopiperazine-2,6-diones by a two-step sequence, a Ugi five-center four-component reaction (U-5C-4CR), followed by the cyclization promoted by trifluoroacetic acid, yielding products with diastereoselectivities ranging from 10:1 to 1.5:1 (Scheme 1a).^11^ Analogous systems have been described by Ugi, starting from γ-ketoacids and α-aminoesters,^12^ although the stereochemical aspect is, again, a major issue (Scheme 1b). Moreover, the synthesis of the nonsubstituted C8a analogue following this strategy is an extremely expensive alternative.^13^ To the best of our knowledge, only one highly diastereoselective synthetic methodology furnishing pyrrolopiperazine-2,6-diones has been reported that fulfills these conditions, a five-step route with no multicomponent reaction being involved.^14^ However, this path presents an important shortcoming: the substituent introduced in the C4 position of the molecule must be an aromatic moiety, because they are introduced through an electrophilic aromatic substitution (Scheme 1c).

**Scheme 1:**
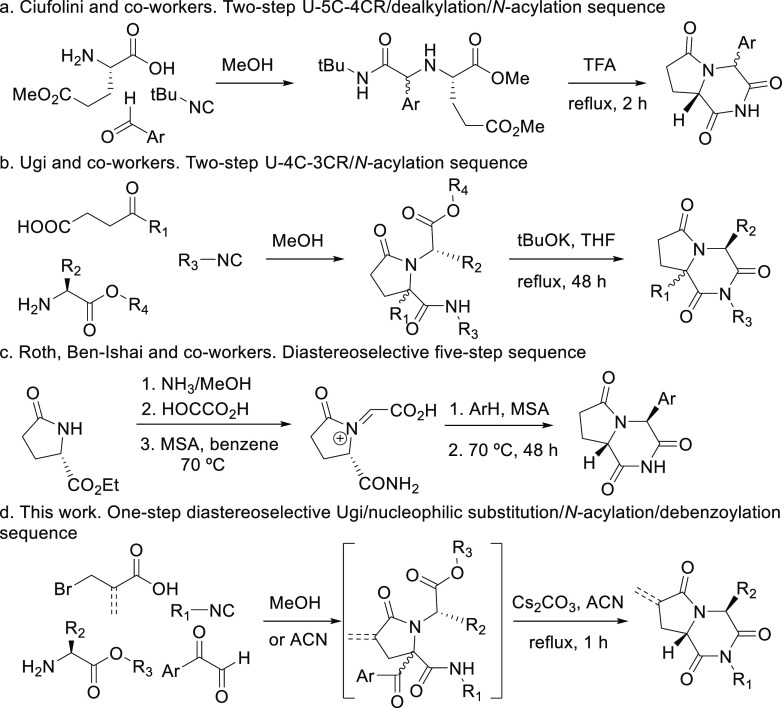
Synthesis of 2,6-Diketopiperazine–Pyrrolidinone-Fused Systems

Prompted by the promising biological activity of 2,6-diketopiperazine–pyrrolidinone fused systems,^15^ herein we present a novel synthetic strategy for the synthesis of this class of compounds that improves those currently described and overcomes some of their limitations. This route allows high chemical yields and an almost quantitative diastereoselectivity of diketopiperazine–pyrrolidinone derivatives bearing an unsubstituted C8a position and different substituents in the C4 position (Scheme 1d). Given the interest in the α-alkylidene-γ-lactam scaffold in the development of compounds with biological properties as Michael acceptors toward bionucleophiles,^16^ α-methylidene-γ-lactams fused with 2,6-diketopiperazines, systems which have not been described so far, were also synthesized following a similar strategy.

The proposed synthetic path is based on a Ugi/nucleophilic substitution sequence, which leads to pyrrolidin-2-ones; a subsequent N-acylation followed by a debenzoylation step would lead to the desired pyrrolopiperazine-2,6-diones. Initially, we employed the three α-aminoesters **1a**–**c** to study the viability of the outlined route, together with 3-bromopropionic acid (**2a**), phenylglyoxal (**3**), and cyclohexyl isocyanide (**4a**) (Scheme 2). The reason behind the selection of phenylglyoxal as the carbonyl component is that the benzoyl group favors the enol tautomer,^17^ promoting cyclization through a C-alkylation reaction; besides, this group can be easily removed in a subsequent step by a retro-Claisen-like reaction.^18^ So, a solution of the commercial hydrochloride form of the corresponding α-aminoesters **1a**–**c** was treated with potassium hydroxide in methanol for 10 min. Subsequently, 3-bromopropionic acid (**2**), phenylglyoxal (**3**), and cyclohexyl isocyanide (**4a**) were added, and the mixture was stirred for 24 h. The spontaneous cyclization of Ugi adducts **5** afforded the corresponding pyrrolidin-2-one **6** with a poor diastereoselectivity (Table 1). The glycine derivative required longer reaction times due to the lower solubility of the intermediate Ugi adduct **5a** in methanol or treatment with a catalytic amount of cesium carbonate (Scheme 2).

**Scheme 2:**
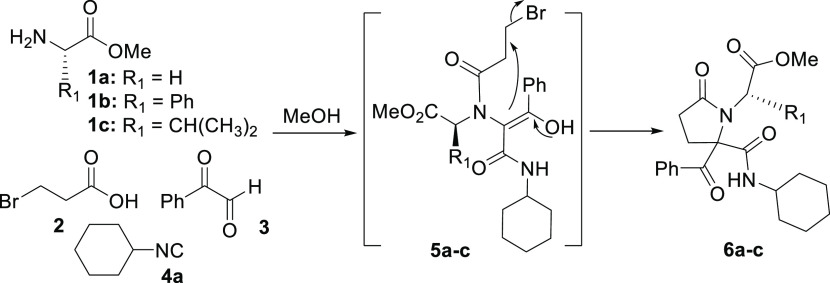
Synthesis of Pyrrolidin-2-ones through a Ugi/Cyclization Sequence

**Table 1:** Results for the Synthesis of Pyrrolidin-2-ones **6**

entry	**1** (R^1^)	**6** (%)	dr^a^
**1**	**1a** (H)	**6a** (54)^b^^,^^c^	–
**2**	**1b** (Ph)	**6b** (41)^d^ (65)^e^	51:49
**3**	**1c** (CH(CH_3_)_2_)	**6c** (35)^d^ (53)^e^	50:50

a Determined by ^1^H NMR spectroscopy in the reaction mixture.

b Ugi adduct **5a** was the only product observed after 24 h (78% yield).

c Yield referred to the conversion of the Ugi adduct to the pyrrolidinone. Conversion is achieved by refluxing **5a** in acetonitrile with a catalytic amount of cesium carbonate for 1 h.

d Chemical yield of the major diastereomer after purification by column chromatography.

e Chemical yield of the diastereomers mixture after purification by column chromatography.

Upon isolation of pyrrolidinones **6a**–**c**, they were treated with cesium carbonate (2 equiv) in acetonitrile and heated to reflux for 1 h. Surprisingly, despite the low nucleophilicity of the carbonate anion, debenzoylated pyrrolopiperazine-2,6-diones **7a**–**c** were the only products detected, formed in a high chemical yield (Scheme 3). A remarkable characteristic of these reactions was their stereochemical outcome when chiral α-aminoesters **1b**,**c** were employed, because regardless of the diastereomeric purity of pyrrolidin-2-one derivatives **6b**,**c**, only one diastereomer of the corresponding pyrrolopiperazine-2,6-diones **7b**,**c** was observed (Scheme 3, Table 2).

**Scheme 3:**
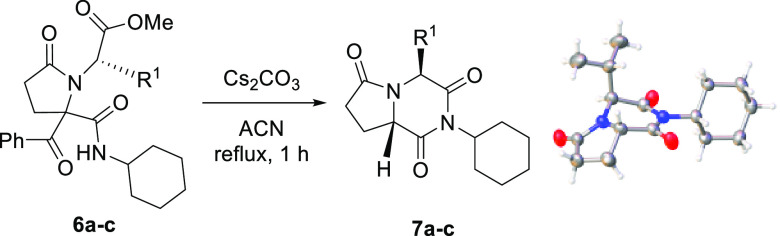
Diastereoselective Synthesis of Pyrrolidinone-Fused Piperazine-2,6-diones **7** and X-ray Molecular Structure of Pyrrolopiperazine-2,6-dione **7c** The Olex2 plot is at the 30% probability level.

**Table 2:** Results for the Synthesis of Pyrrolidinone-Fused Piperazine-2,6-diones **7** from Pyrrolidinones **6**

entry	**6** (R^1^)	**7** (%)	dr^a^
**1**	**6a** (H)	**7a** (84)	–b
**2**	**6b** (Ph)	**7b** (89)	>95:5
**3**	**6c** (CH(CH_3_)_2_)	**7c** (82)	>95:5

a Determined by ^1^H NMR spectroscopy in the reaction mixture.

b Racemic mixture.

The ^1^H NMR spectra of pyrrolopiperazine-2,6-diones **7a**–**c** show a triplet of triplets at ca. 4.5 ppm, corresponding to the proton of the cyclohexane’s methine group, which confirms that cyclization took place through the nitrogen atom coming from the isocyanide, together with a signal around 4.3 ppm, due to the proton linked to C8a; in addition, no signals attributable to the benzoyl group are observed, in agreement with the occurrence of a debenzoylation process (see SI). The stereochemistry of the obtained pyrrolopiperazine-2,6-diones was determined through NOESY experiments and confirmed by single-crystal X-ray diffraction analysis of pyrrolopiperazine-2,6-dione **7c** (Scheme 3). In this way, the absolute configuration of pyrrolopiperazine-2,6-diones **7b**,**c** was confirmed to be (4*S*,8a*S*).

Both the chemical and stereochemical results can be explained by a mechanism according to which, initially, an N-anion (**A** in Scheme 4) would be formed, that is explained by the presence of water traces in the solvent employed in the reaction. In this way, the intramolecular N-acylation would take place, resulting in the diketopiperazine–pyrrolidinone fused system **B**, with the concomitant removal of the methoxide group. At this point, it is difficult to prevent the retro-Claisen reaction because this methoxide group would attack the benzoyl group, yielding methyl benzoate, as could be detected in the raw product by ^1^H NMR spectroscopy; furthermore, this step would be favored by the thermodynamic stability of enolate **D.** This would be the key step in the stereoreochemical outcome, because the stereocenter of the former pyrrolidin-2-one **6** would have been destroyed. Protonation of enolate **D** would be controlled by the stereochemistry of the chiral center coming from the corresponding α-aminoester and would generate the most stable diastereoisomer of pyrrolopiperazine-2,6-diones **7b**,**c** (Scheme 4). To examine this, DFT quantum chemical calculations for the epimers of **7c** on C8a were carried out using Gaussian 16.^19^ After full optimization of the geometries of both species at the B3LYP/6-31G** level, the calculated energy for (4*S*,8a*S*)-**7c** was 5.02 kcal·mol^–1^ lower than that obtained for the (4*S*,8a*R*)-**7c** epimer (see SI). The higher stability of the former is in agreement with the stereochemistry determined for these compounds in solution by NOESY experiments and with that observed for **7c** in the solid state. This result could be explained by the steric hindrance exerted by the R^1^ substituent, which constrained the bicyclic system in the (4*S*,8a*R*)-**7c** epimer.

**Scheme 4:**
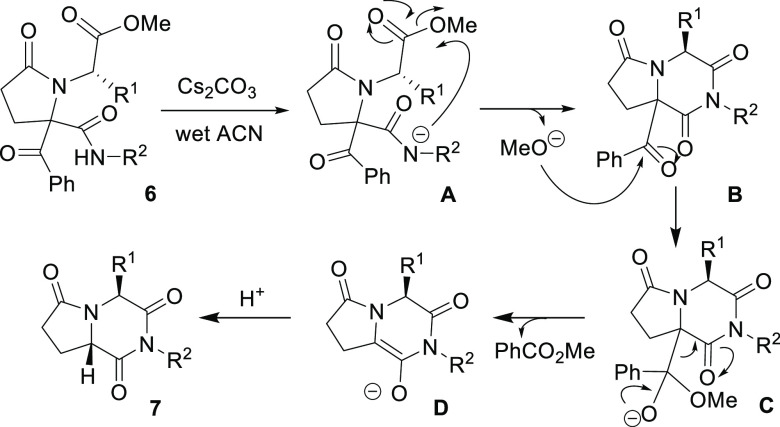
Proposed Mechanism for the Diastereoselective Synthesis of Pyrrolopiperazine-2,6-diones **7**

Bearing in mind that the configuration of C5 in pyrrolidin-2-ones **6** does not determine the stereochemistry of the final pyrrolopiperazine-2,6-diones **7**, the synthetic route leading to them was performed in one pot, without isolating the pyrrolidin-2-one intermediates **6**. Thus, initially, the synthesis of pyrrolidin-2-ones **6** in methanol was conducted and, after removing the solvent, without any further purification, the residue was dissolved in acetonitrile and treated with cesium carbonate. Analysis of the raw product by ^1^H NMR spectroscopy revealed the formation of only one diastereomer of pyrrolopiperazine-2,6-diones **7b**,**c**, but above all, the global chemical yield was remarkably improved. The scope of this one-pot two-step sequence was then assayed with other α-aminoesters and isocyanides and, as expected, all pyrrolopiperazine-2,6-diones **7** were obtained in high yields and in their enantiopure form except for glycine derivative **7a**, which was isolated as a racemic mixture. (Scheme 5, Table 3). Finally, an attempt was made to carry out this sequence using a single solvent to simplify the experimental procedure, adding cesium carbonate to the reaction mixture without removing the solvent. Thus, although the use of methanol in both stages afforded complex mixtures, the use of acetonitrile allowed the isolation of pyrrolopiperazines **7**. However, chemical yields were significantly lower when only one solvent was employed (Table 3, entries 3, 4 vs 5, 6).

**Scheme 5:**
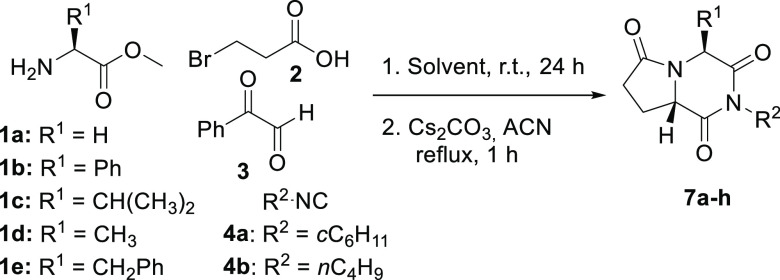
One-Pot Two-Step Sequence for the Synthesis of Pyrrolidinone-Fused 2,6-Diketopiperazines **7**

**Table 3:** Results for the Synthesis of Pyrrolidinone-Fused 2,6-Diketopiperazines **7** by a One-Pot Two-Step Sequence

entry	**1** (R^1^)	**4** (R^2^)	solvent^a^	**7** (%)^b^	dr^c^
**1**	**1a** (H)	**4a** (*c*C_6_H_11_)	MeOH	**7a** (78)	–
**2**	**1b** (Ph)	**4a** (*c*C_6_H_11_)	MeOH	**7b** (86)	>95:5
**3**	**1c** (CH(CH_3_)_2_)	**4a** (*c*C_6_H_11_)	MeOH	**7c** (79)	>95:5
**4**	**1c** (CH(CH_3_)_2_)	**4a** (*c*C_6_H_11_)	ACN	**7c** (58)	>95:5
**5**	**1d** (CH_3_)	**4a** (*c*C_6_H_11_)	MeOH	**7d** (71)	>95:5
**6**	**1d** (CH_3_)	**4a** (*c*C_6_H_11_)	ACN	**7d** (56)	>95:5
**7**	**1e** (CH_2_Ph)	**4a** (*c*C_6_H_11_)	MeOH	**7e** (74)	>95:5
**8**	**1b** (Ph)	**4b** (*n*C_4_H_9_)	MeOH	**7f** (75)	>95:5
**9**	**1c** (CH(CH_3_)_2_)	**4b** (*n*C_4_H_9_)	MeOH	**7g** (81)	>95:5
**10**	**1e** (CH_2_Ph)	**4b** (*n*C_4_H_9_)	MeOH	**7h** (69)	>95:5

a Solvent employed in the first stage.

b Chemical yield after purification.

c Determined by ^1^H NMR spectroscopy in the reaction mixture.

Hence, it was confirmed that the purification and isolation of the pyrrolidin-2-one intermediate is not necessary, and that the reaction can be conducted in a one-pot two-step sequence, providing excellent stereochemical results. These results prompted us to apply this one-pot methodology to the synthesis of 2,6-diketopiperazines fused with α-methylidene-γ-lactams to introduce a methylene group as a convenient Michael acceptor toward bionucleophiles. Initially, for this purpose, 2-(bromomethyl)acrylic acid **2b** was selected, to introduce the methylidene group. So, the strategy previously optimized was applied but, although the α-methylidene-γ-lactams fused with 2,6-diketopiperazines **8** were obtained, yields were quite low due to the poor results obtained in the Ugi reaction, as confirmed by the analysis of the ^1^H NMR spectra of the raw products obtained in this step. With the aim of improving these results, a different carboxylic acid was chosen, 3-bromo-2-(bromomethyl)propionic acid **2c**. A priori it seemed that an additional step would be required to generate the double bond, so it was surprising to see that, after performing the Ugi reaction, treatment of the crude with cesium carbonate afforded α-methylidene-γ-lactams **8** in high yields and with complete diastereoselectivity, no additional step being necessary to create the double bond. This is a consequence of the elimination of hydrogen bromide, which took place along with the N-acylation/debenzoylation step (Scheme 6, Table 4). The absolute configuration of pyrrolopiperazine-2,6-diones **8b**,**e** was assigned as (4*S*,8a*S*) on the basis of NOESY experiments. Again, computational studies for the epimers of **8c** on C8a confirmed the higher stability of the (4*S*,8a*S*) diastereoisomer, with a difference of energy of 5.19 kcal·mol^–1^ between epimers (see SI).

**Scheme 6:**
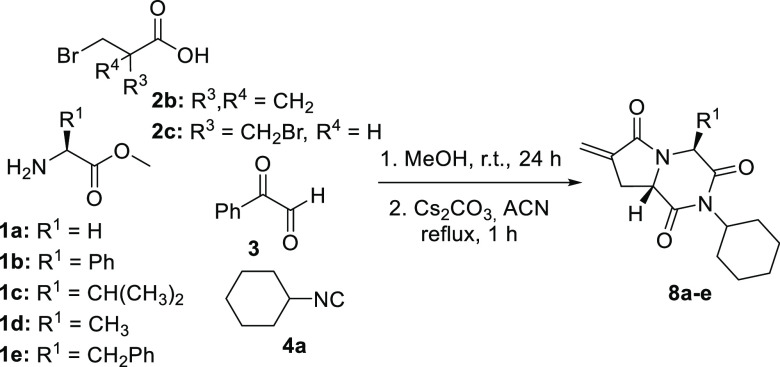
One-Pot Two-Step Sequence for the Synthesis of α-Methylidene-γ-lactam-Fused 2,6-Diketopiperazines **8**

**Table 4:** Results for the Synthesis of α-Methylidene-γ-lactam-Fused 2,6-Diketopiperazines **8** by a One-Pot Two-Step Sequence

entry	**1** (R^1^)	**8** (%)^a^	dr^b^
**1**	**1a** (H)	**8a** (33)^c^ (85)^d^	–
**2**	**1b** (Ph)	**8b** (77)	>95:5
**3**	**1c** (CH(CH_3_)_2_)	**8c** (38)^c^ (83)^d^	>95:5
**4**	**1d** (CH_3_)	**8d** (61)	>95:5
**5**	**1e** (CH_2_Ph)	**8e** (71)	>95:5

a Chemical yield after purification.

b Determined by ^1^H NMR spectroscopy in the reaction mixture.

c Starting from 2-(bromomethyl)acrylic acid.

d Starting from 3-bromo-2-(bromomethyl)propionic acid.

Thus, although atom economy was higher employing 2-(bromomethyl)acrylic acid, meaning that the double bond was introduced in the reactant and not generated during the reaction, the use of 3-bromo-2-(bromomethyl)propionic acid is undoubtedly a better choice as the overall yield is clearly higher. Therefore, the proposed one-pot two-step strategy proves itself a very powerful tool for the rapid and affordable diastereoselective synthesis of biologically relevant pyrrolopiperazine-2,6-diones.

In summary, a novel and completely diastereoselective one-pot methodology to synthesize pyrrolopiperazine-2,6-diones was presented, employing a simple Ugi/nucleophilic substitution/N-acylation/debenzoylation/(elimination) sequence. This synthetic strategy represents a significant improvement over those described in the literature so far, as it allows introduction of a number of alkyl and aryl substituents to the C4 position, owing to the broad variety of commercially available α-aminoesters and the controlled generation of a new stereogenic center on the bridgehead carbon (C8a), starting from simple and inexpensive reagents, and reaching high chemical yields and quantitative diastereoselectivities, along with the possibility of introducing a methylene group as a Michael acceptor in the pyrrolidinone ring.

## Experimental Section

### General Methods

All reagents and solvents were purchased and used without any further purification. Melting points are not corrected. Optical rotations were measured on a Zeiss D-7082 polarimeter in a 1 dm cell, and concentrations are given in g/100 mL. ^1^H and ^13^C NMR spectra were recorded in CDCl_3_ at 300 and 75 MHz, respectively, on a Varian Mercury 300 system or a Bruker Avance III HD system; DEPT-135 experiments were conducted to assign carbon-13 signals. Chemical shifts are reported in parts per million with respect to residual solvent protons and coupling constants in hertz. High resolution mass spectra were recorded on a 6545 Q-TOF Agilent LC-MS mass spectrometer (positive electrospray ionization mode, ESI (+)). X-ray diffraction studies were performed on a Bruker D8 VENTURE diffractometer.

### Procedure for the Synthesis of Ugi Adduct **5a**

Glycine methyl ester hydrochloride **1a** (0.250 g, 2 mmol, 1.0 equiv) was treated with potassium hydroxide (0.101 g, 1.8 mmol, 0.9 equiv) in methanol (10 mL), and the mixture was sonicated for 10 min. Subsequently, 3-bromopropionic acid (**2**) (0.306 g, 2 mmol, 1 equiv) was added to the mixture, followed by phenylglyoxal hydrate (**3**) (0.304 g, 2 mmol, 1 equiv) and cyclohexyl isocyanide (**4**) (0.218 g, 2 mmol, 1 equiv). The reaction mixture was stirred at room temperature for 24 h and the obtained precipitate isolated by vacuum filtration, washed with cold methanol, and dried in vacuo.

### (*E*)-Methyl 2-(3-Bromo-*N*-(3-(cyclohexylamino)-1-hydroxy-3-oxo-1-phenylprop-1-en-2-yl)propanamido)acetate (**5a**)

Pink solid. 78% yield, 727 mg. Mp 128–130 °C. ^1^H NMR (300 MHz, CDCl_3_) δ: 15.50 (s, 1H, OH), 8.27 (d, *J* = 7.7 Hz, 1H, NH), 7.43–7.35 (m, 5H), 4.39 (d, *J* = 16.7 Hz, 1H), 3.87–3.71 (m, 1H), 3.73 (s, 3H), 3.55–3.38 (m, 2H), 3.33 (d, *J* = 16.7 Hz, 1H), 2.92–2.71 (m, 2H), 1.97–1.12 (m, 10H). ^13^C{^1^H} NMR (75 MHz, CDCl_3_) δ: 172.3 (C_q_), 171.4 (C_q_), 169.9 (C_q_), 169.5 (C_q_), 133.3 (C_q_), 130.7 (CH_Ar_), 128.8 (CH_Ar_), 127.0 (CH_Ar_), 108.1 (C_q_), 53.3 (CH_2_), 52.8 (CH_3_), 48.8 (CH), 36.0 (CH_2_), 32.9 (CH_2_), 32.4 (CH_2_), 26.1 (CH_2_), 25.4 (CH_2_), 24.9 (CH_2_). HRMS (ESI) *m*/*z*: [M + H]^+^ calcd for C_21_H_28_BrN_2_O_5_ 467.1182; found 467.1186.

### General Procedure for the Synthesis of Pyrrolidin-2-ones

Method A: Synthesis of pyrrolidin-2-one **6a**. Cesium carbonate (0.05 mmol, 0.05 equiv) was added to a suspension of Ugi adduct **5a** (1 mmol, 1 equiv) in acetonitrile (3 mL). The reaction mixture was heated to reflux with a heating block for 1 h, after which the solvent was removed in a rotary evaporator. The raw product was dissolved in chloroform, and the resulting solution was washed with a 1 M HCl aqueous solution. The organic layer was dried over sodium sulfate, filtered, and concentrated to dryness. The residue was purified by column chromatography, employing SiO_2_ as stationary phase and a hexane/ethyl acetate mixture as eluent. Method B: Synthesis of pyrrolidin-2-ones **6b**,**c**. The corresponding α-aminoester hydrochloride **1b**,**c** (2 mmol, 1 equiv) was treated with potassium hydroxide (1.8 mmol, 0.9 equiv) in methanol (10 mL), and the resulting suspension sonicated for 10 min. Subsequently, 3-bromopropionic acid (**2**) (2 mmol, 1 equiv) was added to the reaction mixture, followed by phenylglyoxal **3** (2 mmol) and isocyanide **4** (2 mmol, 1 equiv). The reaction mixture was stirred at room temperature for 24 h and, after removing the solvent under reduced pressure, the crude product was dissolved in chloroform. This solution was washed with a 1 M HCl aqueous solution and then with a 1 M NaOH aqueous solution. The organic layer was dried over sodium sulfate, filtered, and concentrated to dryness. The residue was purified by column chromatography, employing SiO_2_ as stationary phase and a hexane/ethyl acetate mixture as eluent.

### Methyl 2-(2-Benzoyl-2-(cyclohexylcarbamoyl)-5-oxopyrrolidin-1-yl)acetate (**6a**)

Yellow oil (5:1 Hex/EtOAc). 54% yield, 208 mg. ^1^H NMR (300 MHz, CDCl_3_) δ: 7.87–7.84 (m, 2H), 7.55 (tt, *J* = 7.3, 1.2 Hz, 1H), 7.43 (t, *J* = 7.3 Hz, 2H), 7.13 (d, *J* = 7.9 Hz, 1H), 4.31 (d, *J* = 17.6 Hz, 1H), 3.94 (d, *J* = 17.6 Hz, 1H), 3.81–3.69 (m, 1H), 3.74 (s, 3H), 2.71 (dd, *J* = 8.2, 7.5 Hz, 2H), 2.59–2.25 (m, 2H), 1.91–0.98 (m, 10H). ^13^C{^1^H} NMR (75 MHz, CDCl_3_) δ: 196.6 (C_q_), 175.7 (C_q_), 170.3 (C_q_), 167.8 (C_q_), 134.7 (C_q_), 133.3 (CH_Ar_), 129.3 (CH_Ar_), 128.5 (CH_Ar_), 76.7 (C_q_), 52.5 (CH_3_), 49.2 (CH), 44.9 (CH_2_), 32.4 (CH_2_), 32.2 (CH_2_), 29.4 (CH_2_), 28.5 (CH_2_), 25.3 (CH_2_), 24.9 (CH_2_), 24.8 (CH_2_). HRMS (ESI) *m*/*z*: [M + Na]^+^ calcd for C_21_H_26_N_2_O_5_Na 409.1739; found 409.1733.

### (2*S*)-Methyl 2-(2-Benzoyl-2-(cyclohexylcarbamoyl)-5-oxopyrrolidin-1-yl)-2-phenylacetate (**6b**)

Yellow oil (5:1 Hex/EtOAc). 65% yield, 600 mg (as diastereomers mixture). ^1^H NMR (300 MHz, CDCl_3_) δ: 7.72 (d, *J* = 7.4 Hz, 2H), 7.53 (t, *J* = 7.4 Hz, 1H), 7.39 (t, *J* = 7.4 Hz, 2H), 7.30–7.22 (m, 5H), 6.28 (d, *J* = 7.6 Hz, 1H, NH), 5.40 (s, 1H), 3.70 (s, 3H), 3.48–3.34 (m, 1H), 2.77–2.52 (m, 4H), 1.66–0.68 (m, 10H). ^13^C{^1^H} NMR (75 MHz, CDCl_3_) δ: 196.6 (C_q_), 176.6 (C_q_), 169.8 (C_q_), 168.3 (C_q_), 135.2 (C_q_), 134.6 (C_q_), 133.2 (CH_Ar_), 129.4 (CH_Ar_), 129.1 (CH_Ar_), 128.5 (CH_Ar_), 128.3 (CH_Ar_), 76.1 (C_q_), 61.3 (CH), 52.8 (CH_3_), 49.0 (CH), 32.0 (CH_2_), 31.8 (CH_2_), 30.9 (CH_2_), 29.7 (CH_2_), 28.9 (CH_2_), 25.2 (CH_2_), 24.6 (CH_2_). HRMS (ESI) *m*/*z*: [M + H]^+^ calcd for C_27_H_31_N_2_O_5_ 463.2233; found 463.2232.

### (2*S*)-Methyl 2-(2-Benzoyl-2-(cyclohexylcarbamoyl)-5-oxopyrrolidin-1-yl)-3-methylbutanoate (**6c**)

Yellow oil (5:1 Hex/EtOAc). 53% yield, 454 mg (as diastereomers mixture). ^1^H NMR (300 MHz, CDCl_3_) δ: 7.82 (d, *J* = 7.5 Hz, 2H), 7.56 (tt, *J* = 7.5, 1.2 Hz, 1H), 7.42 (t, *J* = 7.5 Hz, 2H), 6.23 (d, *J* = 7.8 Hz, 1H, NH), 3.85–3.78 (m, 1H), 3.76 (s, 3H), 3.70 (d, *J* = 10.2 Hz, 1H), 3.06–2.90 (m, 1H), 2.87–2.56 (m, 2H), 2.45–2.28 (m, 2H), 1.93–1.01 (m, 10H), 0.85 (d, *J* = 6.6 Hz, 3H), 0.84 (d, *J* = 6.7 Hz, 3H). ^13^C{^1^H} NMR (75 MHz, CDCl_3_) δ: 196.9 (C_q_), 175.6 (C_q_), 171.5 (C_q_), 166.9 (C_q_), 133.7 (CH_Ar_), 129.3 (CH_Ar_), 128.5 (CH_Ar_), 78.6 (C_q_), 65.8 (CH), 52.0 (CH_3_), 49.3 (CH), 32.5 (CH_2_), 32.0 (CH_2_), 29.6 (CH_2_), 29.4 (CH_2_), 27.8 (CH), 25.2 (CH_2_), 24.7 (CH_2_), 24.5 (CH_2_), 20.5 (CH_3_), 20.2 (CH_3_). HRMS (ESI) *m*/*z*: [M + H]^+^ calcd for C_24_H_33_N_2_O_5_ 429.2389; found 429.2393.

### General Procedure for the Synthesis of Pyrrolidinone-Fused 2,6-Diketopiperazines

Method A. A mixture of pyrrolidin-2-ones **6a**–**c** (0.2 mmol, 1 equiv) and cesium carbonate (0.4 mmol, 2 equiv) in acetonitrile (3 mL) was stirred and heated to reflux with a heating block for 1 h. Subsequently, the solvent was removed under reduced pressure and the crude product dissolved in chloroform. This solution was washed with water and the organic layer dried over sodium sulfate, filtered, and concentrated to dryness. The residue was purified by column chromatography, employing SiO_2_ as stationary phase and a hexane/ethyl acetate mixture as eluent. Method B. The corresponding α-aminoester hydrochloride **1a**–**e** (2 mmol, 1 equiv) was treated with potassium hydroxide (1.8 mmol, 0.9 equiv) in methanol (10 mL) and the resulting suspension sonicated for 10 min. Subsequently, 3-bromopropionic acid (**2**) (2 mmol, 1 equiv) was added to the reaction mixture, followed by phenylglyoxal **3** (2 mmol, 1 equiv) and isocyanide **4a**,**b** (2 mmol, 1 equiv). The reaction mixture was stirred at room temperature for 24 h, and the solvent was removed under reduced pressure. The raw product was dissolved in acetonitrile (10 mL), treated with cesium carbonate (4 mmol, 2 equiv), heated to reflux with a heating block for 1 h, and henceforth treated as indicated in method A. Method C. The corresponding α-aminoester hydrochloride **1a**–**e** (2 mmol, 1 equiv) was treated with potassium hydroxide (1.8 mmol, 0.9 equiv) in acetonitrile (10 mL) and the resulting suspension sonicated for 10 min. Subsequently, 3-bromopropionic acid (**2**) (2 mmol, 1 equiv) was added to the reaction mixture, followed by phenylglyoxal **3** (2 mmol, 1 equiv) and isocyanide **4** (2 mmol, 1 equiv). The reaction mixture was stirred at room temperature for 24 h, and then cesium carbonate (4 mmol) was added. The mixture was stirred and heated to reflux with a heating block for 1 h. From there, the reaction was conducted according to the procedure described in method A.

### 2-Cyclohexyldihydropyrrolo[1,2-*a*]pyrazine-1,3,6(2*H*,4*H*,7*H*)-trione (**7a**)

Brown oil (2:1 Hex/EtOAc). 78% yield, 390 mg. ^1^H NMR (300 MHz, CDCl_3_) δ: 4.84 (d, *J* = 18.6 Hz, 1H), 4.47 (tt, *J* = 12.3, 3.8 Hz, 1H), 4.30–4.21 (m, 1H), 3.78 (d, *J* = 18.6 Hz, 1H), 2.59–2.32 (m, 4H), 2.25–1.03 (m, 10H). ^13^C{^1^H} NMR (75 MHz, CDCl_3_) δ: 173.0 (C_q_), 170.9 (C_q_), 167.7 (C_q_), 56.9 (CH), 54.0 (CH), 44.0 (CH_2_), 29.3 (CH_2_), 29.1 (CH_2_), 28.6 (CH_2_), 26.3 (CH_2_), 26.2 (CH_2_), 25.1 (CH_2_), 21.4 (CH_2_). HRMS (ESI) *m*/*z*: [M + H]^+^ calcd for C_13_H_19_N_2_O_3_ 251.1396; found 251.1390.

### (4*S*,8a*S*)-2-Cyclohexyl-4-phenyldihydropyrrolo[1,2-*a*]pyrazine-1,3,6(2*H*,4*H*,7*H*)-trione (**7b**)

Pale yellow oil (2:1 Hex/EtOAc). 86% yield, 561 mg. [α]_D_ = −149.8° (*c* = 0.26, MeOH). ^1^H NMR (300 MHz, CDCl_3_) δ: 7.45–7.21 (m, 5H), 6.03 (s, 1H), 4.61 (tt, *J* = 12.2, 3.7 Hz, 1H), 4.23–4.13 (m, 1H), 2.64–2.19 (m, 4H), 1.97–1.03 (m, 10H). ^13^C{^1^H} NMR (75 MHz, CDCl_3_) δ: 173.0 (C_q_), 168.3 (C_q_), 133.6 (C_q_), 129.3 (CH_Ar_), 128.9 (CH_Ar_), 126.8 (CH_Ar_), 56.6 (CH), 54.4 (CH), 54.1 (CH), 29.7 (CH_2_), 29.1 (CH_2_), 28.8 (CH_2_), 26.3 (CH_2_), 26.3 (CH_2_), 25.1 (CH_2_), 22.0 (CH_2_). HRMS (ESI) *m*/*z*: [M + H]^+^ calcd for C_19_H_23_N_2_O_3_ 327.1709; found 327.1706.

### (4*S*,8a*S*)-2-Cyclohexyl-4-isopropyldihydropyrrolo[1,2-*a*]pyrazine-1,3,6(2*H*,4*H*,7*H*)-trione (**7c**)

White solid (2:1 Hex/EtOAc). 79% yield, 461 mg. Mp 73–75 °C. [α]_D_ = +2.65° (*c* = 2.0, MeOH). ^1^H NMR (300 MHz, CDCl_3_) δ: 4.47–4.28 (m, 3H), 2.60–1.97 (m, 5H), 1.79–1.04 (m, 10H), 0.99 (d, *J* = 6.8 Hz, 3H), 0.89 (d, *J* = 6.8 Hz, 3H). ^13^C{^1^H} NMR (75 MHz, CDCl_3_) δ: 173.8 (C_q_), 171.5 (C_q_), 169.5 (C_q_), 59.1 (CH), 55.9 (CH), 53.7 (CH), 31.2 (CH), 29.5 (CH_2_), 29.1 (CH_2_), 28.7 (CH_2_), 26.3 (CH_2_), 26.2 (CH_2_), 25.1 (CH_2_), 23.0 (CH_2_), 19.4 (CH_3_), 19.3 (CH_3_). HRMS (ESI) *m*/*z*: [M + H]^+^ calcd for C_16_H_25_N_2_O_3_ 293.1865; found 293.1861.

### (4*S*,8a*S*)-2-Cyclohexyl-4-methyldihydropyrrolo[1,2-*a*]pyrazine-1,3,6(2*H*,4*H*,7*H*)-trione (**7d**)

Yellow solid (2:1 Hex/EtOAc). 71% yield, 375 mg. Mp 143–145 °C. [α]_D_ = −103.3° (*c* = 0.16, MeOH). ^1^H NMR (300 MHz, CDCl_3_) δ: 4.88 (q, *J* = 7.3 Hz, 1H), 4.47 (tt, *J* = 12.3, 3.7 Hz, 1H), 4.34–4.28 (m, 1H), 2.59–2.27 (m, 4H), 2.26–1.08 (m, 10H), 1.47 (d, *J* = 7.3 Hz, 3H). ^13^C{^1^H} NMR (75 MHz, CDCl_3_) δ: 172.8 (C_q_), 171.2 (C_q_), 171.1 (C_q_), 54.0 (CH), 54.0 (CH), 49.6 (CH), 29.6 (CH_2_), 29.1 (CH_2_), 28.7 (CH_2_), 26.3 (CH_2_), 26.2 (CH_2_), 25.1 (CH_2_), 21.5 (CH_2_), 16.0 (CH_3_). HRMS (ESI) *m*/*z*: [M + H]^+^ calcd for C_14_H_21_N_2_O_3_ 265.1552; found 265.1546.

### (4*S*,8a*S*)-4-Benzyl-2-cyclohexyldihydropyrrolo[1,2-*a*]pyrazine-1,3,6(2*H*,4*H*,7*H*)-trione (**7e**)

Yellow solid (2:1 Hex/EtOAc). 74% yield, 503 mg. Mp 148–150 °C. [α]_D_ = −72.1° (*c* = 0.29, MeOH). ^1^H NMR (300 MHz, CDCl_3_) δ: 7.30–7.23 (m, 3H), 7.14–7.11 (m, 2H), 5.04 (t, *J* = 5.3 Hz, 1H), 4.49 (tt, *J* = 12.2, 3.8 Hz, 1H), 3.41–3.33 (m, 2H), 3.20 (dd, *J* = 13.8, 5.5 Hz, 1H), 2.41–1.08 (m, 14H). ^13^C{^1^H} NMR (75 MHz, CDCl_3_) δ: 173.3 (C_q_), 170.8 (C_q_), 170.4 (C_q_), 135.9 (C_q_), 129.2 (CH_Ar_), 129.0 (CH_Ar_), 127.7 (CH_Ar_), 55.4 (CH), 55.1 (CH), 54.1 (CH), 37.4 (CH_2_), 29.4 (CH_2_), 29.1 (CH_2_), 28.5 (CH_2_), 26.3 (CH_2_), 26.2 (CH_2_), 25.2 (CH_2_), 22.0 (CH_2_). HRMS (ESI) *m*/*z*: [M + H]^+^ calcd for C_20_H_25_N_2_O_3_ 341.1865; found 341.1860.

### (4*S*,8a*S*)-2-Butyl-4-phenyldihydropyrrolo[1,2-*a*]pyrazine-1,3,6(2*H*,4*H*,7*H*)-trione (**7f**)

White solid (3:1 Hex/EtOAc). 75% yield, 455 mg. Mp 89–91 °C. [α]_D_ = +86.3° (*c* = 0.12, MeOH). ^1^H NMR (300 MHz, CDCl_3_) δ: 7.40–7.25 (m, 5H), 6.08 (s, 1H), 4.26–4.21 (m, 1H), 3.88–3.83 (m, 2H), 2.60–2.31 (m, 4H), 1.60–1.50 (m, 2H), 1.35 (sext, *J* = 7.7 Hz, 2H), 0.94 (t, *J* = 7.3 Hz, 3H). ^13^C{^1^H} NMR (75 MHz, CDCl_3_) δ: 173.2 (C_q_), 170.8 (C_q_), 167.9 (C_q_), 133.3 (C_q_), 129.3 (CH_Ar_), 128.9 (CH_Ar_), 126.7 (CH_Ar_), 56.3 (CH), 54.1 (CH), 40.1 (CH_2_), 29.9 (CH_2_), 29.5 (CH_2_), 21.6 (CH_2_), 20.1 (CH_2_), 13.7 (CH_3_). HRMS (ESI) *m*/*z*: [M + H]^+^ calcd for C_17_H_21_N_2_O_3_ 301.1547; found 301.1549.

### (4*S*,8a*S*)-2-Butyl-4-isopropyldihydropyrrolo[1,2-*a*]pyrazine-1,3,6(2*H*,4*H*,7*H*)-trione (**7g**)

Yellow oil (3:1 Hex/EtOAc). 81% yield, 431 mg. [α]_D_ = +150.8° (*c* = 0.38, MeOH). ^1^H NMR (300 MHz, CDCl_3_) δ: 4.55 (d, *J* = 7.6 Hz, 1H), 4.39–4.34 (m, 1H), 3.75–3.70 (m, 2H), 2.63–2.13 (m, 5H), 1.49–1.38 (m, 2H), 1.26 (sext, *J* = 7.9 Hz, 2H), 1.04 (d, *J* = 6.7 Hz, 3H), 0.93 (d, *J* = 6.8 Hz, 3H), 0.87 (t, *J* = 7.3 Hz, 3H). ^13^C{^1^H} NMR (75 MHz, CDCl_3_) δ: 173.8 (C_q_), 170.9 (C_q_), 169.1 (C_q_), 58.8 (CH), 55.5 (CH), 39.8 (CH_2_), 31.2 (CH), 29.9 (CH_2_), 29.4 (CH_2_), 22.5 (CH_2_), 20.0 (CH_2_), 19.4 (CH_3_), 19.3 (CH_3_), 13.6 (CH_3_). HRMS (ESI) *m*/*z*: [M + H]^+^ calcd for C_14_H_23_N_2_O_3_ 267.1703; found 267.1699.

### (4*S*,8a*S*)-4-Benzyl-2-butyldihydropyrrolo[1,2-*a*]pyrazine-1,3,6(2*H*,4*H*,7*H*)-trione (**7h**)

Yellow oil (3:1 Hex/EtOAc). 69% yield, 434 mg. [α]_D_ = −39.1° (*c* = 0.27, MeOH). ^1^H NMR (300 MHz, CDCl_3_) δ: 7.30–7.10 (m, 5H), 5.09 (t, *J* = 5.5 Hz, 1H), 3.82–3.67 (m, 2H), 3.49–3.45 (m, 1H), 3.34 (dd, *J* = 13.9, 5.5 Hz, 1H), 3.23 (dd, *J* = 13.9, 5.5 Hz, 1H), 2.43–2.08 (m, 4H), 1.55–1.39 (m, 2H), 1.28 (sext, *J* = 7.3 Hz, 2H), 0.92 (t, *J* = 7.2 Hz, 3H). ^13^C{^1^H} NMR (75 MHz, CDCl_3_) δ: 173.3 (C_q_), 170.3 (C_q_), 170.0 (C_q_), 135.7 (C_q_), 129.1 (CH_Ar_), 129.0 (CH_Ar_), 127.7 (CH_Ar_), 55.1 (CH), 54.8 (CH), 40.2 (CH_2_), 37.3 (CH_2_), 29.8 (CH_2_), 29.3 (CH_2_), 21.7 (CH_2_), 20.1 (CH_2_), 13.7 (CH_3_). HRMS (ESI) *m*/*z*: [M + H]^+^ calcd for C_18_H_23_N_2_O_3_ 315.1703; found 315.1710.

### General Procedure for the Synthesis of α-Methylidene-γ-lactam-Fused 2,6-Diketopiperazines

The corresponding α-aminoester hydrochloride **1a**–**e** (2 mmol, 1 equiv) was treated with potassium hydroxide (1.8 mmol, 0.9 equiv) in methanol (10 mL) and the resulting suspension sonicated for 10 min. Subsequently, 2-(bromomethyl)acrylic acid (**2b**) or 3-bromo-2-(bromomethyl)propionic acid (**2c**) (2 mmol, 1 equiv) was added to the reaction mixture, followed by phenylglyoxal **3** (2 mmol, 1 equiv) and isocyanide **4a**,**b** (2 mmol, 1 equiv). The reaction mixture was stirred at room temperature for 24 h, and the solvent was removed under reduced pressure. The raw product was dissolved in acetonitrile (10 mL), treated with cesium carbonate (4 mmol), and heated to reflux with a heating block for 1 h. Subsequently, the solvent was removed under reduced pressure and the crude product dissolved in chloroform. This solution was washed with water and the organic layer dried over sodium sulfate, filtered, and concentrated to dryness. The residue was purified by column chromatography, employing SiO_2_ as stationary phase and a hexane/ethyl acetate mixture as eluent.

### 2-Cyclohexyl-7-methylenedihydropyrrolo[1,2-*a*]pyrazine-1,3,6(2*H*,4*H*,7*H*)-trione (**8a**)

Orange oil (3:1 Hex/EtOAc). 85% yield, 446 mg. ^1^H NMR (300 MHz, CDCl_3_) δ: 6.11 (t, *J* = 2.7 Hz, 1H), 5.50 (t, *J* = 2.6 Hz, 1H), 4.96 (d, *J* = 18.7 Hz, 1H), 4.49 (tt, *J* = 11.8, 3.7 Hz, 1H), 4.28 (t, *J* = 6.7 Hz, 1H), 3.90 (d, *J* = 18.8 Hz, 1H), 3.19 (dt, *J* = 5.4, 2.7 Hz, 2H), 2.26–0.82 (m, 10H). ^13^C{^1^H} NMR (75 MHz, CDCl_3_) δ: 170.4 (C_q_), 167.7 (C_q_), 166.1 (C_q_), 136.2 (C_q_), 118.6 (CH_2_), 54.5 (CH), 54.3 (CH), 44.6 (CH_2_), 29.3 (CH_2_), 28.7 (CH_2_), 27.7 (CH_2_), 26.4 (CH_2_), 26.3 (CH_2_), 25.2 (CH_2_). HRMS (ESI) *m*/*z*: [M + H]^+^ calcd for C_14_H_19_N_2_O_3_ 263.1390, found 263.1395.

### (4*S*,8a*S*)-2-Cyclohexyl-7-methylene-4-phenyldihydropyrrolo[1,2-*a*]pyrazine-1,3,6(2*H*,4*H*,7*H*)-trione (**8b**)

Yellow oil (3:1 Hex/EtOAc). 77% yield, 521 mg. [α]_D_ = −36.0 (*c* = 0.11, acetone). ^1^H NMR (300 MHz, CDCl_3_) δ: 7.42–7.27 (m, 5H), 6.16 (t, *J* = 2.8 Hz, 1H), 6.13 (s, 1H), 5.53 (t, *J* = 2.4 Hz, 1H), 4.62 (tt, *J* = 12.2, 3.7 Hz, 1H), 4.19 (dd, *J* = 8.8, 5.2 Hz, 1H), 3.25–3.06 (m, 2H), 1.88–1.18 (m, 10 H). ^13^C{^1^H} NMR (75 MHz, CDCl_3_) δ: 170.9 (C_q_), 168.3 (C_q_), 166.2 (C_q_), 136.4 (C_q_), 133.6 (C_q_), 129.5 (CH_Ar_), 129.1 (CH_Ar_), 127.1 (CH_Ar_), 118.8 (CH_2_), 57.2 (CH), 54.4 (CH), 51.9 (CH), 29.2 (CH_2_), 29.1 (CH_2_), 28.1 (CH_2_), 26.4 (CH_2_), 25.3 (CH_2_). HRMS (ESI) *m*/*z*: [M + H]^+^ calcd for C_20_H_23_N_2_O_3_ 339.1703; found 339.1709.

### (4*S*,8a*S*)-2-Cyclohexyl-4-isopropyl-7-methylenedihydropyrrolo[1,2-*a*]pyrazine-1,3,6(2*H*,4*H*,7*H*)-trione (**8c**)

Yellow oil (3:1 Hex/EtOAc). 83% yield, 505 mg. [α]_D_ = −3.5 (*c* = 0.90, acetone). ^1^H NMR (300 MHz, CDCl_3_) δ: 6.08 (t, *J* = 2.9 Hz, 1H), 5.48 (t, *J* = 2.5 Hz, 1H), 4.63 (d, *J* = 7.4 Hz, 1H), 4.50 (tt, *J* = 12.2, 3.7 Hz, 1H), 4.37 (dd, *J* = 9.1, 4.7 Hz, 1H), 3.24 (ddt, *J* = 17.9, 9.1, 2.5 Hz, 1H), 3.05 (ddt, *J* = 17.7, 4.9, 2.8 Hz, 1H), 2.37–2.24 (m, 1H), 2.24–1.12 (m, 10H), 1.09 (d, *J* = 6.8 Hz, 3H), 0.97 (d, *J* = 6.9 Hz, 3H). ^13^C{^1^H} NMR (75 MHz, CDCl_3_) δ: 170.8 (C_q_), 169.5 (C_q_), 167.0 (C_q_), 136.2 (C_q_), 118.1 (CH_2_), 59.7 (CH), 54.0 (CH), 53.4 (CH), 31.4 (CH), 29.0 (CH_2_), 28.9 (CH_2_), 28.5 (CH_2_), 26.3 (CH_2_), 25.2 (CH_2_), 19.5 (CH_3_), 19.4 (CH_3_). HRMS (ESI) *m*/*z*: [M + H]^+^ calcd for C_17_H_25_N_2_O_3_ 305.1860; found 305.1862.

### (4*S*,8a*S*)-2-Cyclohexyl-4-methyl-7-methylenedihydropyrrolo[1,2-*a*]pyrazine-1,3,6(2*H*,4*H*,7*H*)-trione (**8d**)

Yellow oil (3:1 Hex/EtOAc). 61% yield, 337 mg. [α]_D_ = −23.1 (*c* = 0.13, acetone). ^1^H NMR (300 MHz, CDCl_3_) δ: 6.07 (t, *J* = 2.8 Hz, 1H), 5.47 (t, *J* = 2.4 Hz, 1H), 4.95 (q, *J* = 7.2 Hz, 1H), 4.45 (tt, *J* = 12.4, 3.9 Hz, 1H), 4.32 (dd, *J* = 7.7, 5.7 Hz, 1H), 3.20–3.10 (m, 2H), 2.23–1.09 (m, 10H), 1.50 (d, *J* = 7.2 Hz, 3H). ^13^C{^1^H} NMR (75 MHz, CDCl_3_) δ: 171.2 (C_q_), 170.5 (C_q_), 165.8 (C_q_), 136.5 (C_q_), 118.2 (CH_2_), 54.2 (CH), 51.5 (CH), 50.1 (CH), 29.2 (CH_2_), 28.7 (CH_2_), 27.6 (CH_2_), 26.4 (CH_2_), 26.3 (CH_2_), 25.2 (CH_2_), 16.1 (CH_3_). HRMS (ESI) *m*/*z*: [M + H]^+^ calcd for C_15_H_21_N_2_O_3_ 277.1547; found 277.1553.

### (4*S*,8a*S*)-4-Benzyl-2-cyclohexyl-7-methylenedihydropyrrolo[1,2-*a*]pyrazine-1,3,6(2*H*,4*H*,7*H*)-trione (**8e**)

Light yellow oil (3:1 Hex/EtOAc). 71% yield, 500 mg. [α]_D_ = −38.7 (*c* = 0.15, acetone). ^1^H NMR (300 MHz, CDCl_3_) δ: 7.27–7.12 (m, 5H), 6.07 (t, *J* = 2.8 Hz, 1H), 5.43 (t, *J* = 2.4 Hz, 1H), 5.14 (t, *J* = 5.2 Hz, 1H), 4.49 (tt, *J* = 12.5, 3.8 Hz, 1H), 3.47 (dd, *J* = 13.9, 5.0 Hz, 1H), 3.32 (dd, *J* = 8.4, 5.2 Hz, 1H), 3.23 (dd, *J* = 13.9, 5.4 Hz, 1H), 3.02–2.84 (m, 2H), 2.30–0.82 (m, 10H). ^13^C{^1^H} NMR (75 MHz, CDCl_3_) δ: 170.4 (C_q_), 170.3 (C_q_), 136.2 (C_q_), 136.0 (C_q_), 129.4 (CH_Ar_), 129.1 (CH_Ar_), 127.8 (CH_Ar_), 118.2 (CH_2_), 55.7 (CH), 54.4 (CH), 53.1 (CH), 37.6 (CH_2_), 29.3 (CH_2_), 28.6 (CH_2_), 27.7 (CH_2_), 26.4 (CH_2_), 26.3 (CH_2_), 25.3 (CH_2_). HRMS (ESI) *m*/*z*: [M + H]^+^ calcd for C_21_H_25_N_2_O_3_ 353.1860; found 353.1869.

## Supporting Information Available

Copies of ^1^H, ^13^C, and DEPT-135 NMR and ESI-HRMS spectra. X-ray crystal data and refinement details for **7c**. Olex2 plot of **7c**. Gibbs free energies in Hartree of epimers of **7c** on C8a (PDF)
