# Supplementary material for: One-Pot Diastereoselective Synthesis of Pyrrolopiperazine-2,6-diones by a Ugi/Nucleophilic Substitution/N-Acylation Sequence
Source: J Org Chem. 2022 Jun 27;87(14):9391–8. doi: 10.1021/acs.joc.2c00694 (PMC9348836; doi:10.1021/acs.joc.2c00694)
Supplement: Supplementary file 1 — jo2c00694_si_001.pdf [file jo2c00694_si_001.pdf]

The Journal of Organic Chemistry

Supporting Information

One-pot diastereoselective synthesis of pyrrolopiperazine-2,6-diones by an Ugi/nucleophilic substitution/*N*-acylation sequence

Beatriz González-Saiz, Israel Carreira-Barral, Pablo Pertejo, Javier Gómez-Ayuso, Roberto Quesada, María García-Valverde*

magaval@ubu.es

CONTENTS

NMR and HRMS spectra of the compounds S3

X-Ray diffraction studies S39

Computational study S41

NMR and HRMS spectra of the compounds

(*E*)-Methyl 2-(3-bromo-*N*-(3-(cyclohexylamino)-1-hydroxy-3-oxo-1-phenylprop-1-en-2-yl)propanamido) acetate, 5a

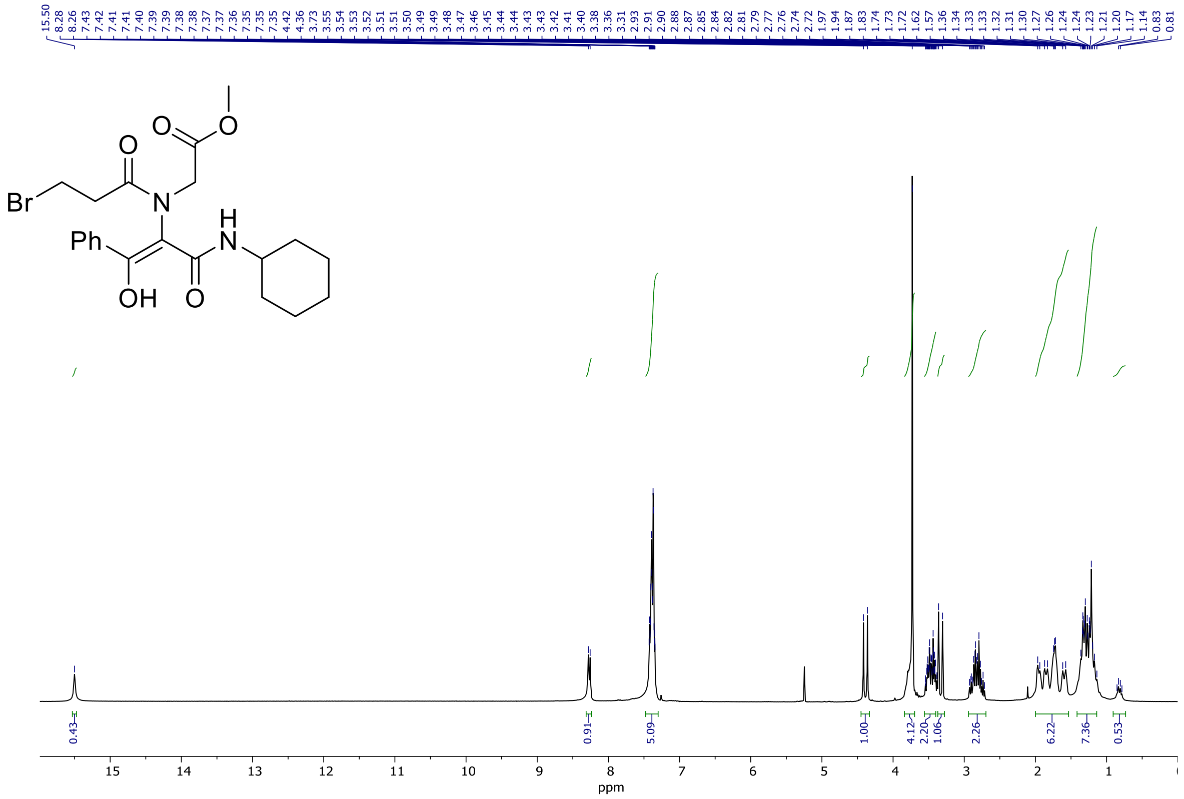

Figure S1. ^{1}H NMR spectrum of **5a** (300 MHz, $CDCl_3$).

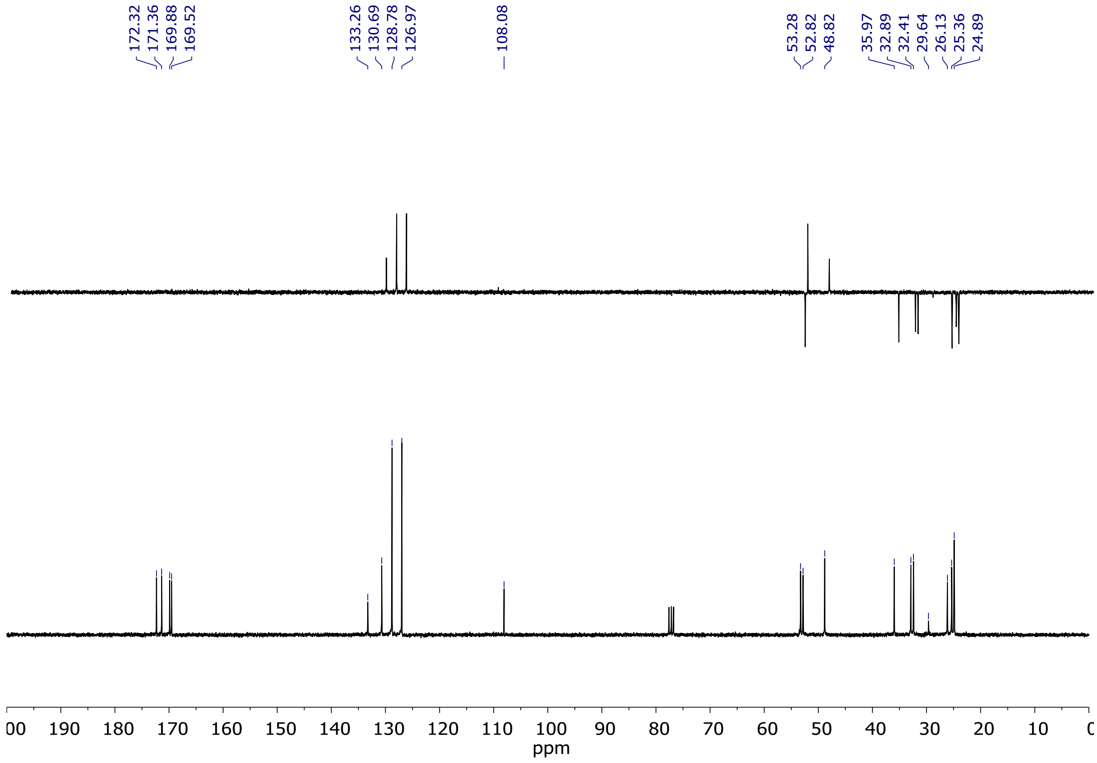

Figure S2. ^{13}C and DEPT NMR spectra of **5a** (75 MHz, $CDCl_3$).

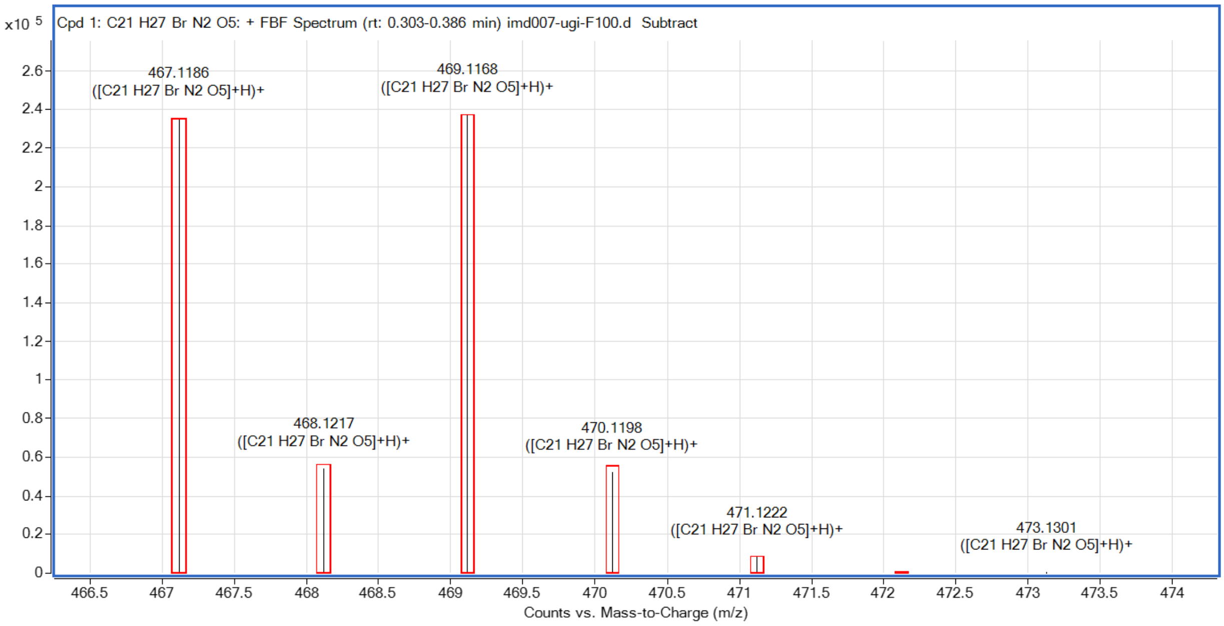

Figure S3. HRMS spectrum of **5a**.

Methyl 2-(2-benzoyl-2-(cyclohexylcarbamoyl)-5-oxopyrrolidin-1-yl)acetate, 6a

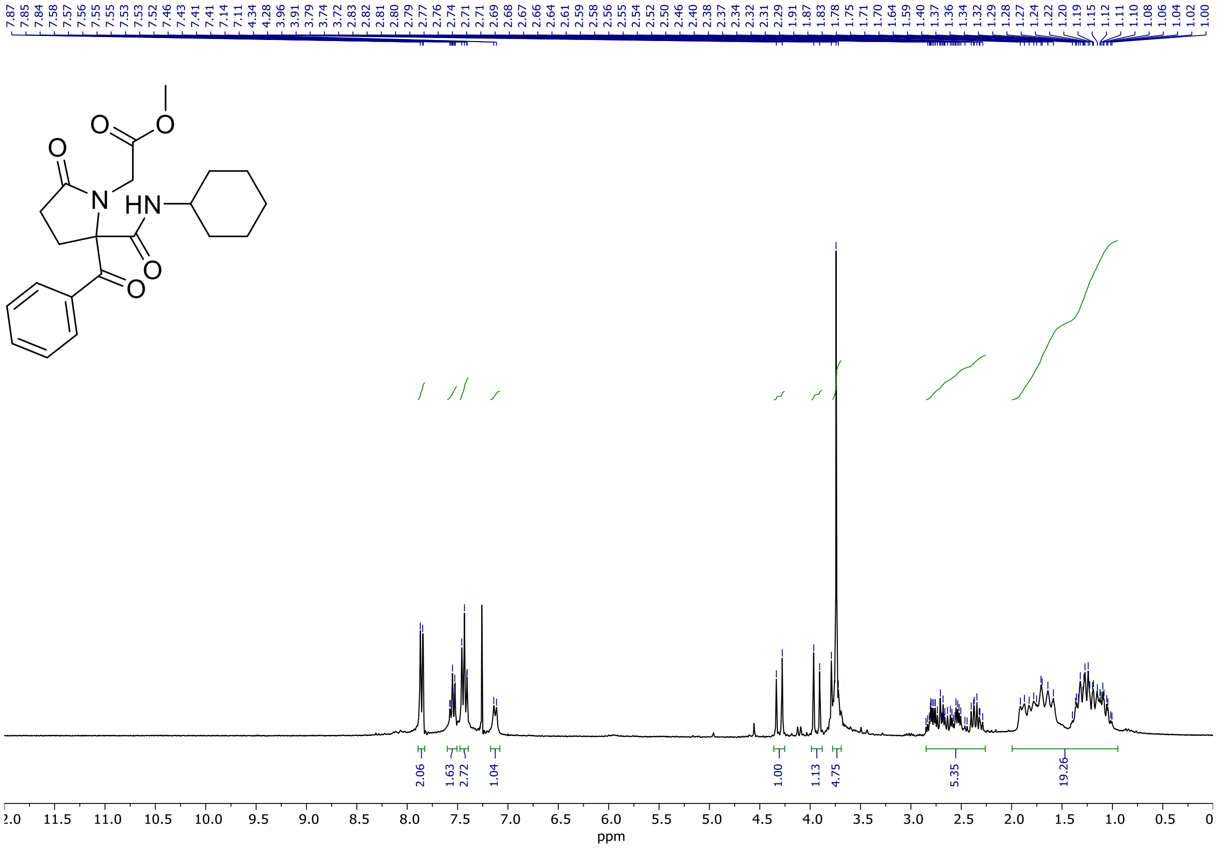

Figure S4. ^{1}H NMR spectrum of **6a** (300 MHz, $CDCl_3$).

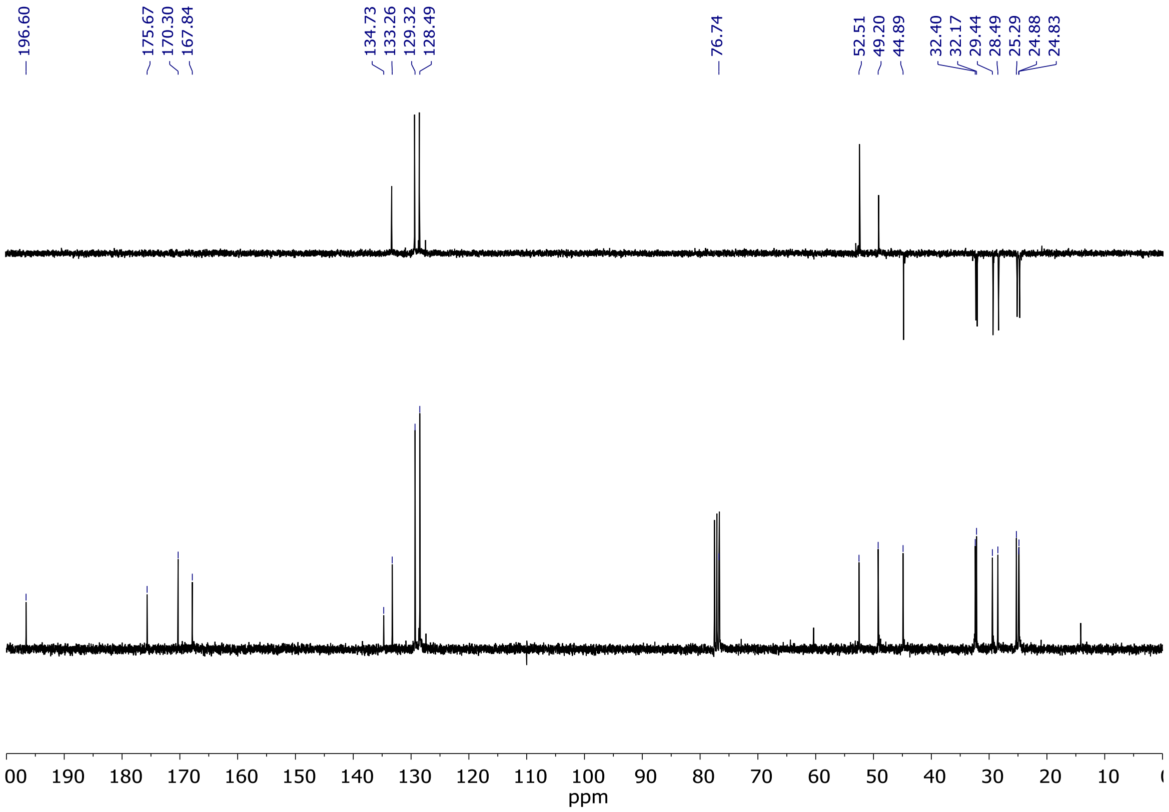

Figure S5. ^{13}C and DEPT NMR spectra of **6a** (75 MHz, $CDCl_3$).

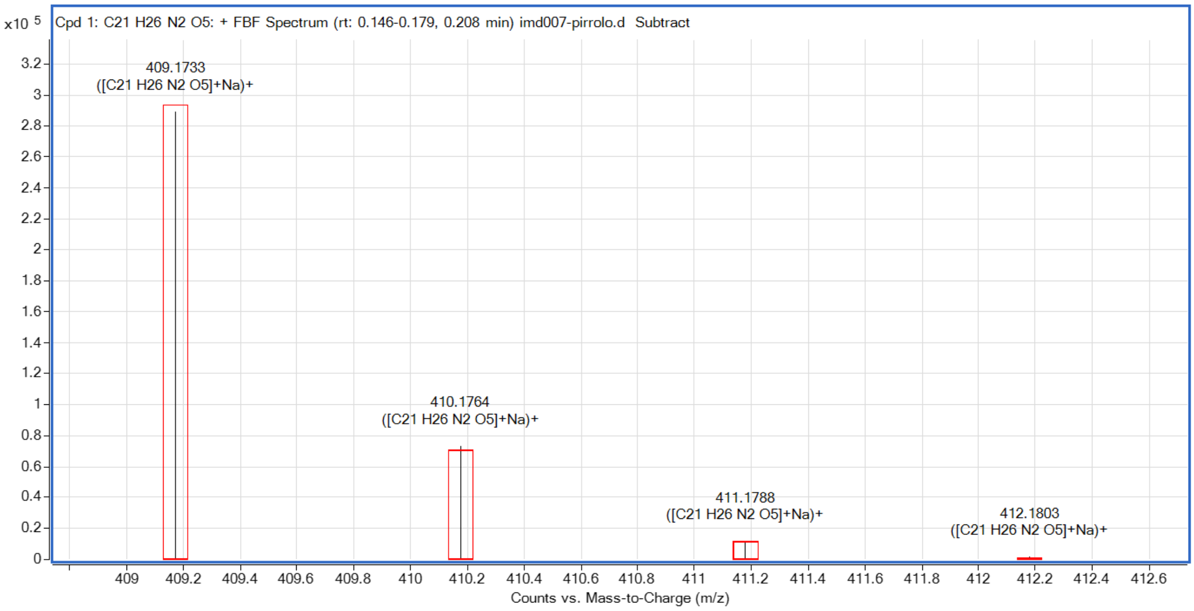

Figure S6. HRMS spectrum of **6a**.

(2*S*)-Methyl 2-(2-benzoyl-2-(cyclohexylcarbamoyl)-5-oxopyrrolidin-1-yl)-2-phenylacetate, 6b

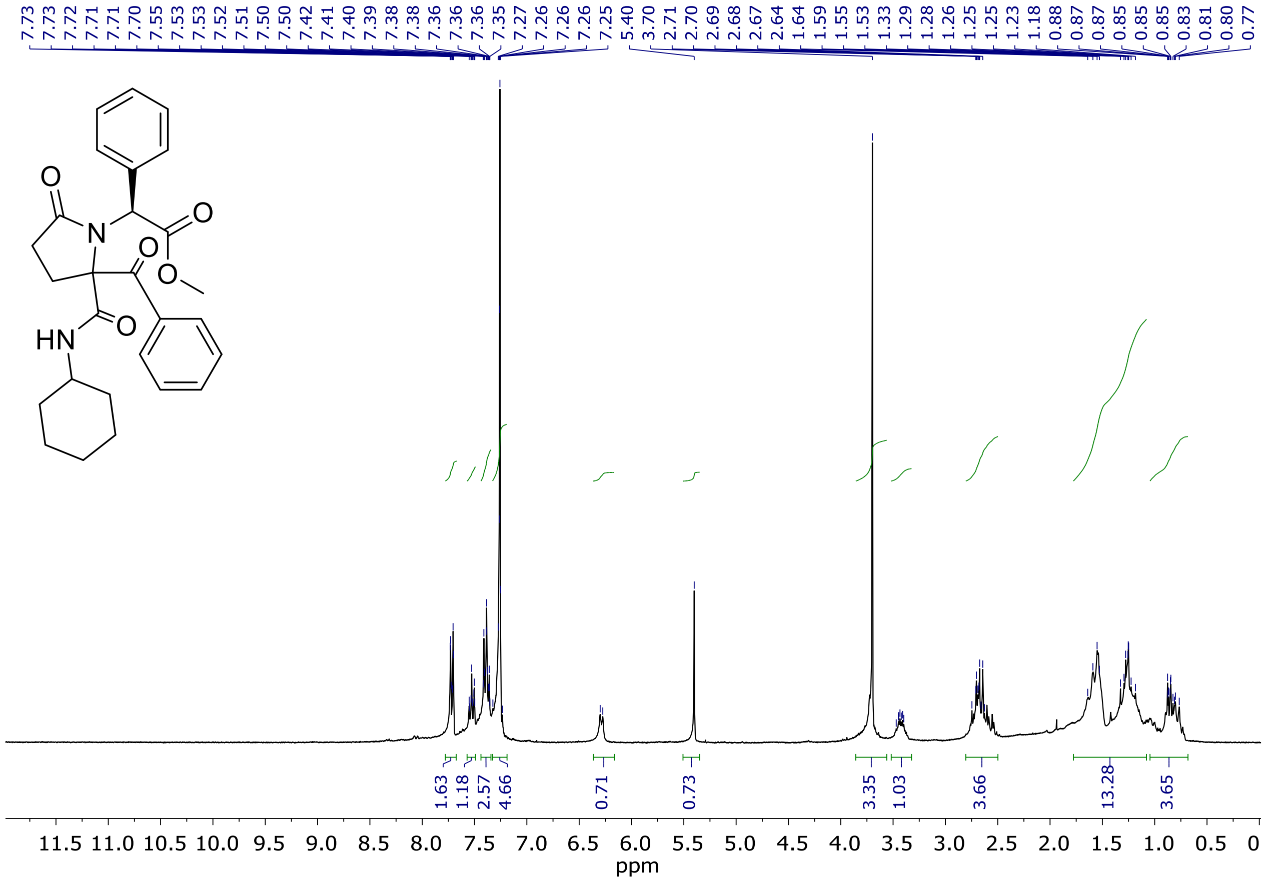

Figure S7. ^{1}H NMR spectrum of **6b** (300 MHz, $CDCl_3$).

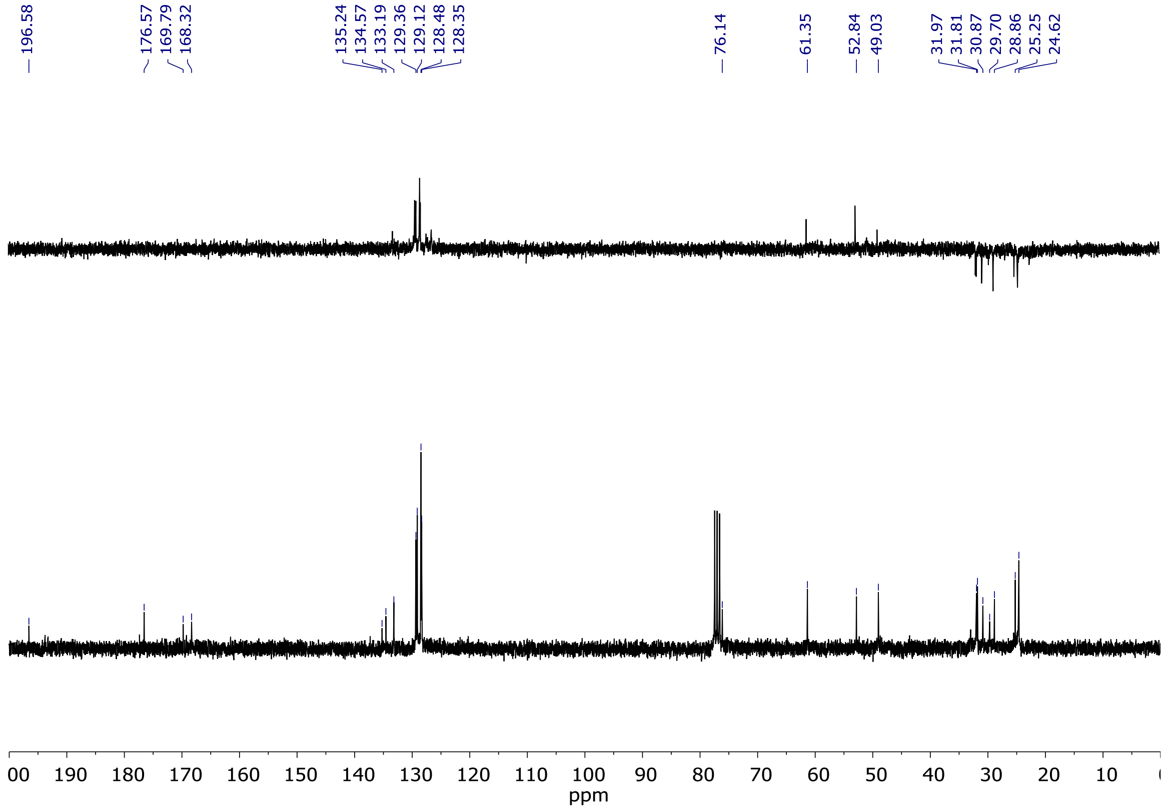

Figure S8. ^{13}C and DEPT NMR spectra of **6b** (75 MHz, $CDCl_3$).

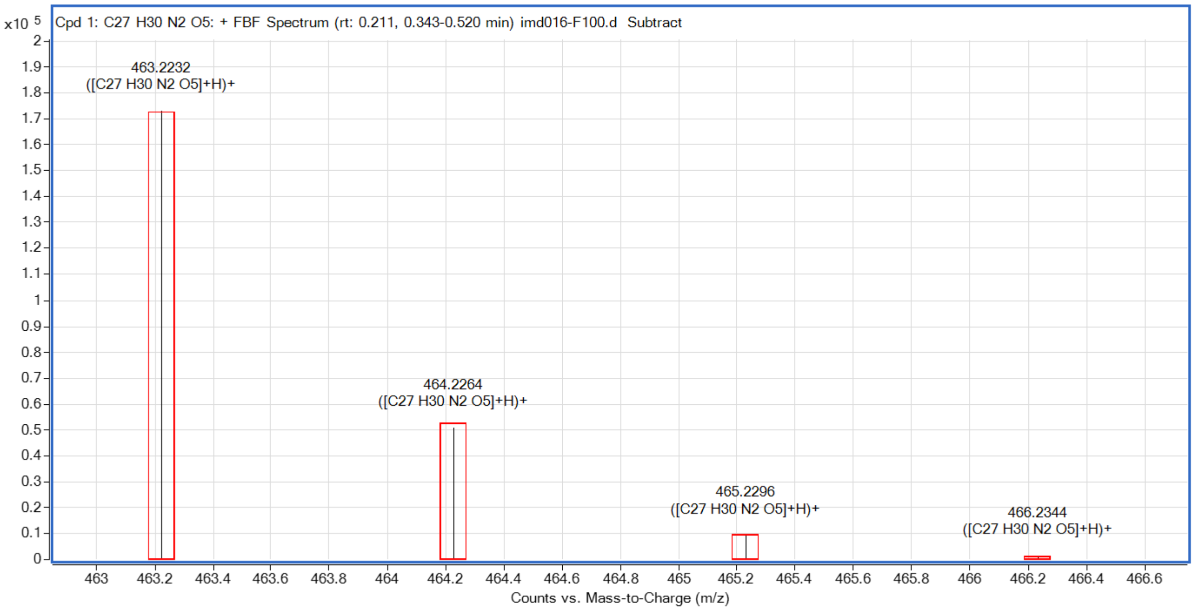

Figure S9. HRMS spectrum of **6b**.

(2*S*)-Methyl 2-(2-benzoyl-2-(cyclohexylcarbamoyl)-5-oxopyrrolidin-1-yl)-3-methylbutanoate, 6c

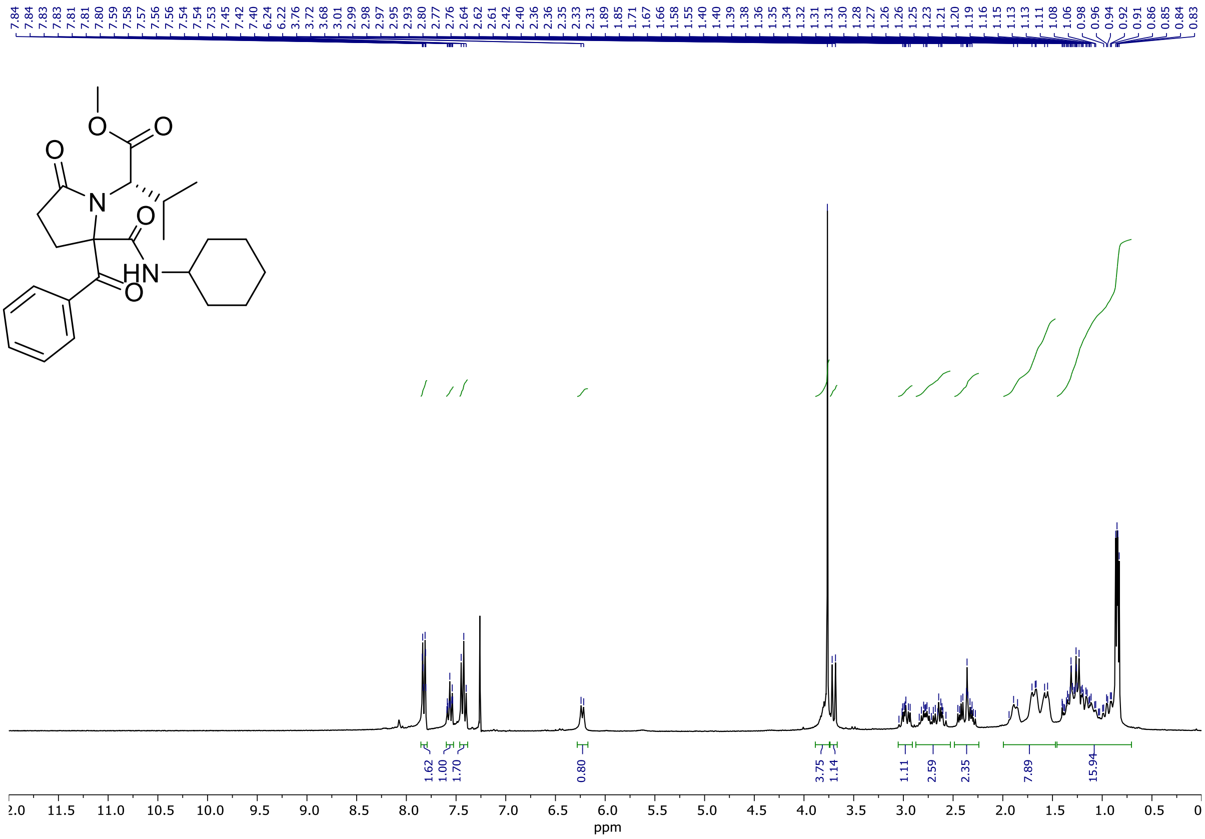

Figure S10. ^{1}H NMR spectrum of **6c** (300 MHz, $CDCl_3$).

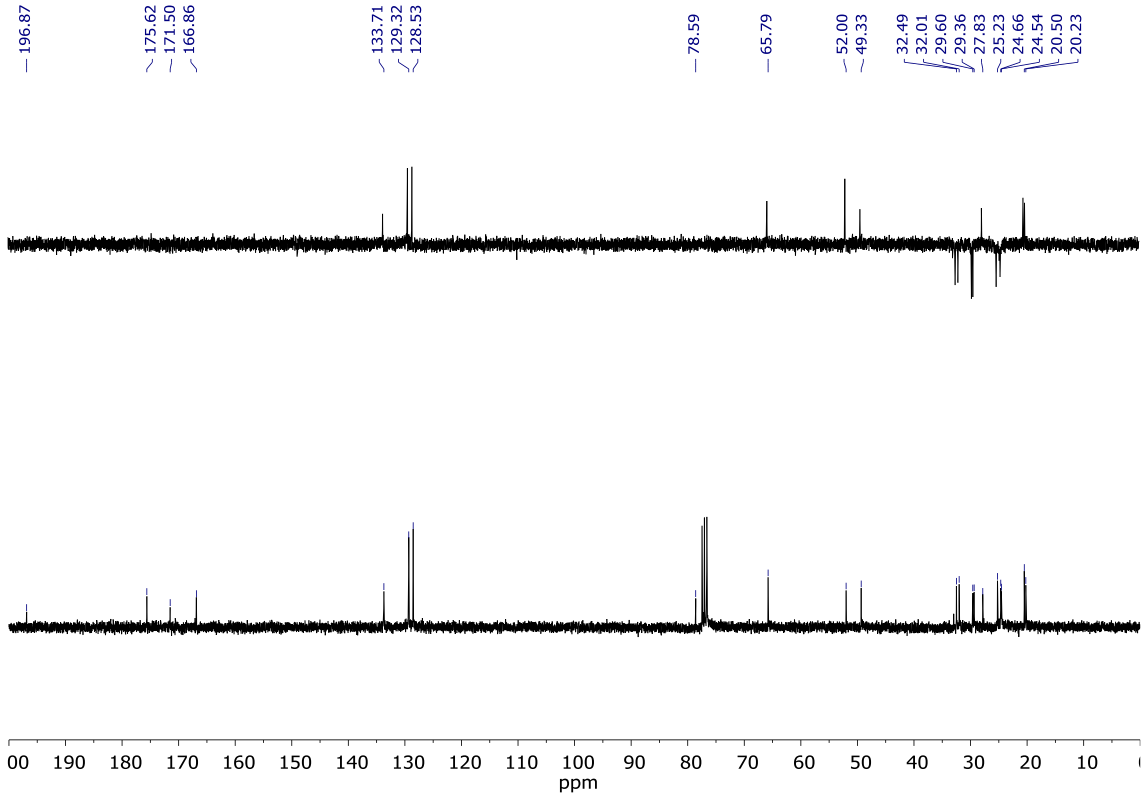

Figure S11. ^{13}C and DEPT NMR spectra of **6c** (75 MHz, $CDCl_3$).

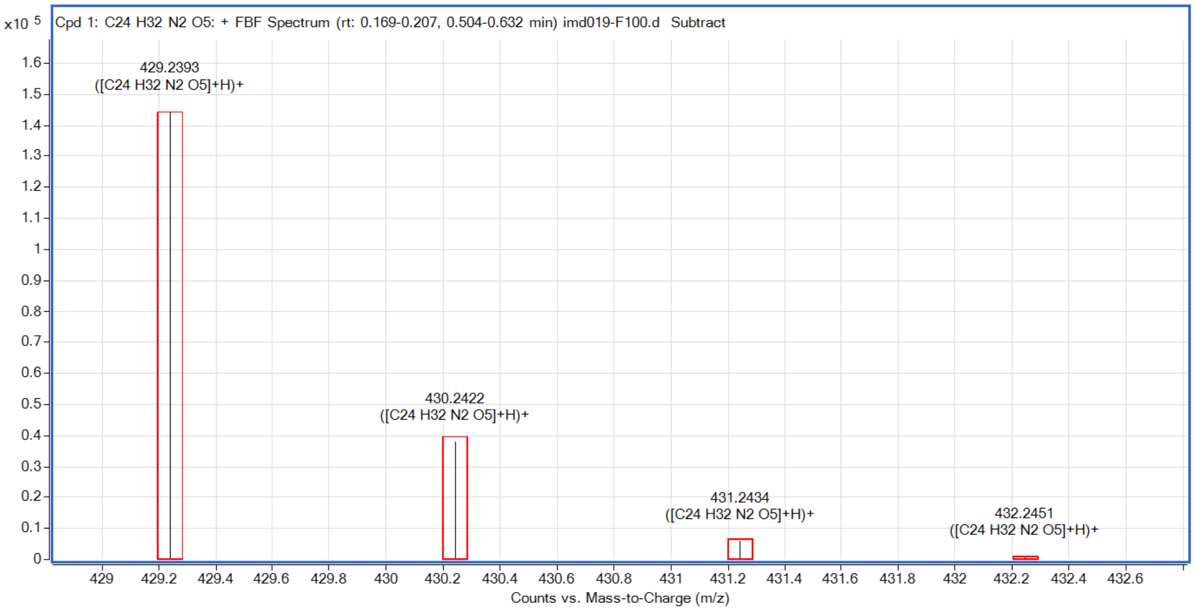

Figure S12. HRMS spectrum of **6c**.

2-Cyclohexyldihydropyrrolo[1,2-*a*]pyrazine-1,3,6(2*H*,4*H*,7*H*)-trione, 7a

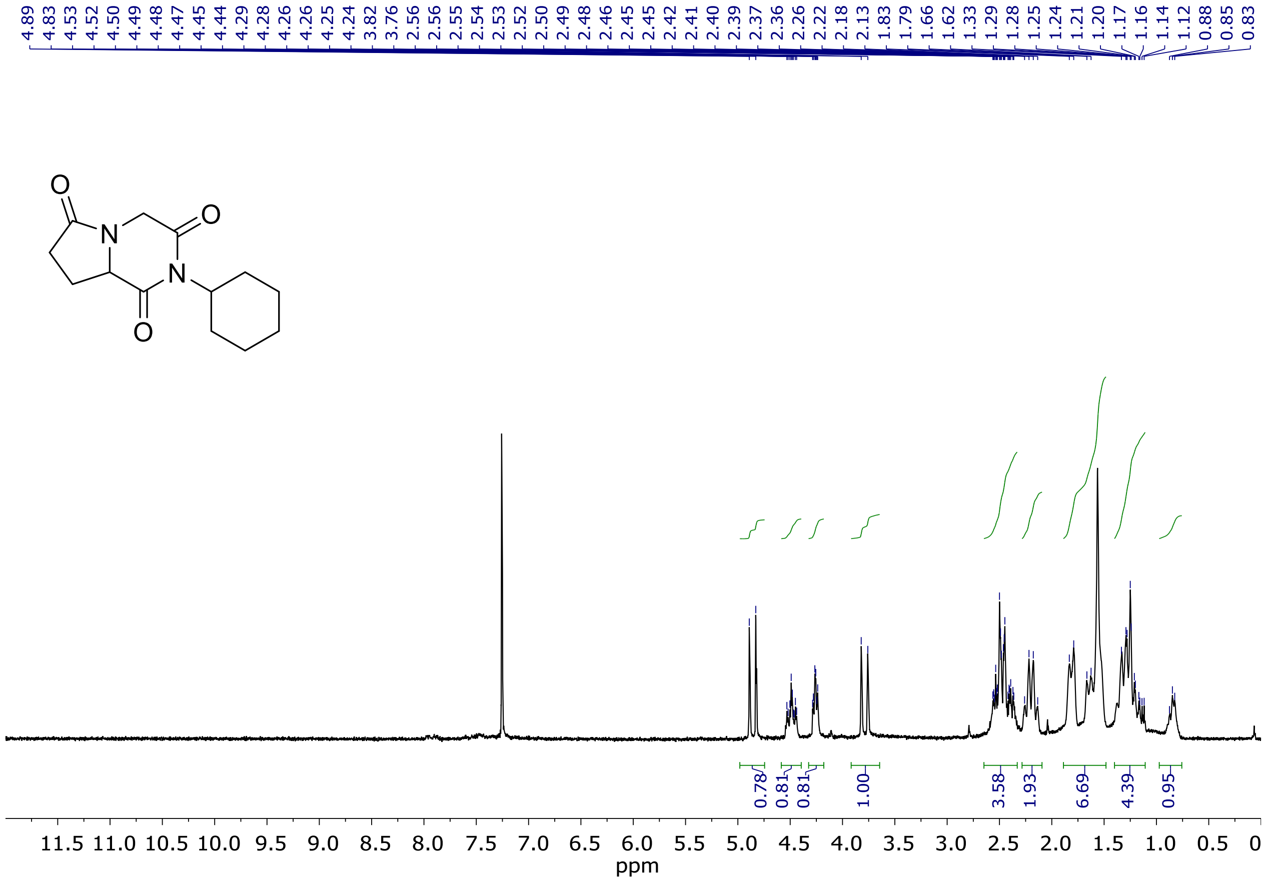

Figure S13. ^{1}H NMR spectrum of **7a** (300 MHz, $CDCl_3$).

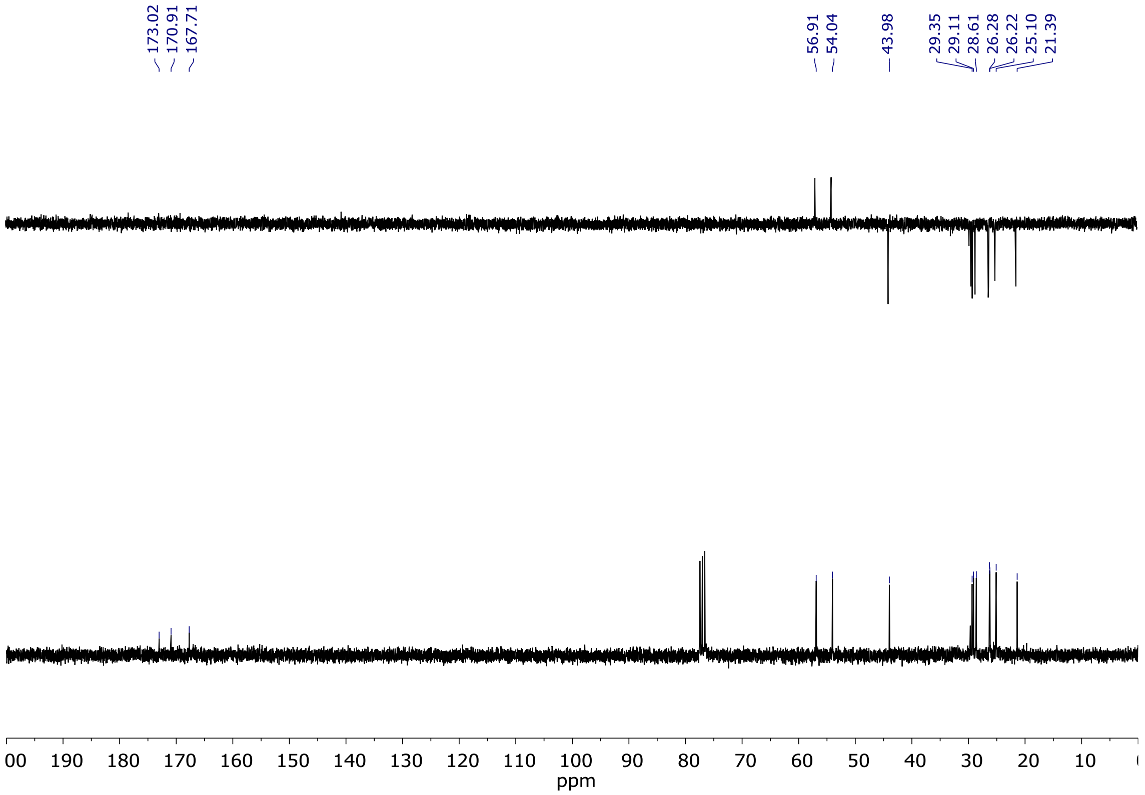

Figure S14. ^{13}C and DEPT NMR spectra of **7a** (75 MHz, $CDCl_3$).

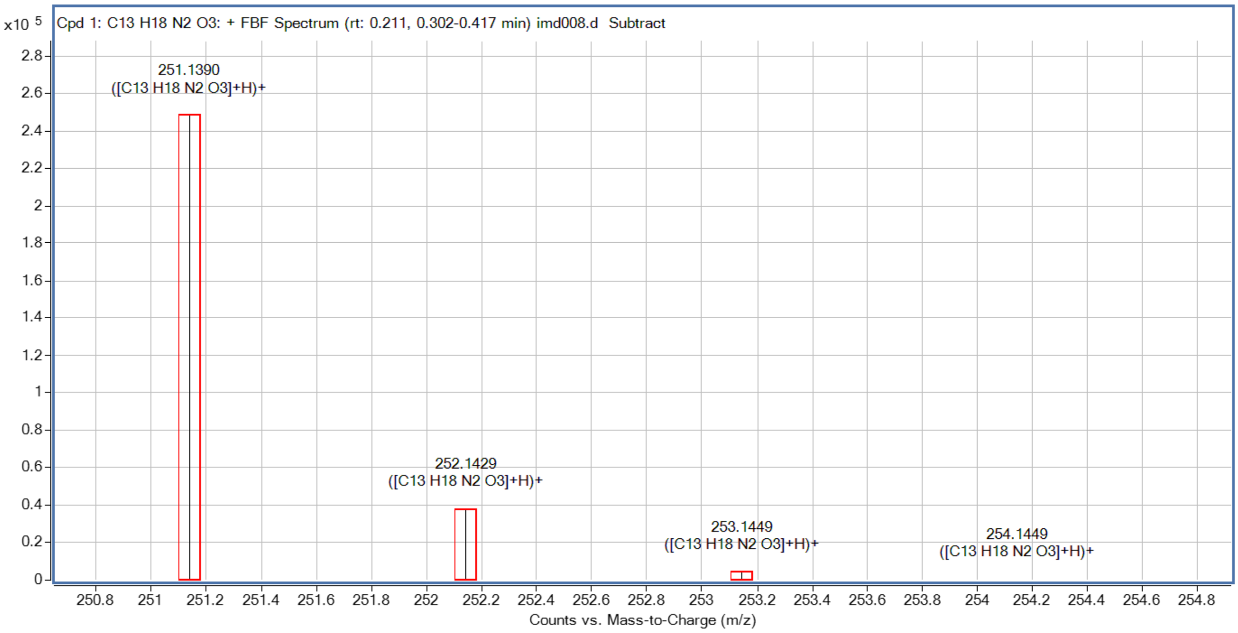

Figure S15. HRMS spectrum of **7a**.

(4*S*,8a*S*)-2-Cyclohexyl-4-phenyldihydropyrrolo[1,2-*a*]pyrazine-1,3,6(2*H*,4*H*,7*H*)-trione, 7b

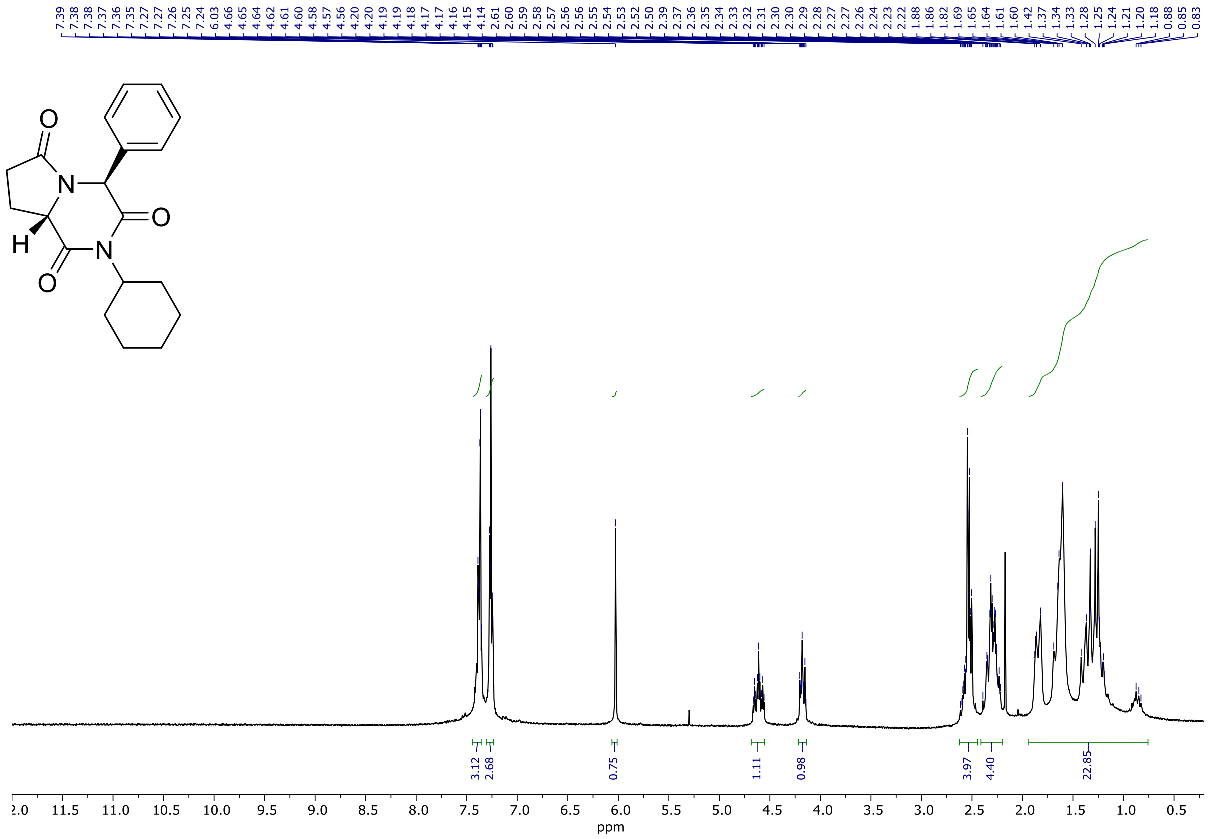

Figure S16. ^{1}H NMR spectrum of **7b** (300 MHz, $CDCl_3$).

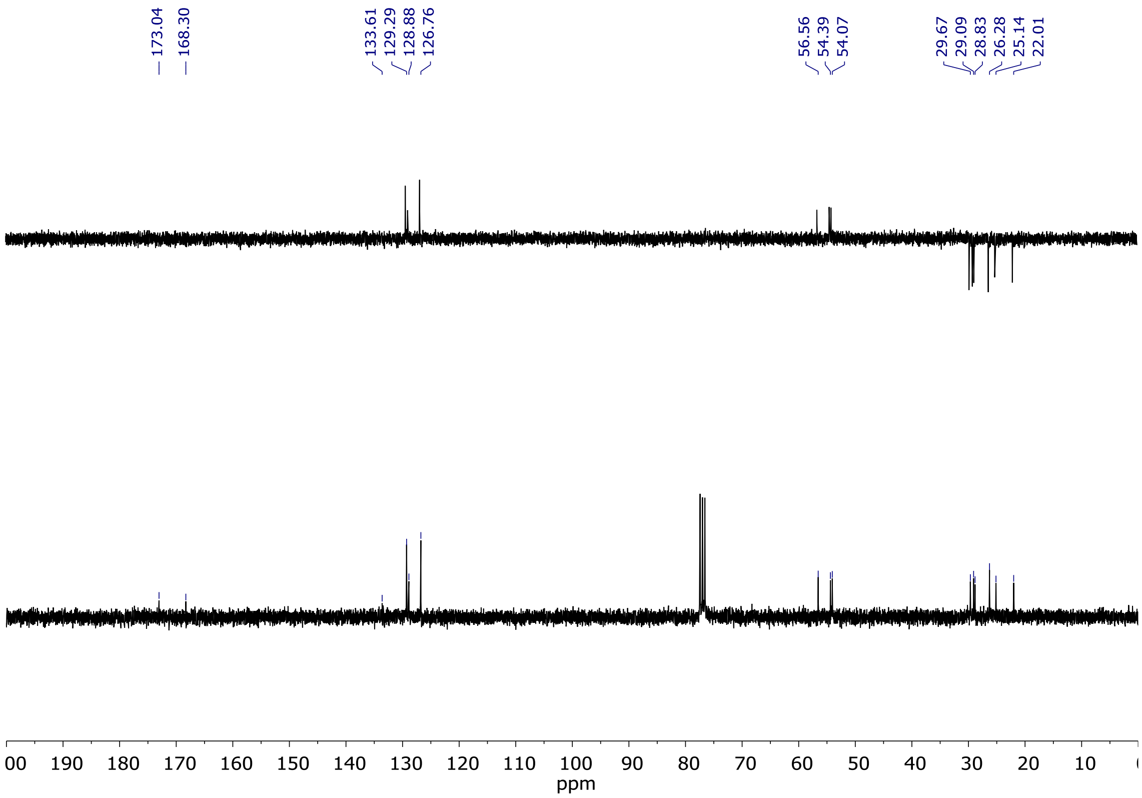

Figure S17. ^{13}C and DEPT NMR spectra of **7b** (75 MHz, $CDCl_3$).

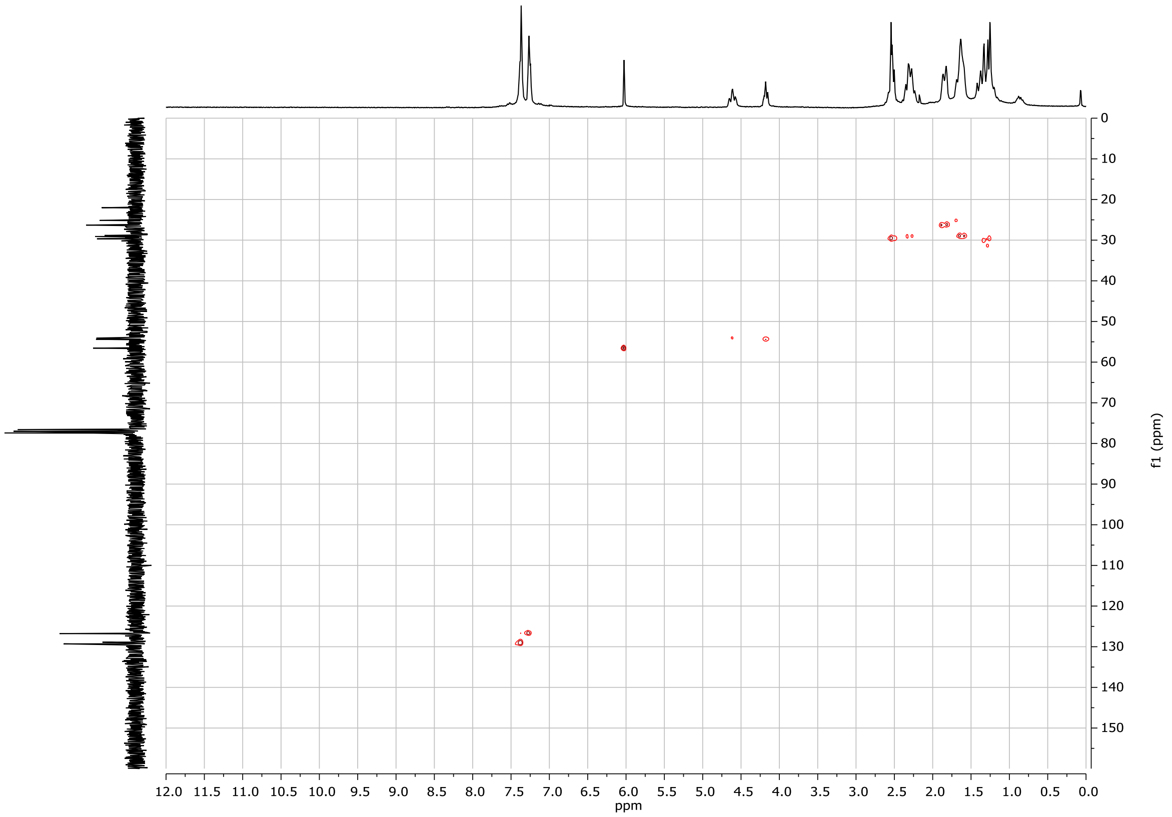

Figure S18. HMQC spectrum of **7b** ($CDCl_3$).

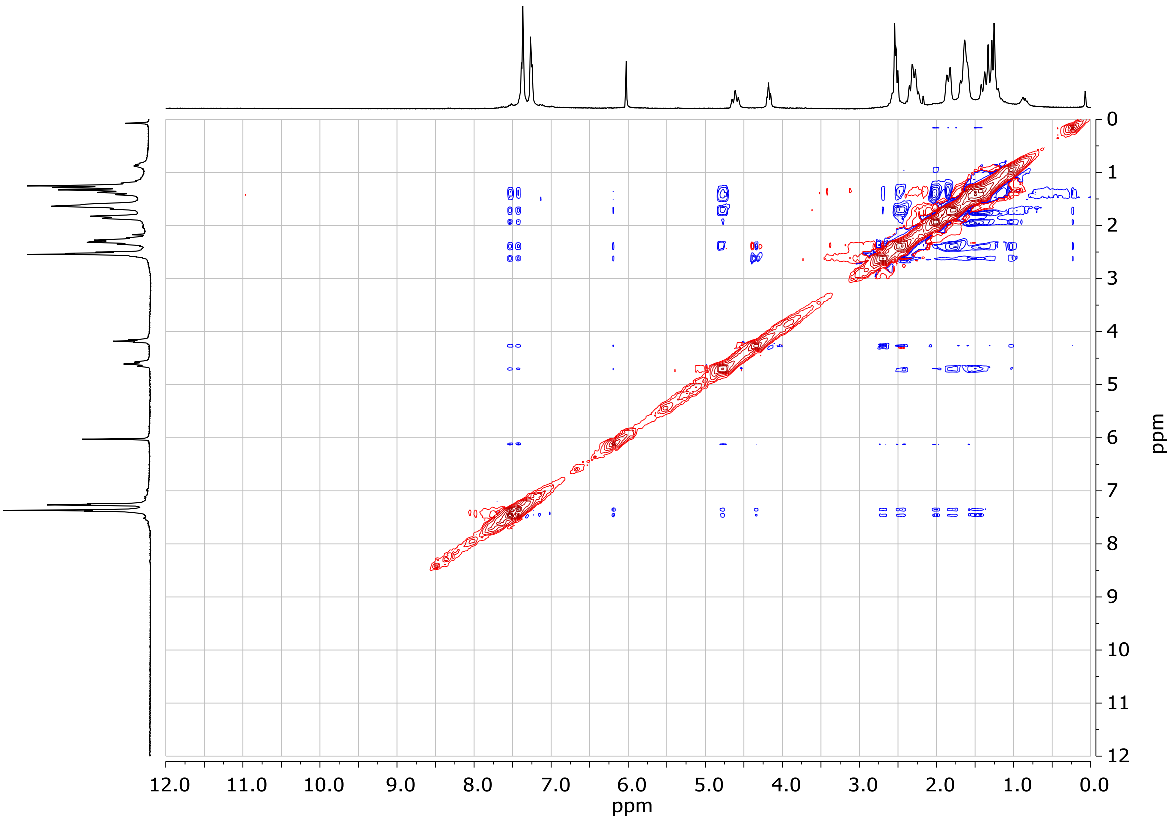

Figure S19. NOESY spectrum of **7b** ($CDCl_3$).

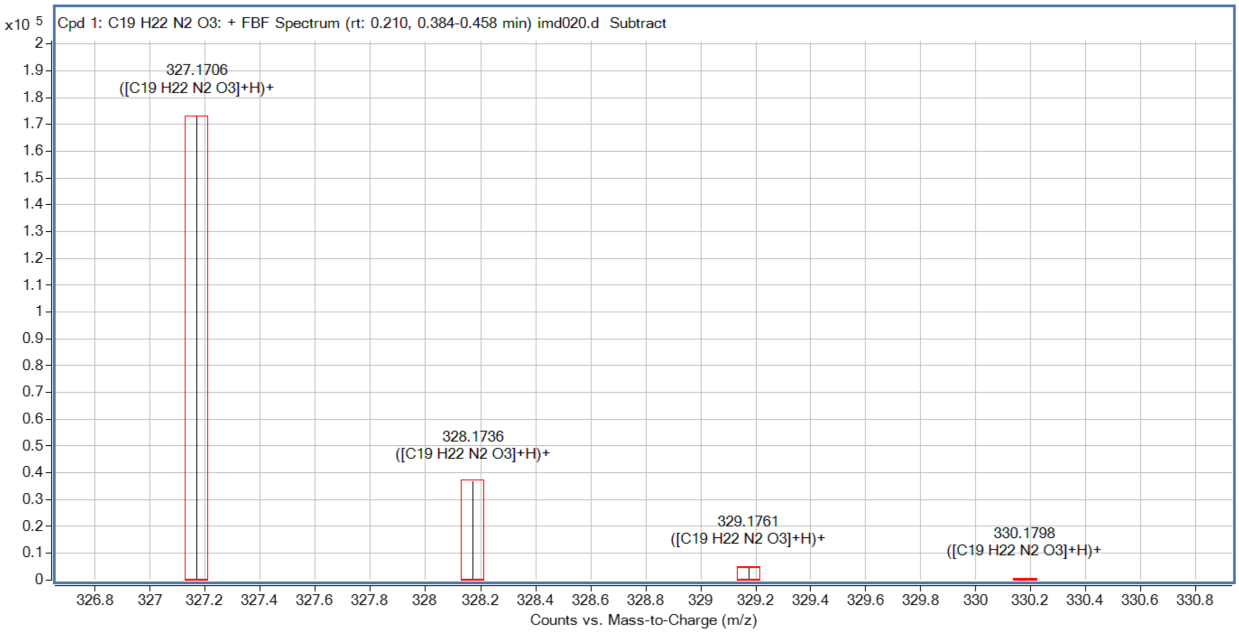

Figure S14. HRMS spectrum of **7b**.

(4*S*,8a*S*)-2-Cyclohexyl-4-isopropyldihydropyrrolo[1,2-*a*]pyrazine-1,3,6(2*H*,4*H*,7*H*)-trione, 7c

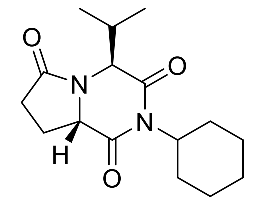

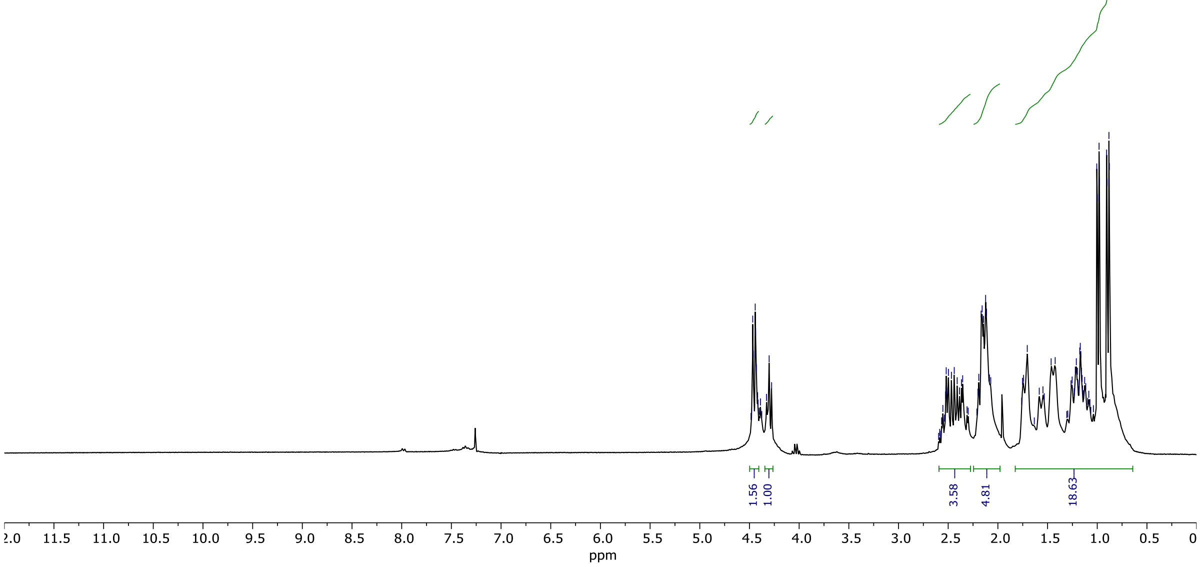

Figure S21. ^{1}H NMR spectrum of **7c** (300 MHz, $CDCl_3$).

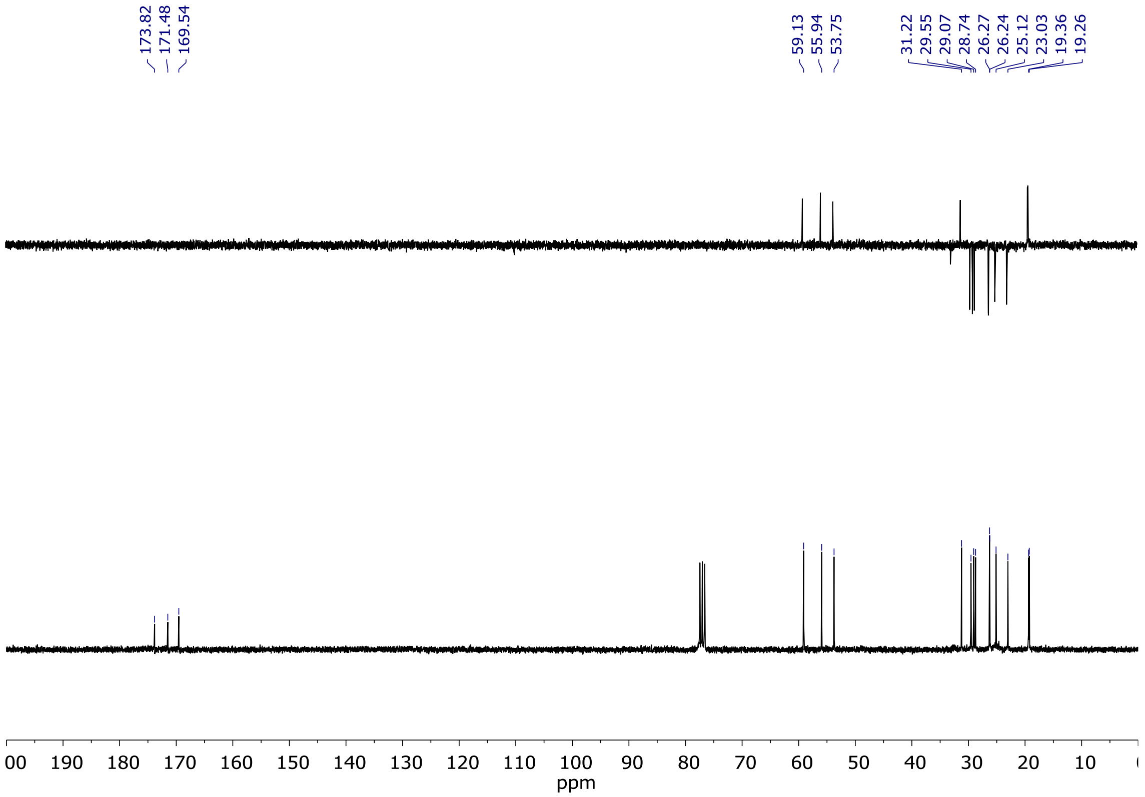

Figure S22. ^{13}C and DEPT NMR spectra of **7c** (75 MHz, $CDCl_3$).

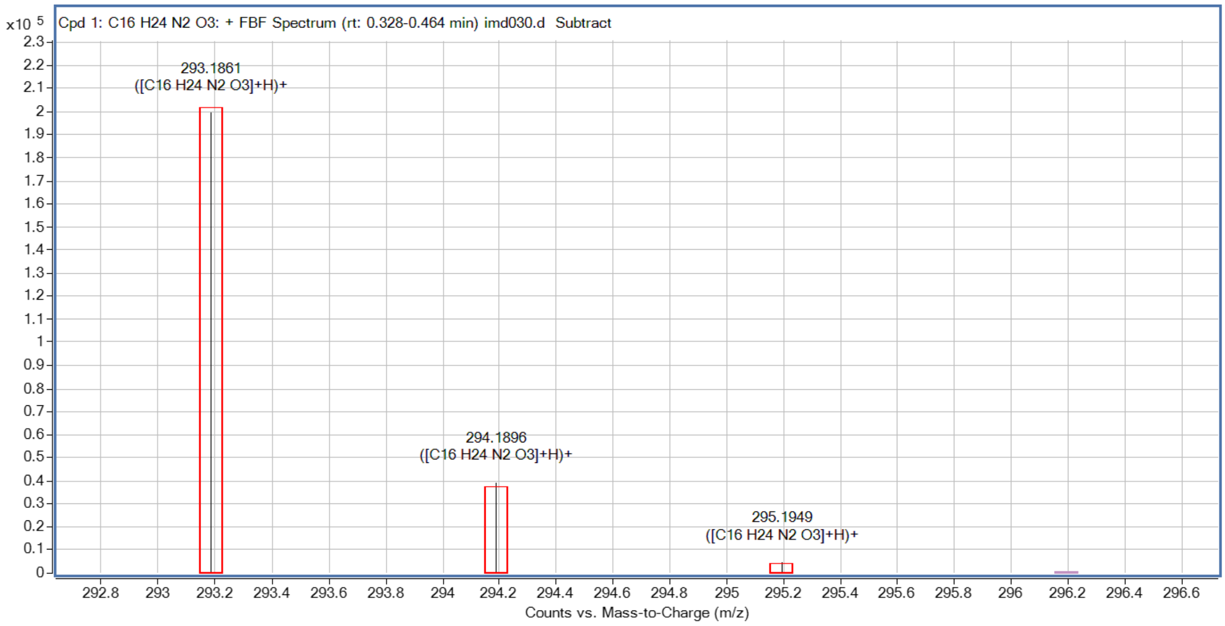

Figure S23. HRMS spectrum of **7c**.

(4*S*,8a*S*)-2-Cyclohexyl-4-methyldihydropyrrolo[1,2-*a*]pyrazine-1,3,6(2*H*,4*H*,7*H*)-trione, 7d

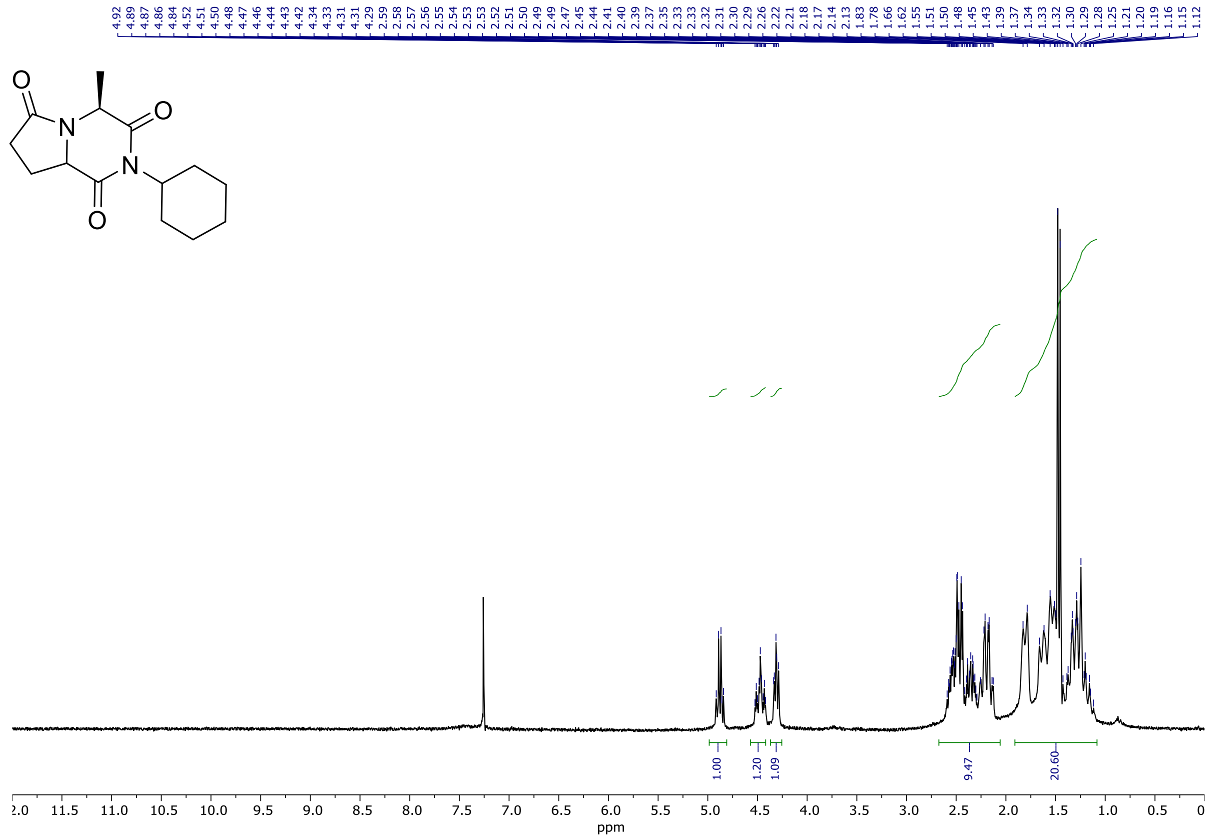

Figure S15. ^{1}H NMR spectrum of **7d** (300 MHz, $CDCl_3$).

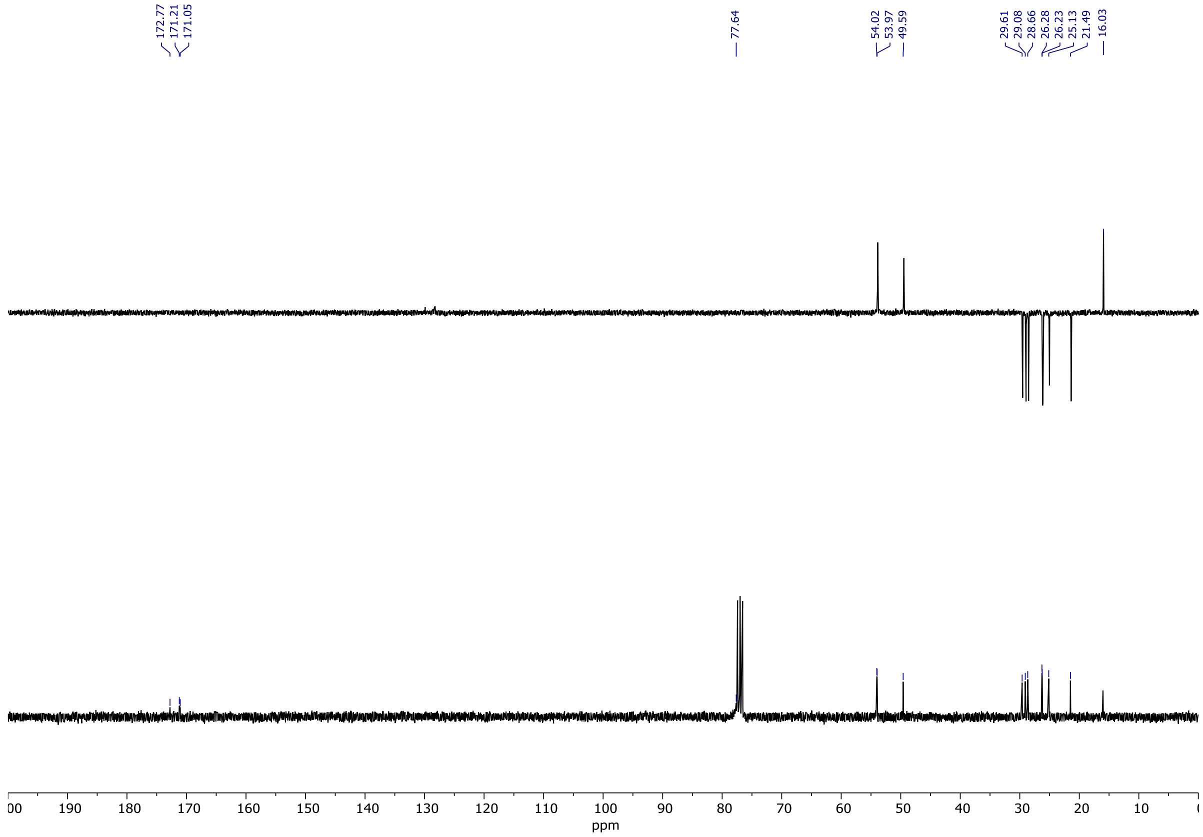

Figure S16. ^{13}C and DEPT NMR spectra of **7d** (75 MHz, $CDCl_3$).

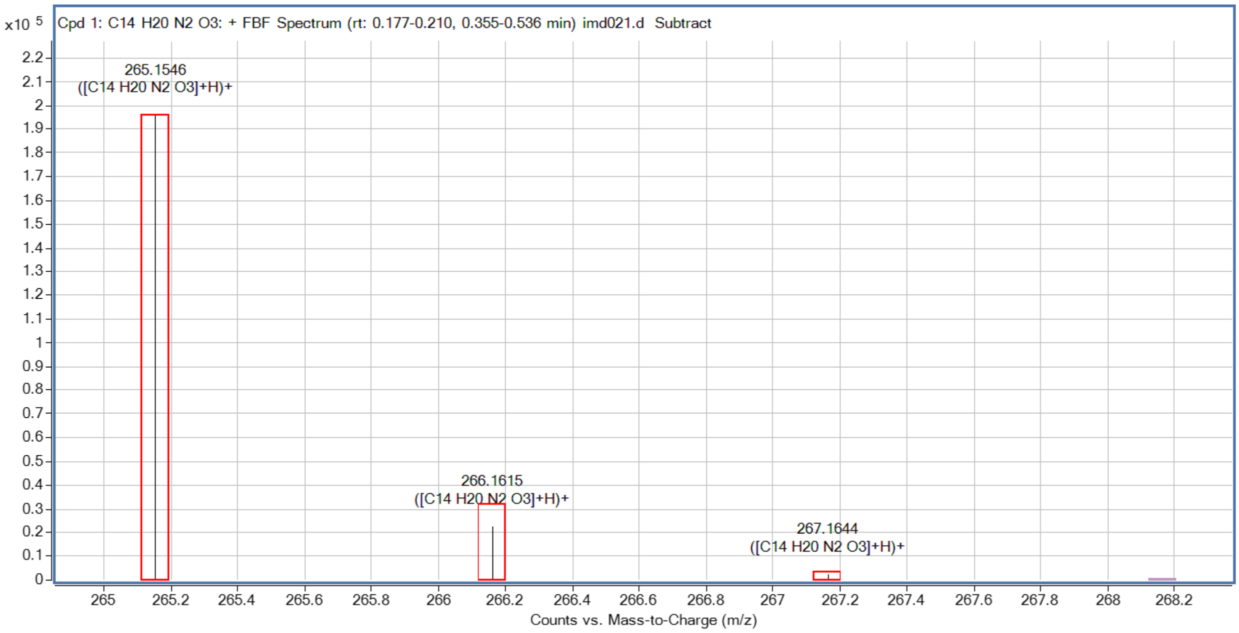

Figure S17. HRMS spectrum of **7d**.

(4*S*,8a*S*)-4-Benzyl-2-cyclohexyldihydropyrrolo[1,2-*a*]pyrazine-1,3,6(2*H*,4*H*,7*H*)-trione, 7e

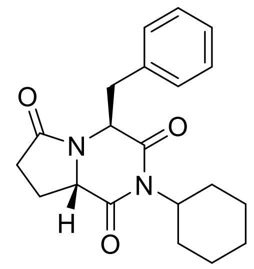

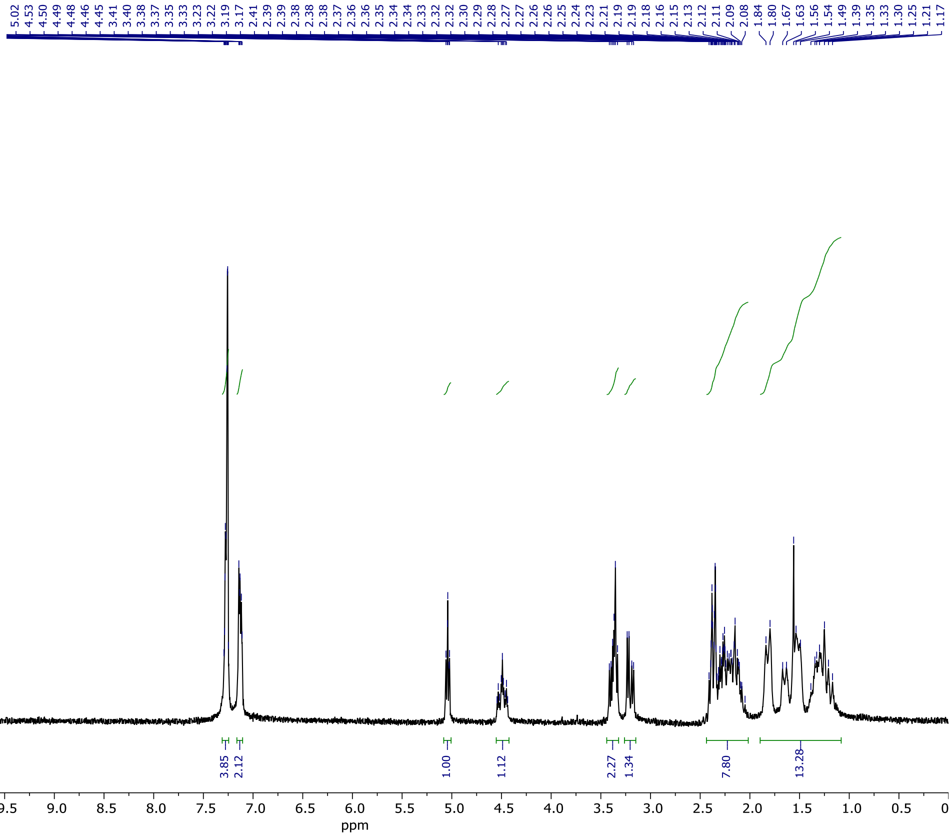

Figure S27. ^{1}H NMR spectrum of **7e** (300 MHz, $CDCl_3$).

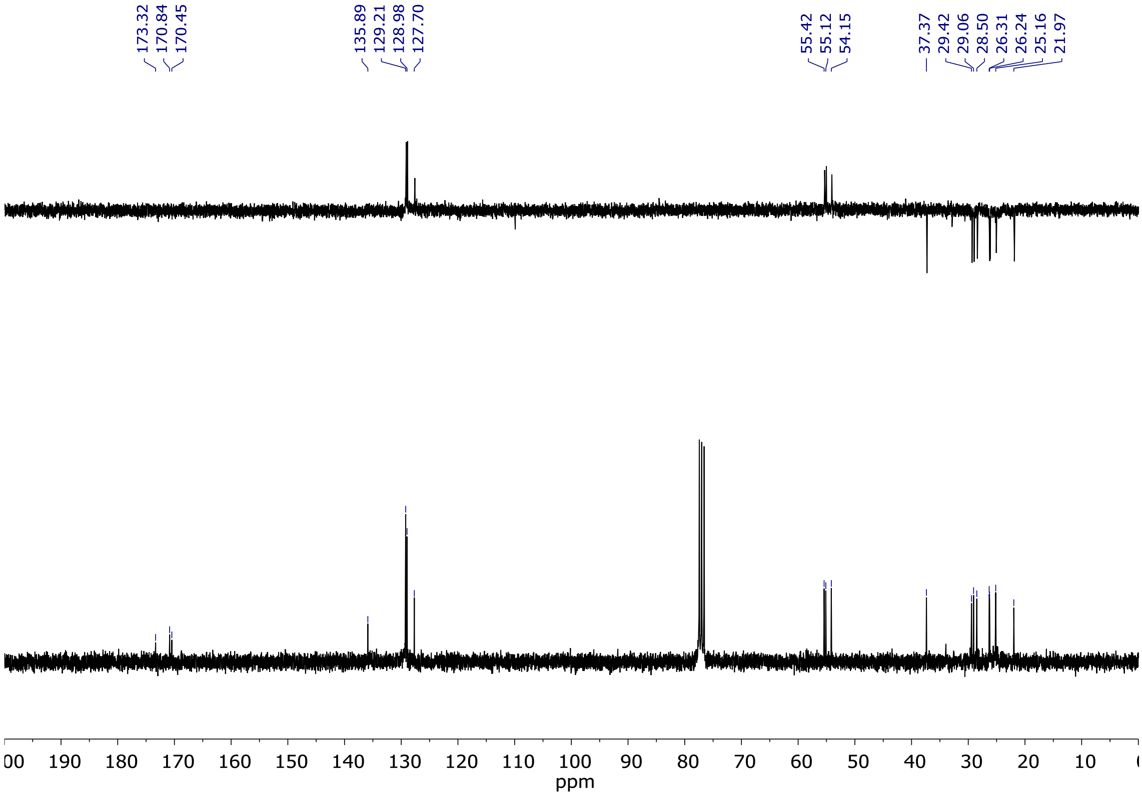

Figure S28. ^{13}C and DEPT NMR spectra of **7e** (75 MHz, $CDCl_3$).

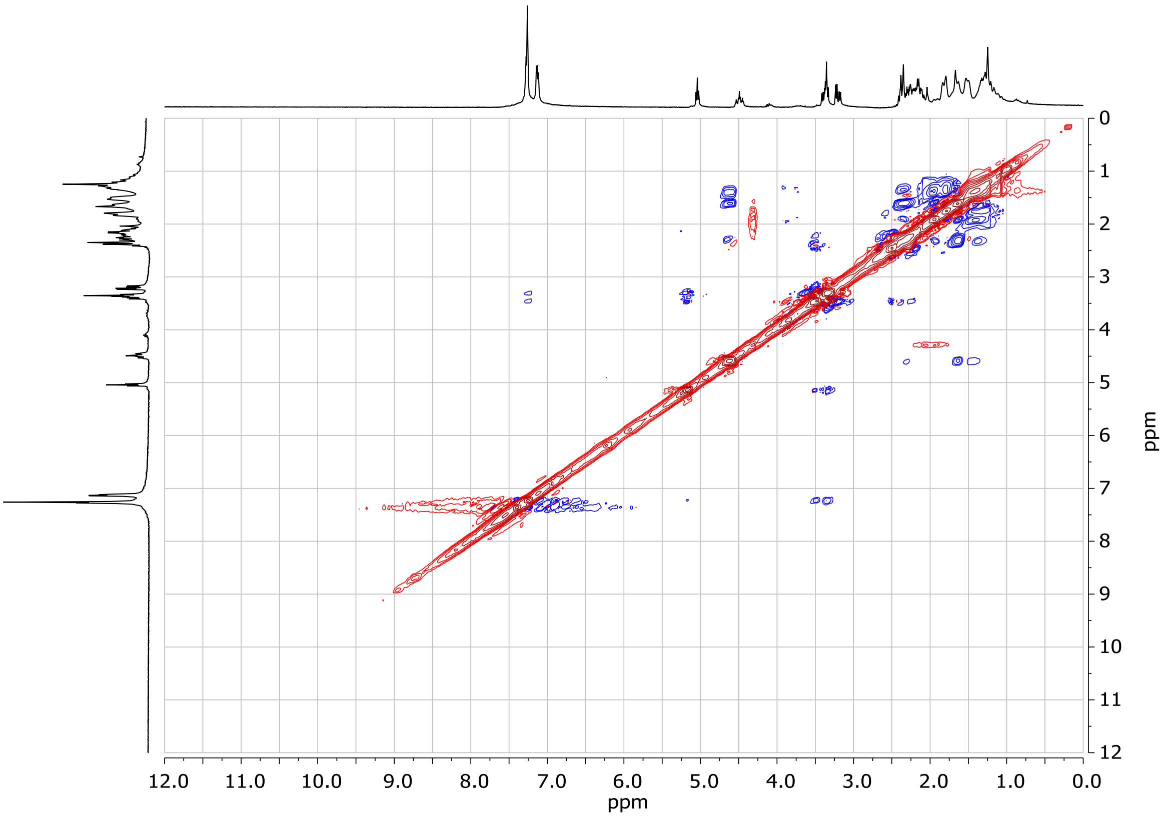

Figure S29. NOESY spectrum of **7e** ($CDCl_3$).

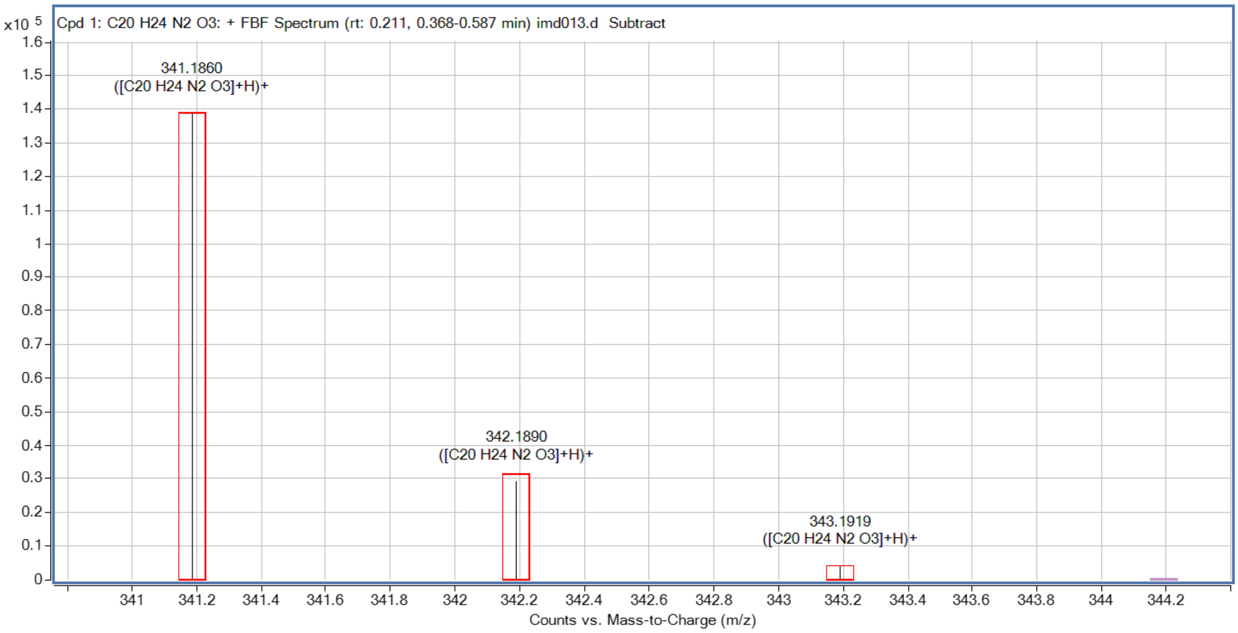

Figure S18. HRMS spectrum of **7e**.

(4*S*,8a*S*)-2-Butyl-4-phenyldihydropyrrolo[1,2-*a*]pyrazine-1,3,6(2*H*,4*H*,7*H*)-trione, 7f

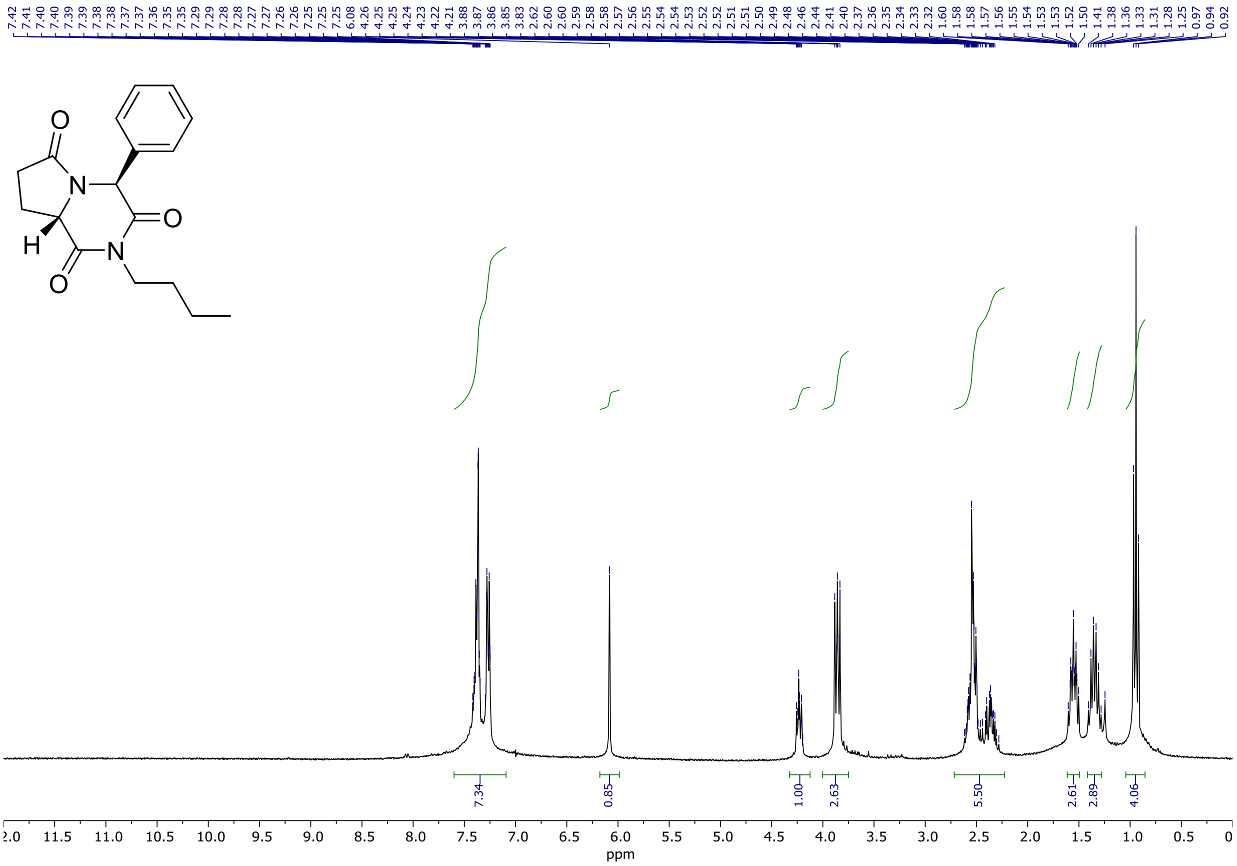

Figure S19. ^{1}H NMR spectrum of **7f** (300 MHz, $CDCl_3$).

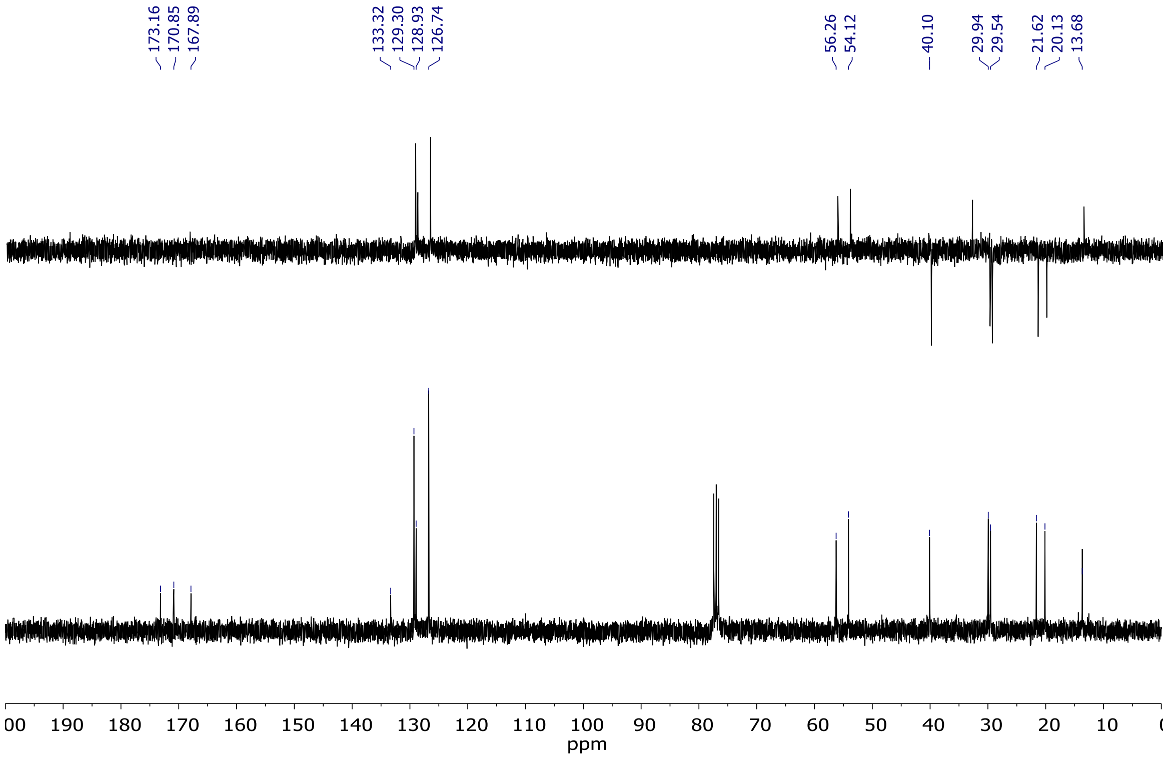

Figure S20. ^{13}C and DEPT NMR spectra of **7f** (75 MHz, $CDCl_3$).

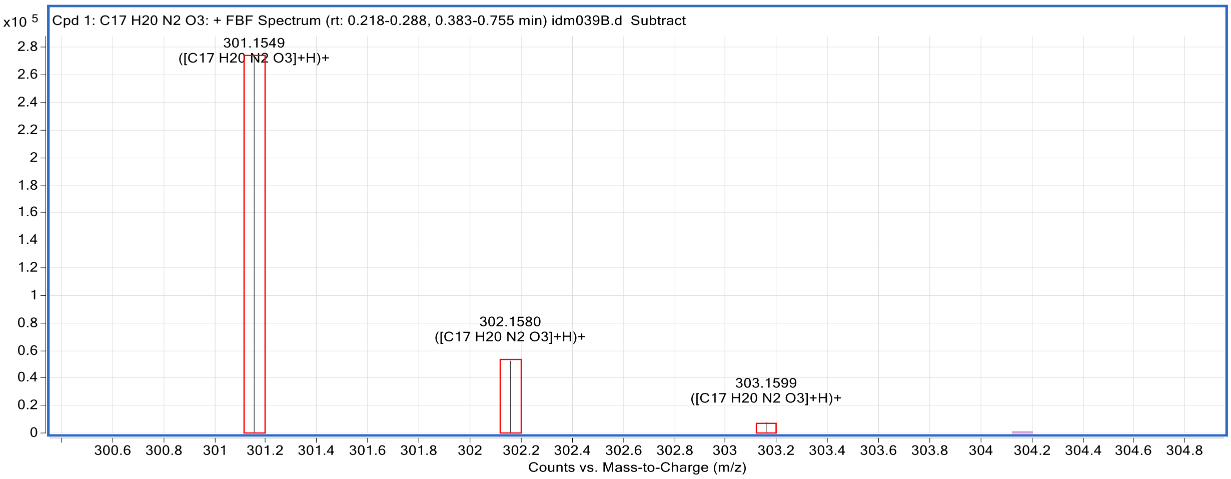

Figure S21. HRMS spectrum of **7f**.

(4*S*,8a*S*)-2-Butyl-4-isopropyldihydropyrrolo[1,2-*a*]pyrazine-1,3,6(2*H*,4*H*,7*H*)-trione, 7g

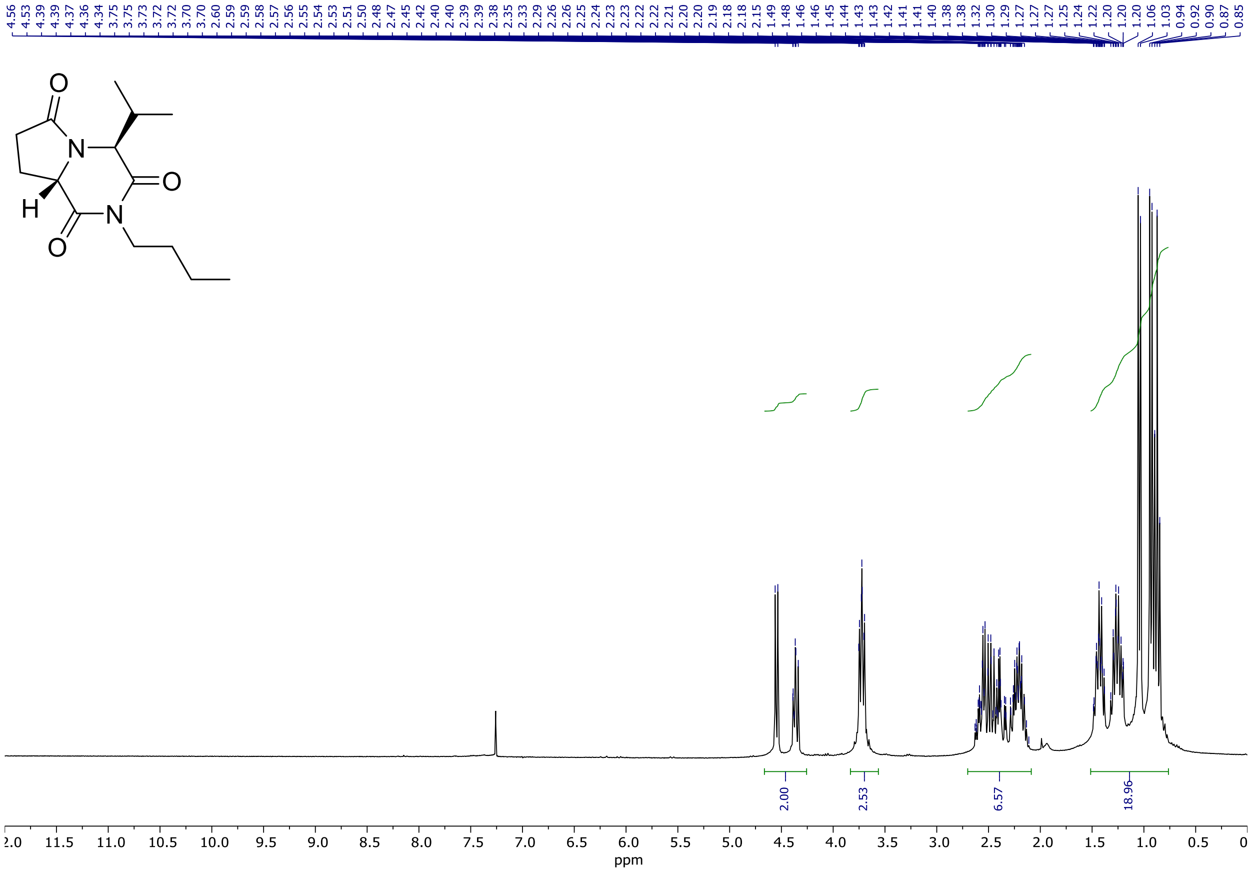

Figure S34. ^{1}H NMR spectrum of **7g** (300 MHz, $CDCl_3$).

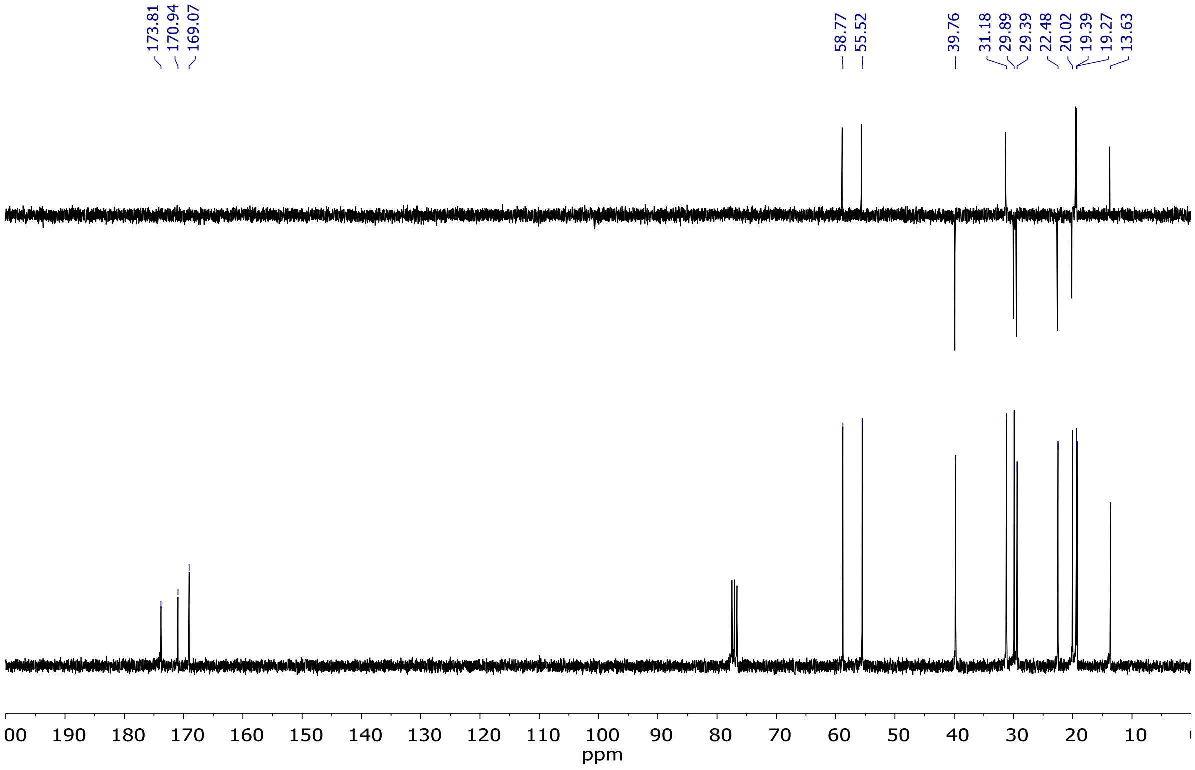

Figure S22. ^{13}C and DEPT NMR spectra of **7g** (75 MHz, $CDCl_3$).

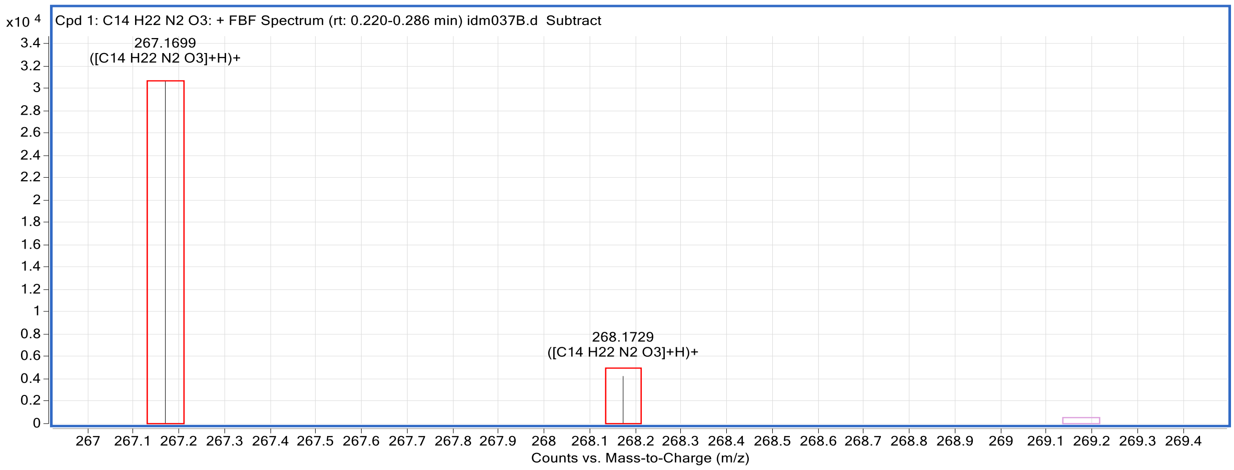

Figure S23. HRMS spectrum of **7g**.

(4*S*,8a*S*)-4-Benzyl-2-butyldihydropyrrolo[1,2-*a*]pyrazine-1,3,6(2*H*,4*H*,7*H*)-trione, 7h

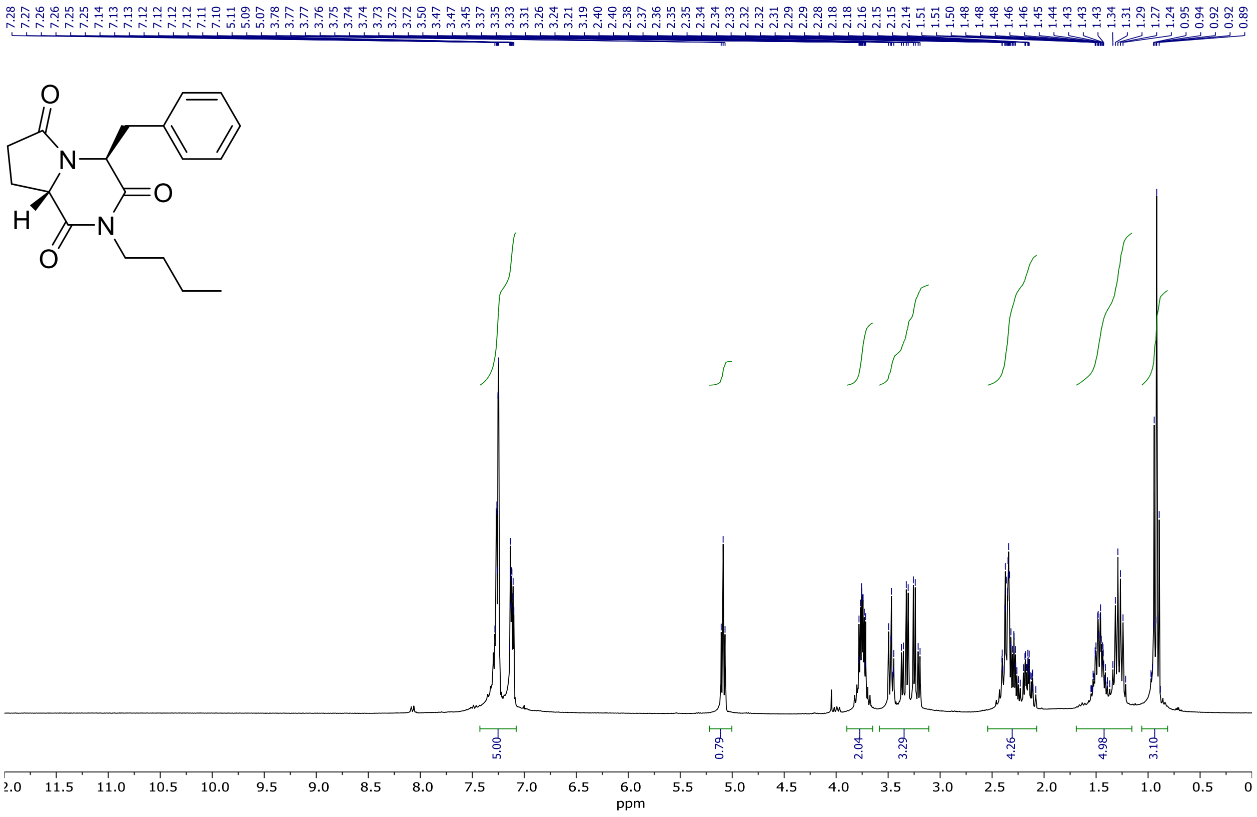

Figure S37. ^{1}H NMR spectrum of **7h** (300 MHz, $CDCl_3$).

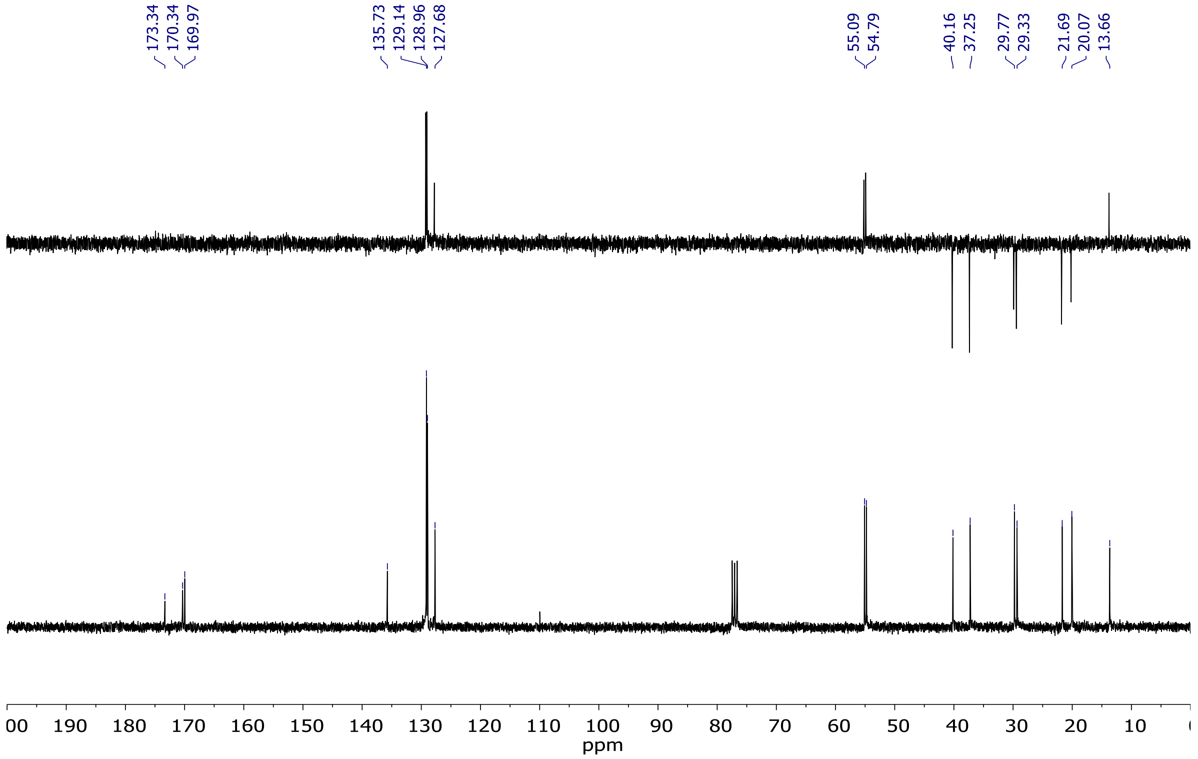

Figure S38. ^{13}C and DEPT NMR spectra of **7h** (75 MHz, $CDCl_3$).

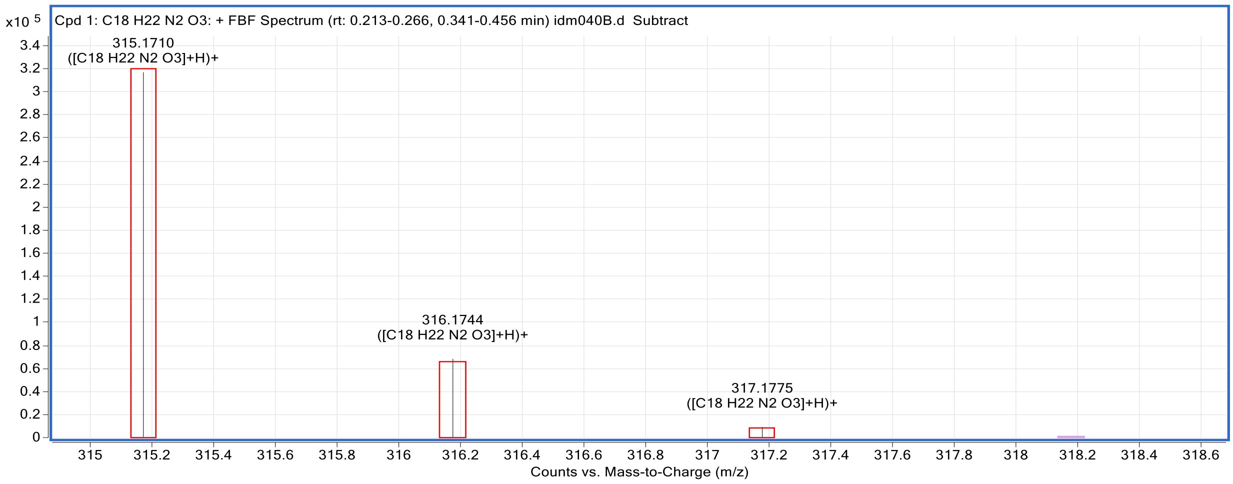

Figure S39. HRMS spectrum of **7h**.

2-Cyclohexyl-7-methylenedihydropyrrolo[1,2-*a*]pyrazine-1,3,6(2*H*,4*H*,7*H*)-trione, 8a

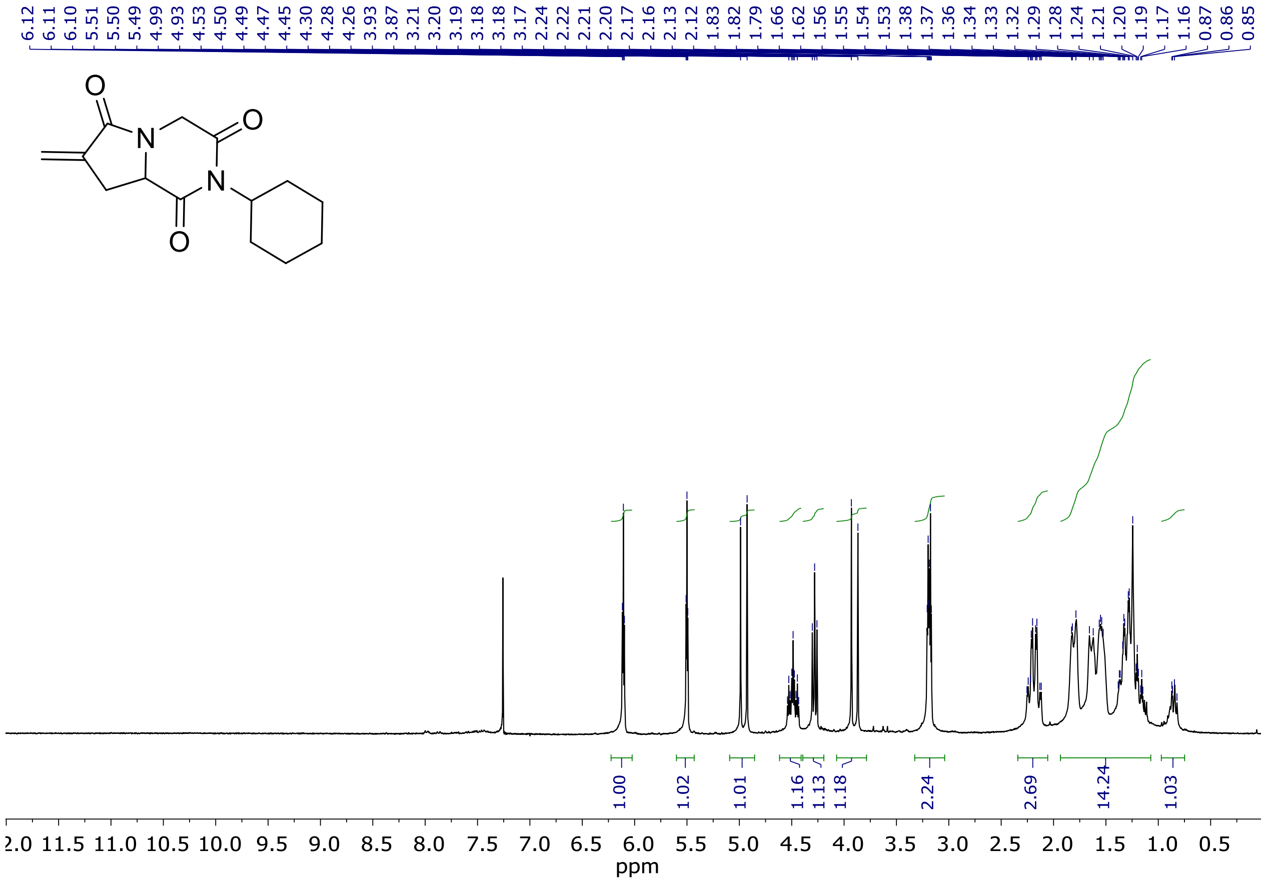

Figure S40. ^{1}H NMR spectrum of **8a** (300 MHz, $CDCl_3$).

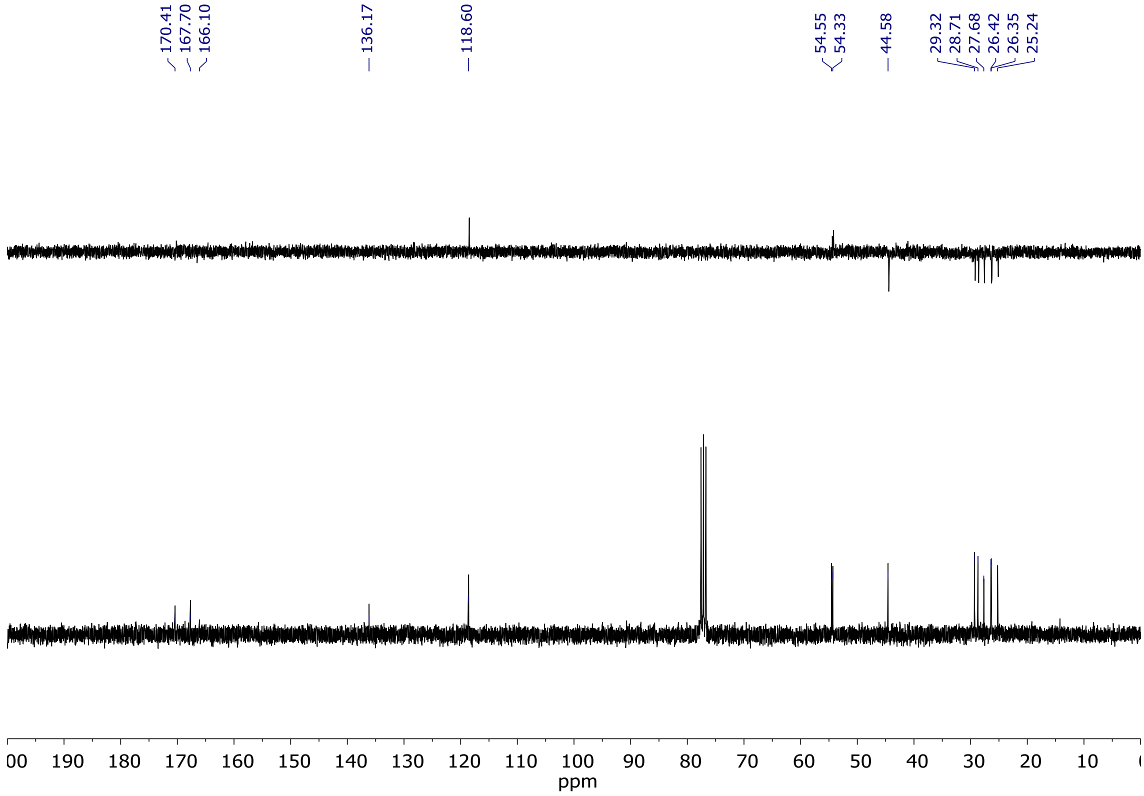

Figure S41. ^{13}C and DEPT NMR spectra of **8a** (75 MHz, $CDCl_3$).

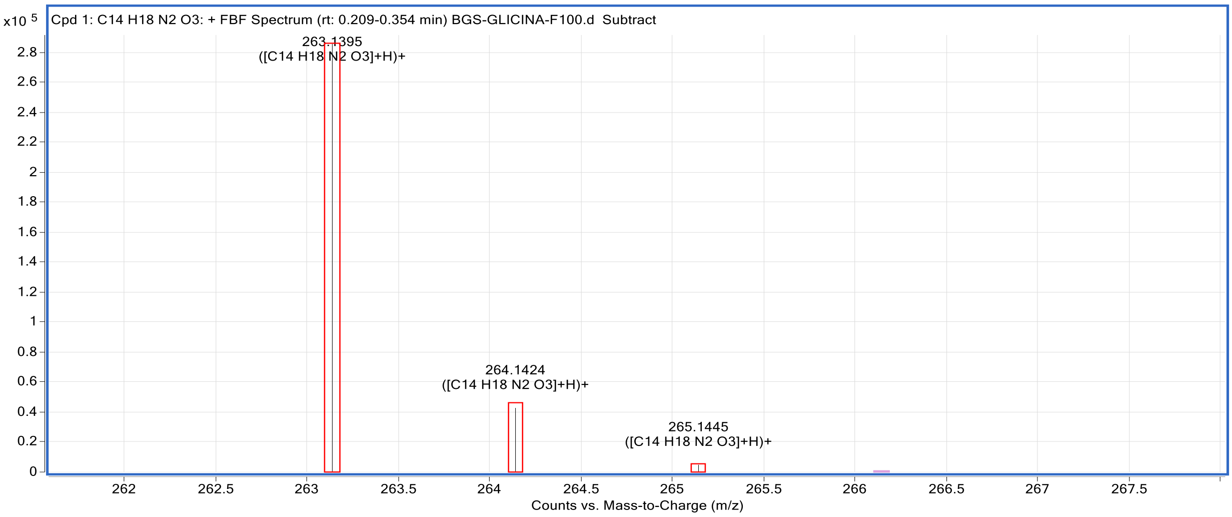

Figure S42. HRMS spectrum of **8a**.

(4*S*,8a*S*)-2-Cyclohexyl-7-methylene-4-phenyldihydropyrrolo[1,2-*a*]pyrazine-1,3,6(2*H*,4*H*,7*H*)-trione, 8b

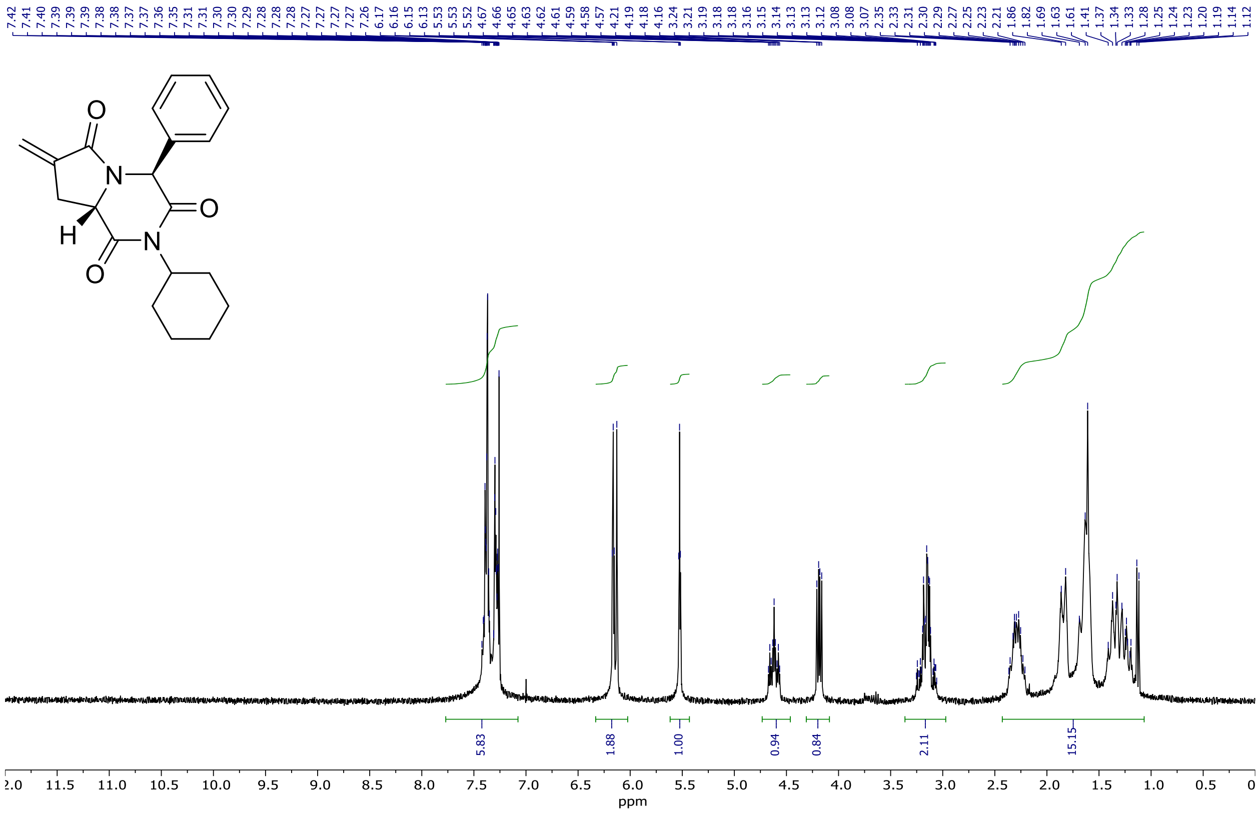

Figure S43. ^{1}H NMR spectrum of **8b** (300 MHz, $CDCl_3$).

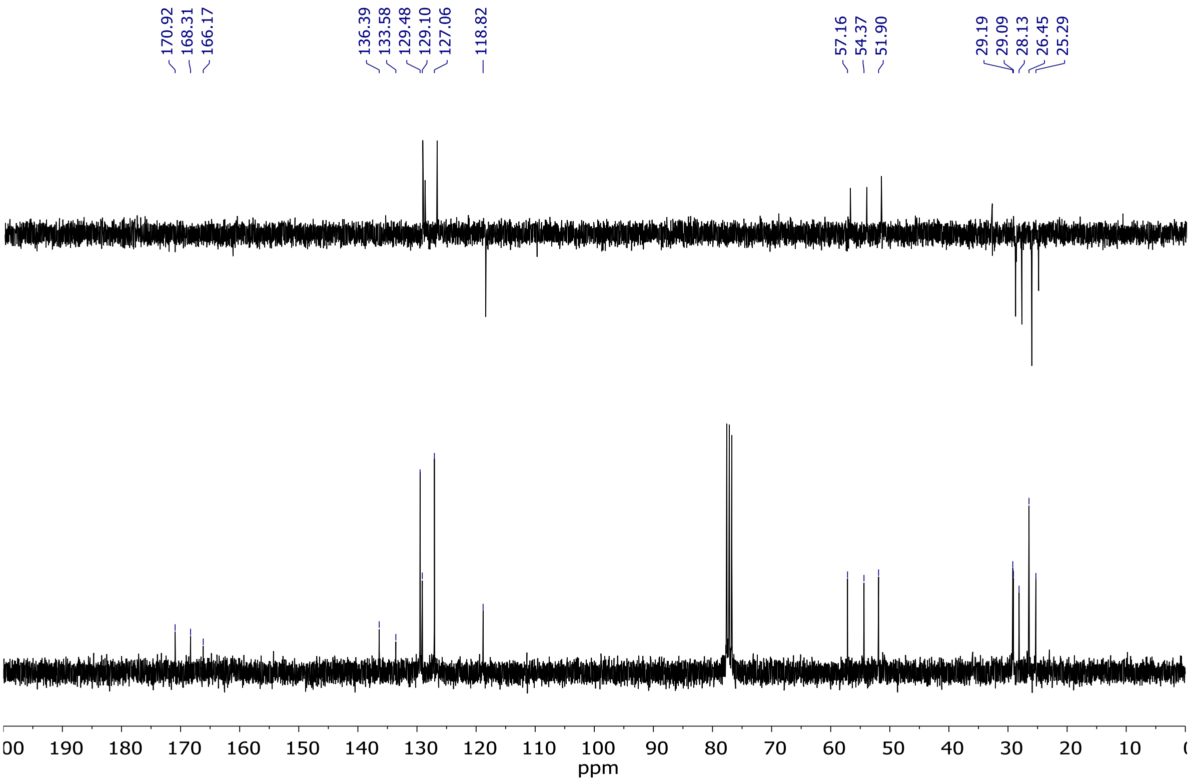

Figure S44. ^{13}C and DEPT NMR spectra of **8b** (75 MHz, $CDCl_3$).

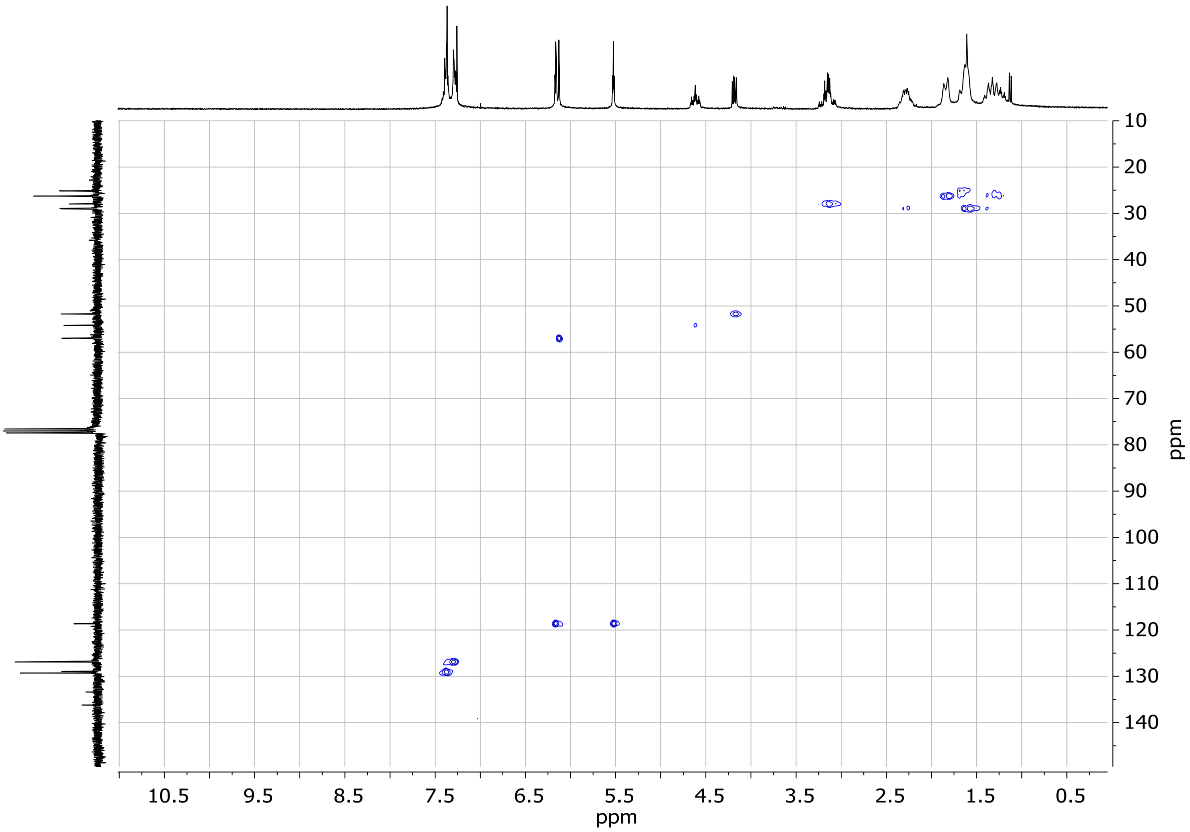

Figure S45. HMQC spectrum of **8b** ($CDCl_3$).

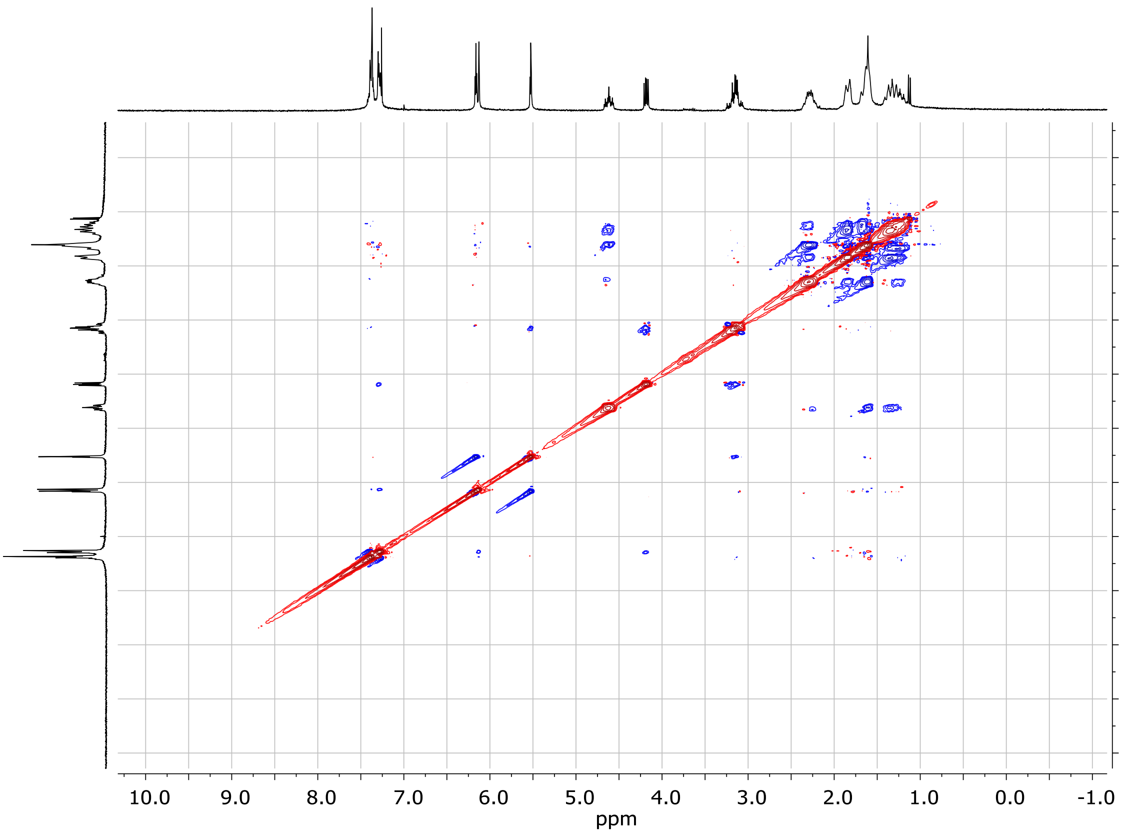

Figure S46. NOESY spectrum of **8b** ($CDCl_3$).

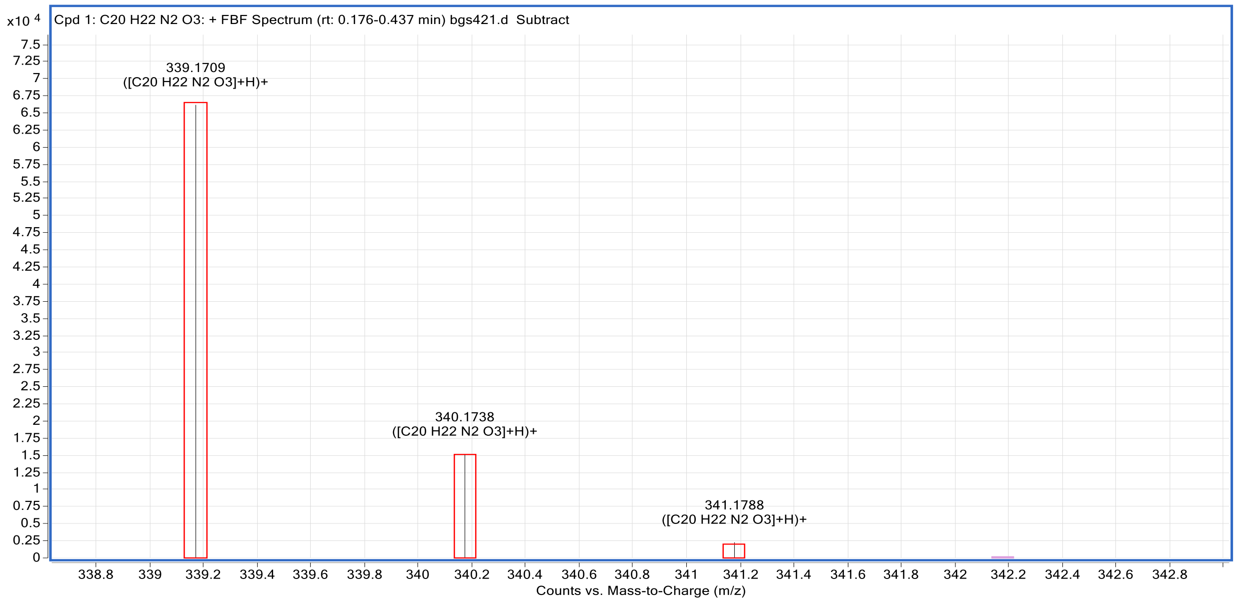

Figure S47. HRMS spectrum of **8b**.

(4*S*,8a*S*)-2-Cyclohexyl-4-isopropyl-7-methylenedihydropyrrolo[1,2-*a*]pyrazine-1,3,6(2*H*,4*H*,7*H*)-trione, 8c

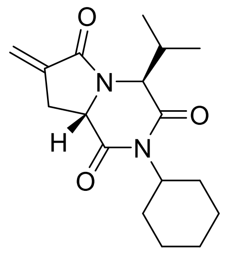

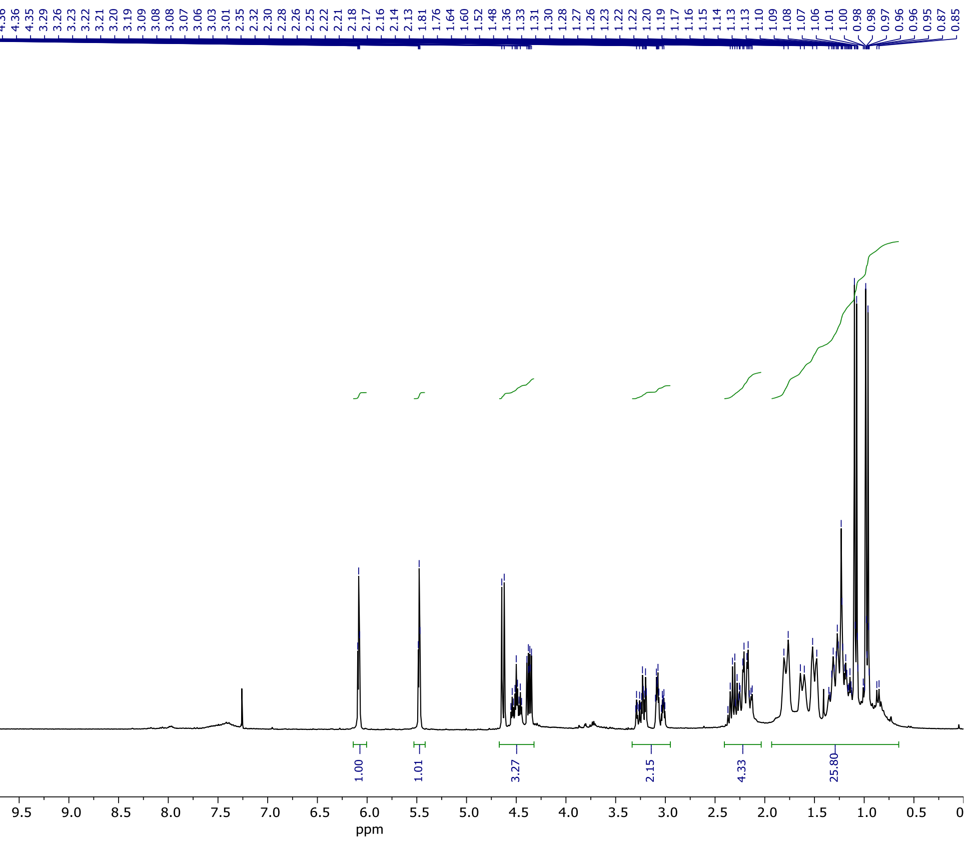

Figure S48. ^{1}H NMR spectrum of **8c** (300 MHz, $CDCl_3$).

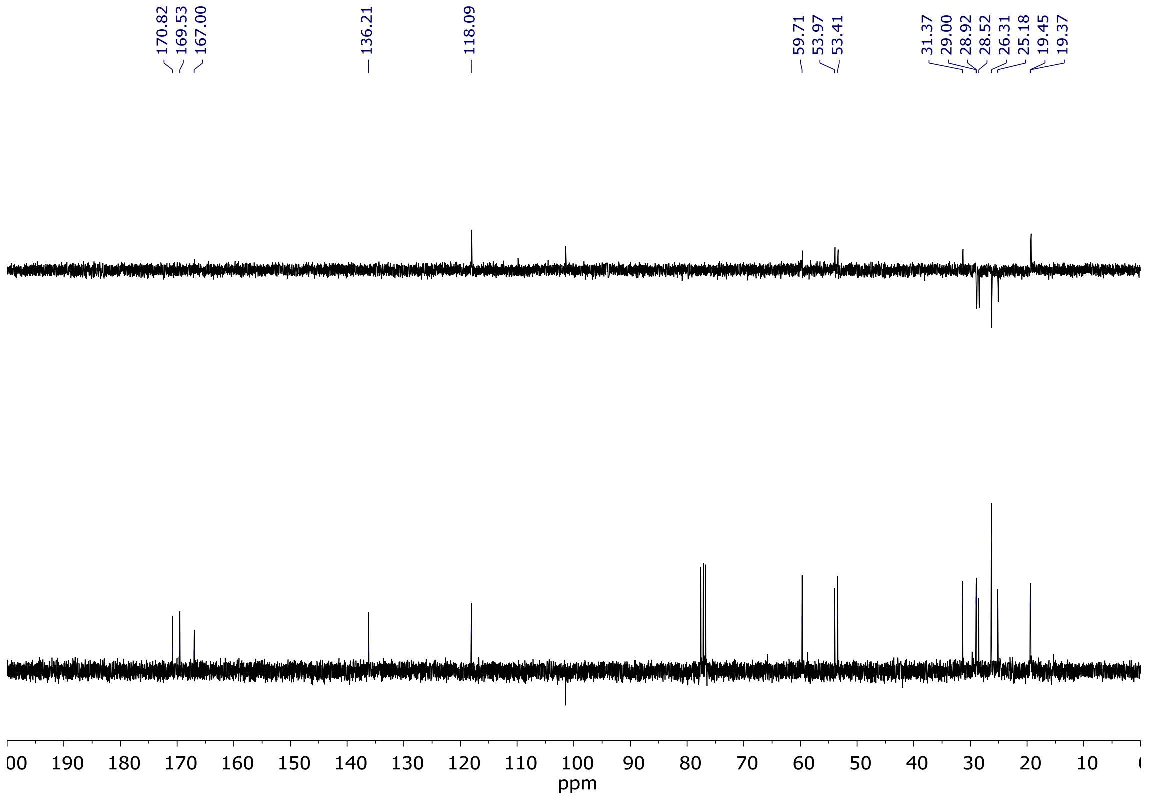

Figure S49. ^{13}C and DEPT NMR spectra of **8c** (75 MHz, $CDCl_3$).

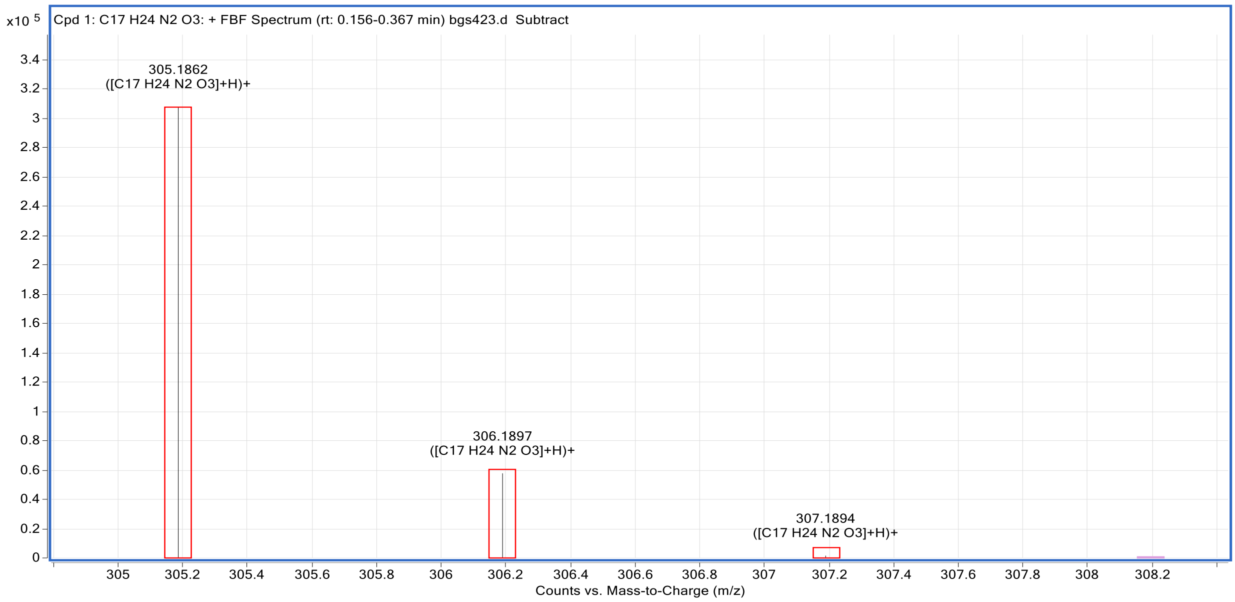

Figure S50. HRMS spectrum of **8c**.

(4*S*,8a*S*)-2-Cyclohexyl-4-methyl-7-methylenedihydropyrrolo[1,2-*a*]pyrazine-1,3,6(2*H*,4*H*,7*H*)-trione, 8d

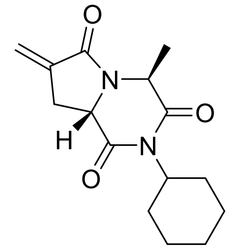

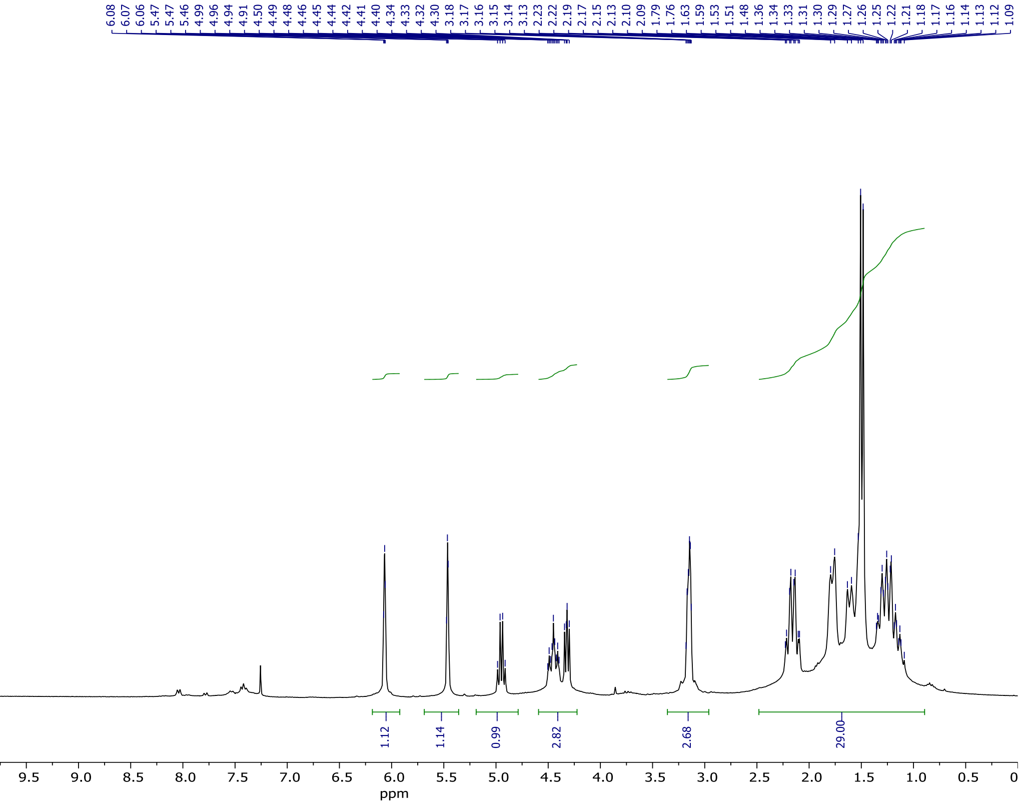

Figure S51. ^{1}H NMR spectrum of **8d** (300 MHz, $CDCl_3$).

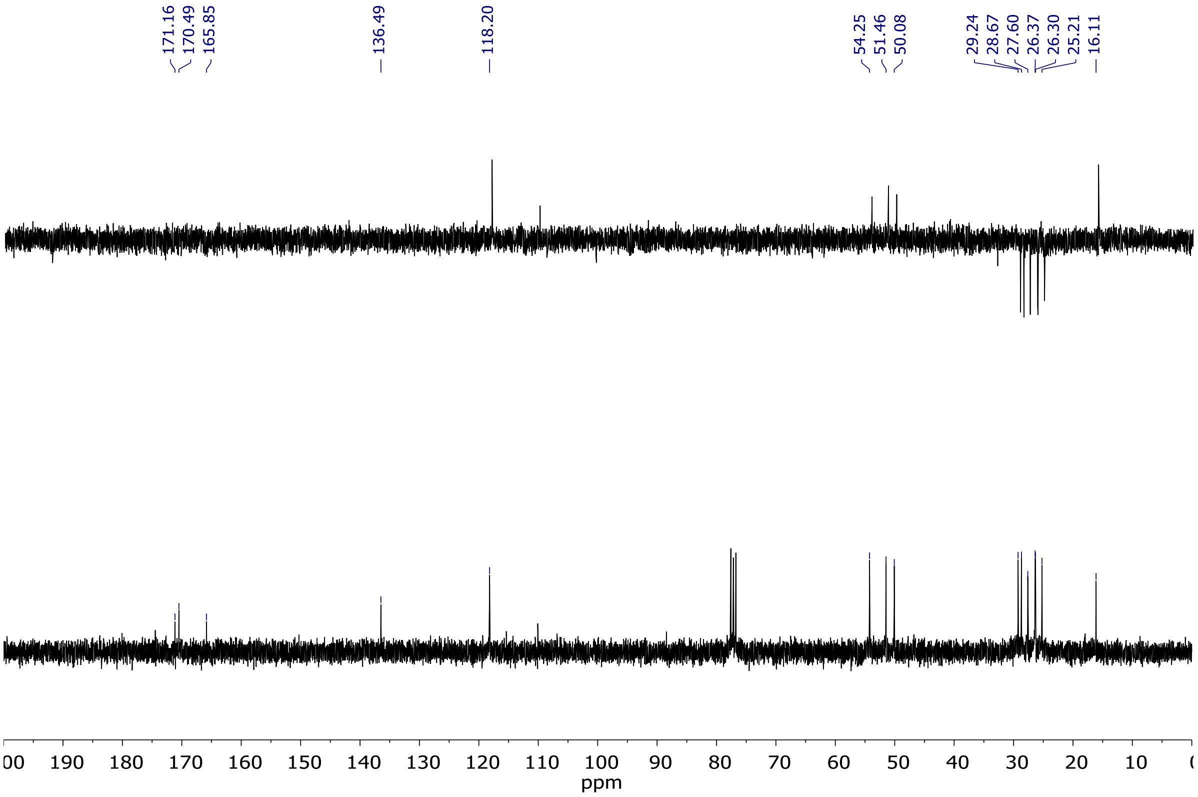

Figure S52. ^{13}C and DEPT NMR spectra of **8d** (75 MHz, $CDCl_3$).

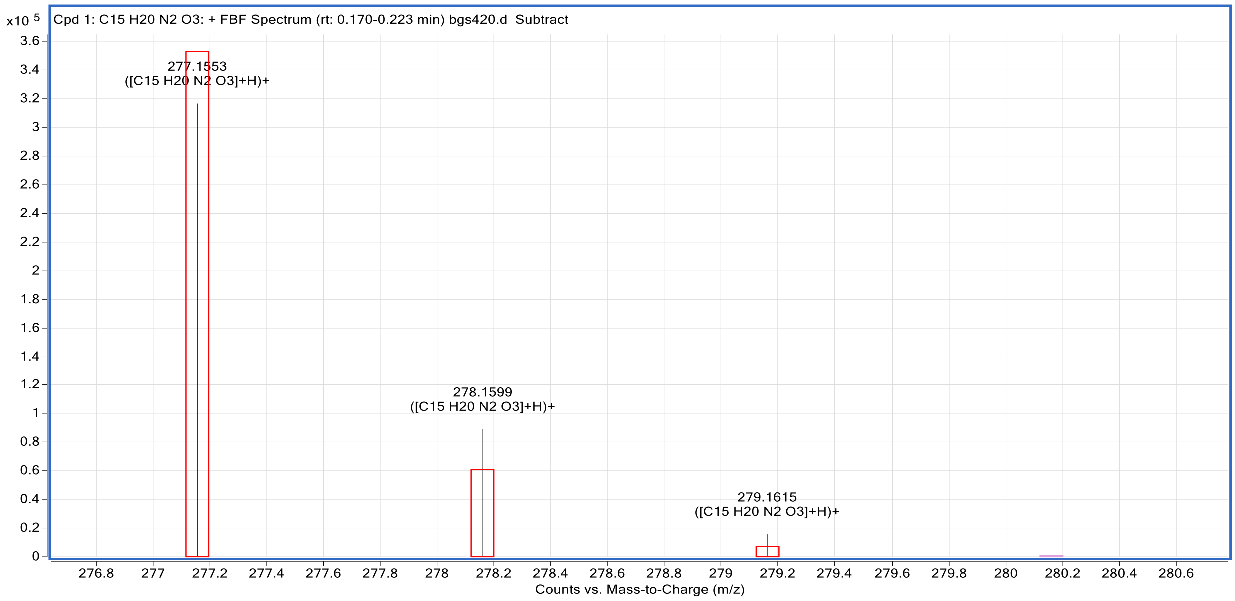

Figure S53. HRMS spectrum of **8d**.

(4*S*,8a*S*)-4-Benzyl-2-cyclohexyl-7-methylenedihydropyrrolo[1,2-*a*]pyrazine-1,3,6(2*H*,4*H*,7*H*)-trione, 8e

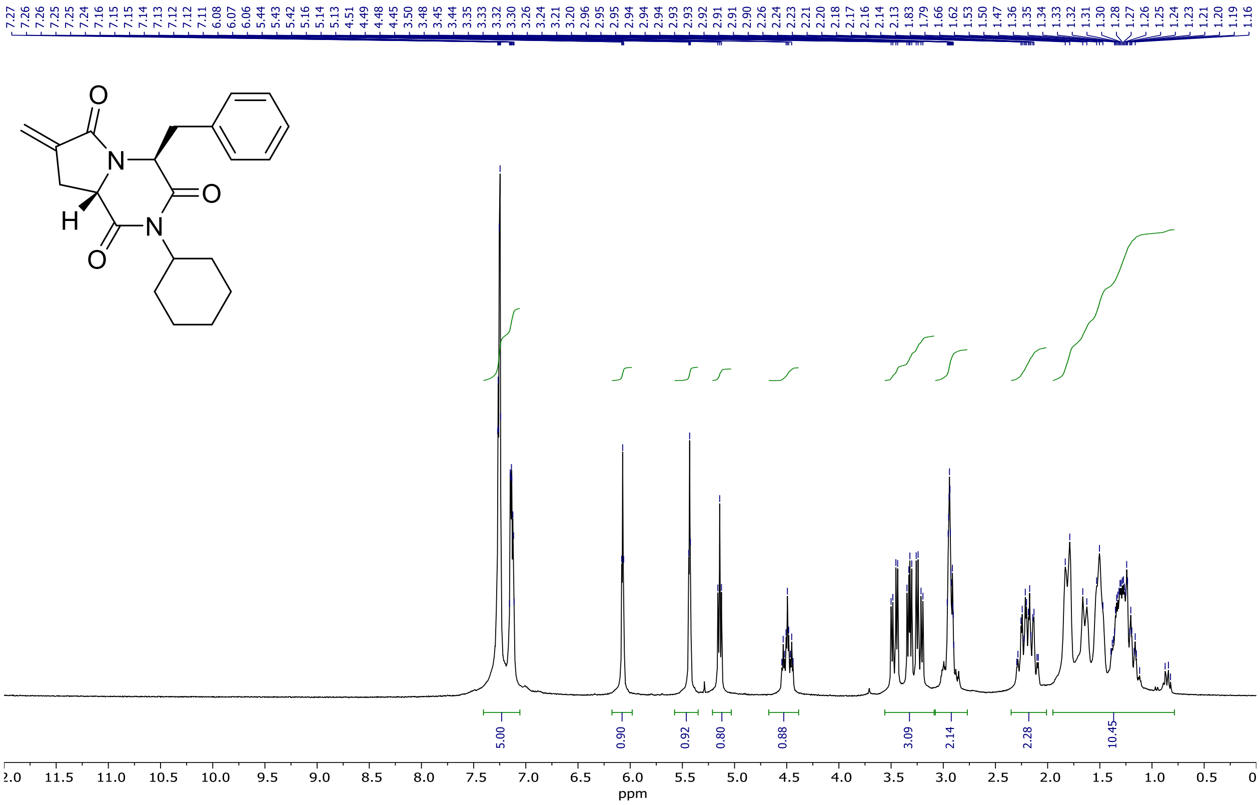

Figure S54. ^{1}H NMR spectrum of **8e** (300 MHz, $CDCl_3$).

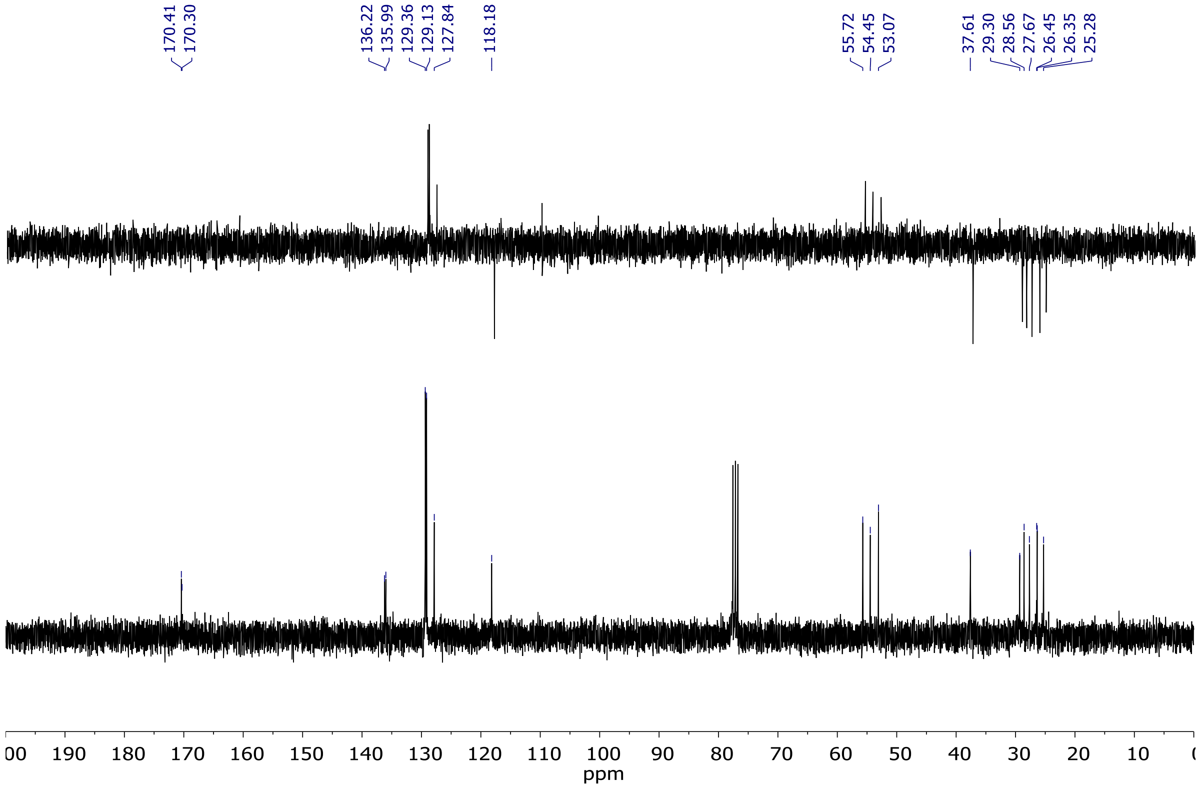

Figure S55. ^{13}C and DEPT NMR spectra of **8e** (75 MHz, $CDCl_3$).

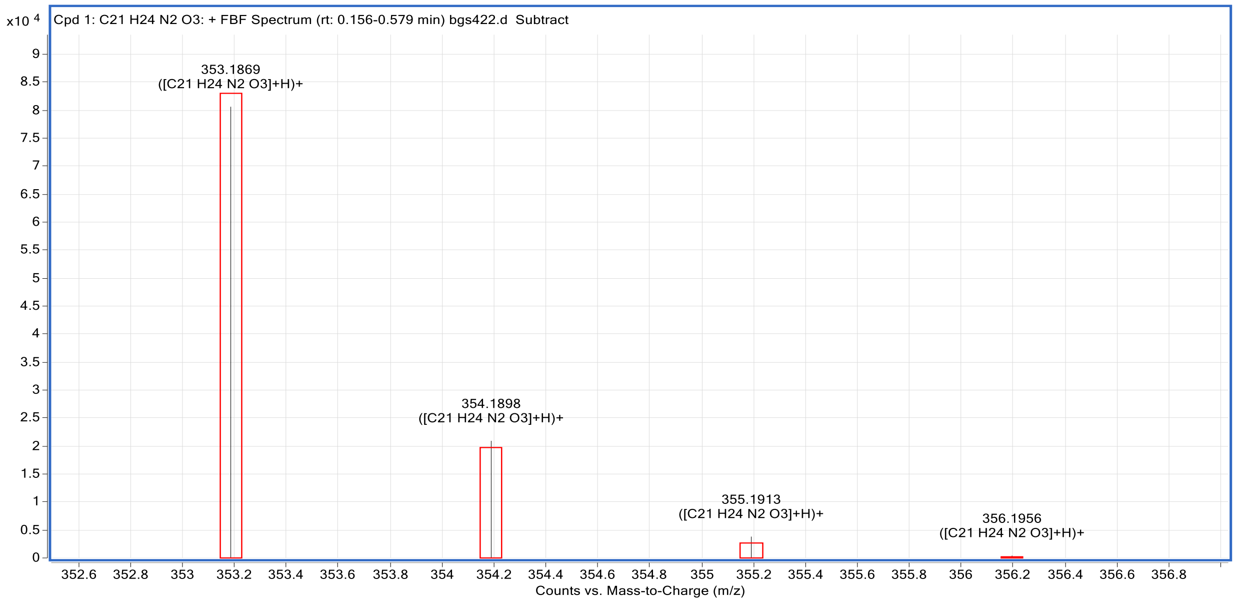

Figure S56. HRMS spectrum of **8e**.

X-Ray diffraction studies

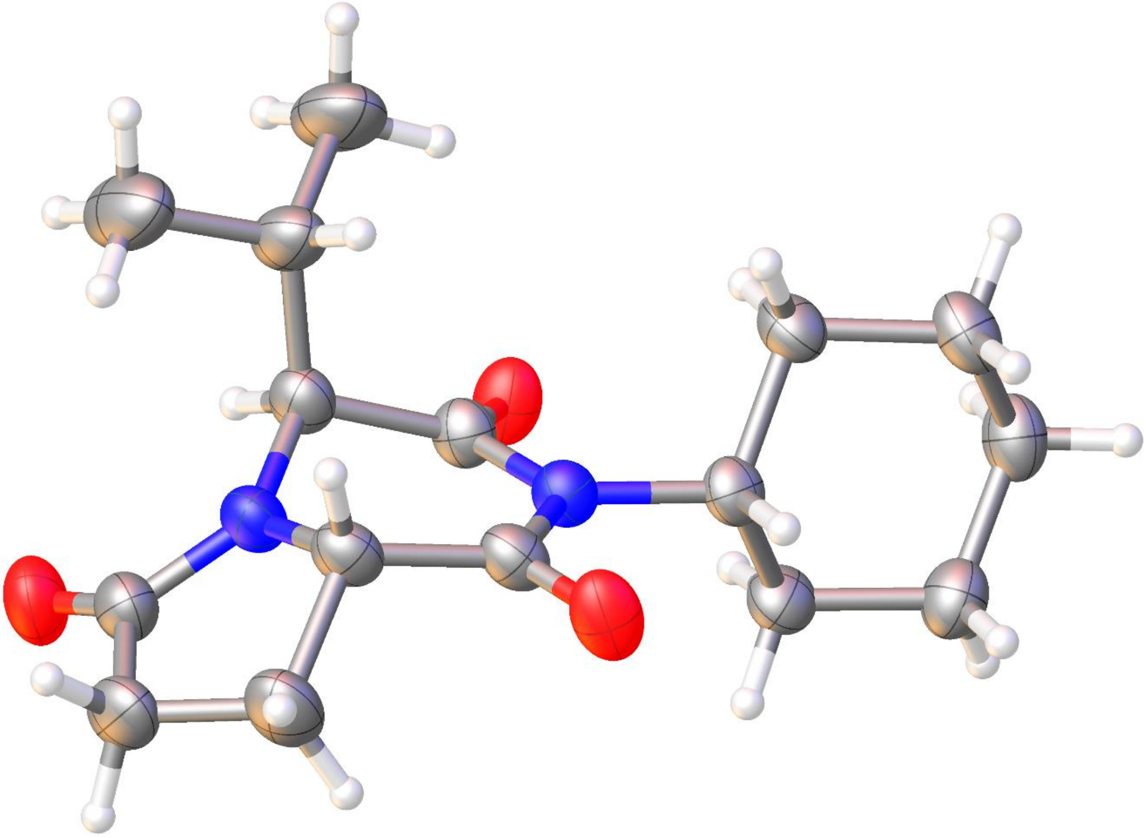

Figure S57. X-ray molecular structure of compound **7c**. The Olex2 plot is at the 30% probability level.

Single crystals of compound **7c** were obtained by slow evaporation of a solution of the isolated compound in a 2:1 methanol:water mixture. Crystal data and details on data collection and refinement are summarized in **Table S1**. The structure was drawn with the Olex2 program.[1]

Three dimensional X-ray data were collected on a Bruker D8 VENTURE diffractometer. Data were corrected for absorption effects using the multi-scan method (SADABS).[2] Complex scattering factors were taken from the SHELXL-2016[3] program running under the WinGX program system[4] as implemented on a Pentium® computer. The structure was solved with SIR92[5] and refined by full-matrix least-squares on F^2. All hydrogen atoms were included in calculated positions and refined in riding mode. Refinement converged with anisotropic displacement parameters for all non-hydrogen atoms.

---

1 Olex2: Dolomanov, O. V.; Bourhis, L. J.; Gildea, R. J.; Howard, J. A. K.; Puschmann, H. *J. Appl. Cryst.* **2009**, *42*, 339-341.

2 SADABS: Krause, L.; Herbst-Irmer, R.; Sheldrick, G. M.; Stalke, D. *J. Appl. Cryst.* **2015**, *48*, 3-10.

3 SHELX-2016: Sheldrick, G. M. *Acta Cryst*. **2008**, *A64*, 112-122.

4 WinGX: Farrugia, L. J. *J. Appl. Cryst*. **1999**, *32*, 837-838.

5 SIR92: Altomare, A.; Cascarano, G.; Giacovazzo, C.; Guagliardi, A.; Burla, M. C.; Polidori, G., Camalli, M. *J. Appl. Cryst.* **1994**, *27*, 435-435.

Table S1. Crystal data and refinement details for **7c**.

Empirical formula	$C_{16}H_{24}N_2O_3$
MW	292.37
crystal system	Monoclinic
space group	$P\ 2_1$
T/K	299(2)
a/Å	5.2417(6)
b/Å	9.0213(10)
c/Å	17.3575(19)
α/deg	90
β/deg	91.763(6)
γ/deg	90
V/Å^3	820.39(16)
F(000)	316
Z	2
λ, Å	1.54178
D_{calc}/g cm^{-3}	1.184
μ/mm^{-1}	0.661
θ range/deg	7.66 – 65.92
R_{int}	0.0603
reflections measured	5817
unique reflections	2672
reflections observed	2392
GOF on F^2	1.058
$R1^a$	0.0792
$wR2^b$	0.2293
Largest ≠ peak & hole/eÅ^{-3}	0.553 and -0.250

^{a} $R1 = \sum||F_0|-|Fc||/\sum|F_0|$. ^{b} w$R$2 (all data) = $\{\sum[w(|F_0|^2-|Fc|^2)^2]/\sum[w(F_0^4)]\}^{1/2}$

Computational study

Gibbs' free energies in Hartree of epimers of **7c** on C8a (gas phase) at the B3LYP/6-31G** level

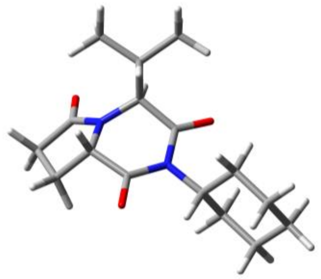

(4*S*,8a*S*)-**7c**

-959,089552

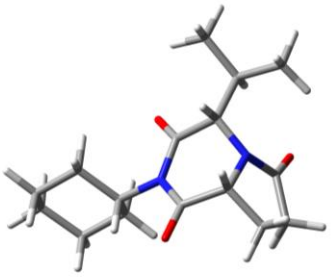

(4*S*,8a*R*)-**7c** (*epi*-**7c**)

-959,081550

Gibbs' free energies in Hartree of epimers of **8c** on C8a (gas phase) at the B3LYP/6-31G** level

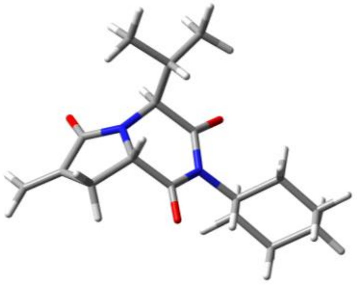

(4*S*,8a*S*)-**8c**

-997,171465

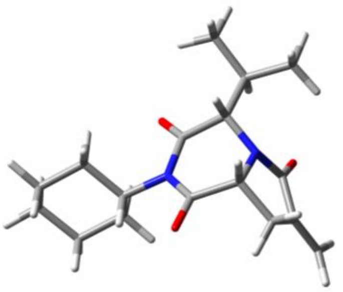

(4*S*,8a*R*)-**8c** (*epi*-**8c**)

-997,163195
